# Identification of breadfruit (*Artocarpus altilis*) and South American crops introduced during early settlement of Rapa Nui (Easter Island), as revealed through starch analysis

**DOI:** 10.1371/journal.pone.0298896

**Published:** 2024-03-20

**Authors:** Paloma Berenguer, Claudia Clavero, Mónica Saldarriaga-Córdoba, Antonio Rivera-Hutinel, Daniela Seelenfreund, Helene Martinsson-Wallin, Patricia Castañeda, Andrea Seelenfreund

**Affiliations:** 1 Escuela de Antropología, Geografía e Historia, Facultad de Ciencias Sociales Universidad Academia de Humanismo Cristiano, Santiago, Chile; 2 Facultad de Ciencias Básicas, Universidad Metropolitana de Ciencias de la Educación, Ñuñoa, Chile; 3 Centro de Investigación en Recursos Naturales y Sustentabilidad (CYRENIS), Universidad Bernardo O’Higgins, Santiago, Chile; 4 Facultad de Ciencias Químicas y Farmacéuticas, Universidad de Chile, Santiago, Chile; 5 Department of Archaeology and Ancient History, Uppsala University, Uppsala, Sweden; The University of the West Indies, TRINIDAD AND TOBAGO

## Abstract

Starch residue analysis was carried out on stone tools recovered from the bottom layer of the Anakena site on Rapa Nui (Easter Island). These deposits have been dated to AD 1000–1300 AD and so far, represent the earliest evidence of human settlement on this island. Twenty obsidian tools were analyzed. Analysis of 46 starch grains recovered from 20 obsidian tools from the earliest dated level of the Anakena site on Rapa Nui provides direct evidence for translocation of traditional crop plants at initial stages of the colonization of this island. The analysis of starch grains was based mainly on statistical methods for species identification but was complemented by visual inspection in some cases. Our results identify taxons previously unknown to have been cultivated on the island, such as breadfruit (*Artocarpus altilis*), *Zingiber officinale* (ginger), and starch grains of the *Spondias dulcis* and *Inocarpus fagifer* tropical trees. Additionally, starch grains of *Colocasia esculenta* (taro) and *Dioscorea* sp. (yam), both common species in Pacific agriculture, were identified. Furthermore, the presence of four American taxa *Ipomoea batatas* (sweet potato), *Canna sp*. (achira), *Manihot esculenta* (manioc), and *Xanthosoma* sp., was detected. The occurrence of *Canna sp*., *M*. *esculenta*, and *Xanthosoma* sp. starch grains suggests the translocation of previously not described South American cultivars into the Pacific. The detection of *I*. *batatas* from this site in Rapa Nui constitutes the earliest record of this cultigen in the Pacific. Our study provides direct evidence for translocation of a set of traditional Polynesian and South American crop plants at the initial stages of colonization in Rapa Nui.

## Introduction

The study of the colonization of Remote Oceania revolves around some fundamental questions, such as the timing, the routes taken by the voyagers, and the origins and composition of the intentionally translocated set of animal and plant species. The question of timing is still under debate, as recent data shows a much later timing for all colonization events than previously posed [1–6]. As several authors [7–9] have pointed out, colonization of remote islands requires several stages to be successful: discovery, with initial location and exploration of the new land and its resources, followed by colonization, involving the settlement of a resident population with its domesticates to ensure long-term survival. The time span between the first and second stages probably required return voyages, as handed down in traditional legends. For example, the oral history of Rapa Nui regarding the planning stage of the new settlement is very specific, and states that it involved at least one return voyage [10, 11]. The process of colonization of the numerous islands of East Polynesia and Marginal East Polynesia [12] spread over the vast expanse of the Pacific Ocean involved the intentional transport of several species, as the islands become increasingly impoverished in natural resources from west to east. Genetic studies of all species transported intentionally (or not) on the canoes into Remote Oceania from widely different origins, such as humans, animals, plants, and microorganisms, have revealed that this was a complex process. Polynesians were great navigators, and for several centuries they crossed the ocean in all directions, moving plants and other goods. Over 70 different plant species were purposely introduced into the Pacific. Some of these originate in mainland Asia, others can be traced to Southeast Asia, while others are native to New Guinea [13]. Approximately 50 of these species were collected or described by the first European expeditions that visited the islands [14]. It is probable that the process of establishing the new crops and hence, generating a new productive landscape, began soon after settlement [e.g. 15–18].

Microfossil and starch analyses have provided evidence for the presence of plant taxa in archaeological contexts [19]. Some of these studies have specifically addressed the introduction of canoe plants into Remote Oceania, and particularly into Marginal East Polynesia. Prebble et al. [20] have identified the presence of tropical and subtropical East Polynesian plants, such as taro and other leafy crop plants, using fossil pollen data, and the presence of fossil remains of commensal invertebrates common in tropical garden contexts in the more temperate offshore islands of New Zealand. Using starch analyses, Allen and Ussher [21] were able to document the presence of a number of West Polynesian taxa in the archaeological record of the Marquesas Islands during the 14th century, such as breadfruit and kava, but also of sweet potato. The authors were also able to demonstrate significant shifts over time related to the importance of each of these crops.

Most of the research aimed at the identification of Rapa Nui’s plant and animal species have focused on the paleoecological history of the island, addressing questions regarding a supposed ecological collapse [1, 22–24], the causes of deforestation [1, 25–27], the date and origin of the initial human settlement [6, 28–31], and the reconstruction of the prehistoric landscape [e.g., 32–40]. Some of these studies have also addressed the general importance of the available food resources [see for example, 41–47].

Flenley [32] did pioneering work in reconstructing the paleoecological history of Rapa Nui. His work was based on pollen analysis of soil sediments taken from inside the main crater lakes of the island. He demonstrated that before human settlement, the island was populated by several tree species, such as the toromiro (*Sophora toromiro*), and a type of palm (*Paschalococus* sp.), some species of the Asteraceae and Coprosma families, and indicated that deforestation began at around 1200 AD. The precise timing of the first permanent human settlement of Easter Island remains uncertain [30, 48], as are the plant species introduced by the first settlers. The first stages of human settlement were marked by the clearing of the original forest cover for agriculture plots around 1200 AD [25, 35, 49], in time replacing the endemic forest with an artificial, imported agricultural landscape [36, 50]. Whistler [14] estimates that only 12 species were introduced in southeastern Polynesia by these first settlers. Among these species were some of the most common Polynesian trees, such as *Alphitonia* cf., *Spondias dulcis* (Tahitian apple or vī), *Inocarpus fagifer* (Tahitian chestnut), *Casuarina esquistetifolia* (ironwood), and *Artocarpus altilis* (breadfruit tree). Of these, only *A*. *altilis* grows today on the island; however, it is a modern introduction. Additionally, *Lagenaria siceraria* (bottle gourd*)* and *Ipomoea batatas* (sweet potato*)* were introduced from the South American continent [51, 52]. Other authors suggest that about 20 of the commonly available species introduced and cultivated in the Western and Central Pacific reached as far east as Easter Island [13]. Archaeological charcoal analyses indicate the presence of taxa characteristic of a mesic forest at a period between the fourteenth and the beginning of the seventeenth century, which included large trees, such as *Pittosporum*, *Alphitonia* cf., *Syzygium* cf., and *S*. *malaccense*, among others [53].

Sediment analysis performed in different areas of Rapa Nui have provided insight into the plant species that were prehistorically cultivated on the island. Pollen and phytoliths analyses of soil sediments have allowed the identification of Ti (*Cordyline fruticosa*), sweet potato, a Moraceae (possibly *Broussonetia papyrifera*), and a type of banana (*Musa* sp.) [54]. Horrocks and Wozniak [55] analyzed microfossils collected from soil sediments at Ahu Te Niu, dated roughly between the 13th and 15th centuries AD, and identified starch grains, which they cautiously identified as yam (*Dioscorea alata*), sweet potato, taro (*Colocasia esculenta*), and bottle gourd. They suggest that the area was subject to a mixed dryland agriculture production system, dominated by yam and sweet potato, and supplemented with taro and bottle gourd. Additional insight derives from the Ahu Heki’i and La Pérouse sites on the northeast coast [54] and from the craterlake sediments inside the Rano Kau [36] and Rano Raraku craters [50]. Here, through microfossil analyses the authors identified taro, sweet potato, banana, and possibly bottle gourd, providing evidence of ancient Polynesian agriculture in these localities [50]. Sherwood et al. [56], based on excavations performed in the inner Moai quarry at Rano Raraku suggest that the inner, south, and east slopes of Rano Raraku were cultivated from the 14th century AD up until the early 19th century AD. However, the questions regarding the inventory of the initially cultivated crops remains open, as does the query on the timing of the introduction of the sweet potato and eventually, of other South American cultivars. Other topics that remain open are related to possible shifts in time of plant cultivation and consumption.

The aim of this paper is to contribute to the reconstruction of subsistence practices of the Rapa Nui society during the settlement, particularly addressing the question of the initial introduced plant inventory, through the analysis of starch remains recovered from obsidian tools from the earliest dated site in Rapa Nui.

### The Anakena site

**Fig 1:**
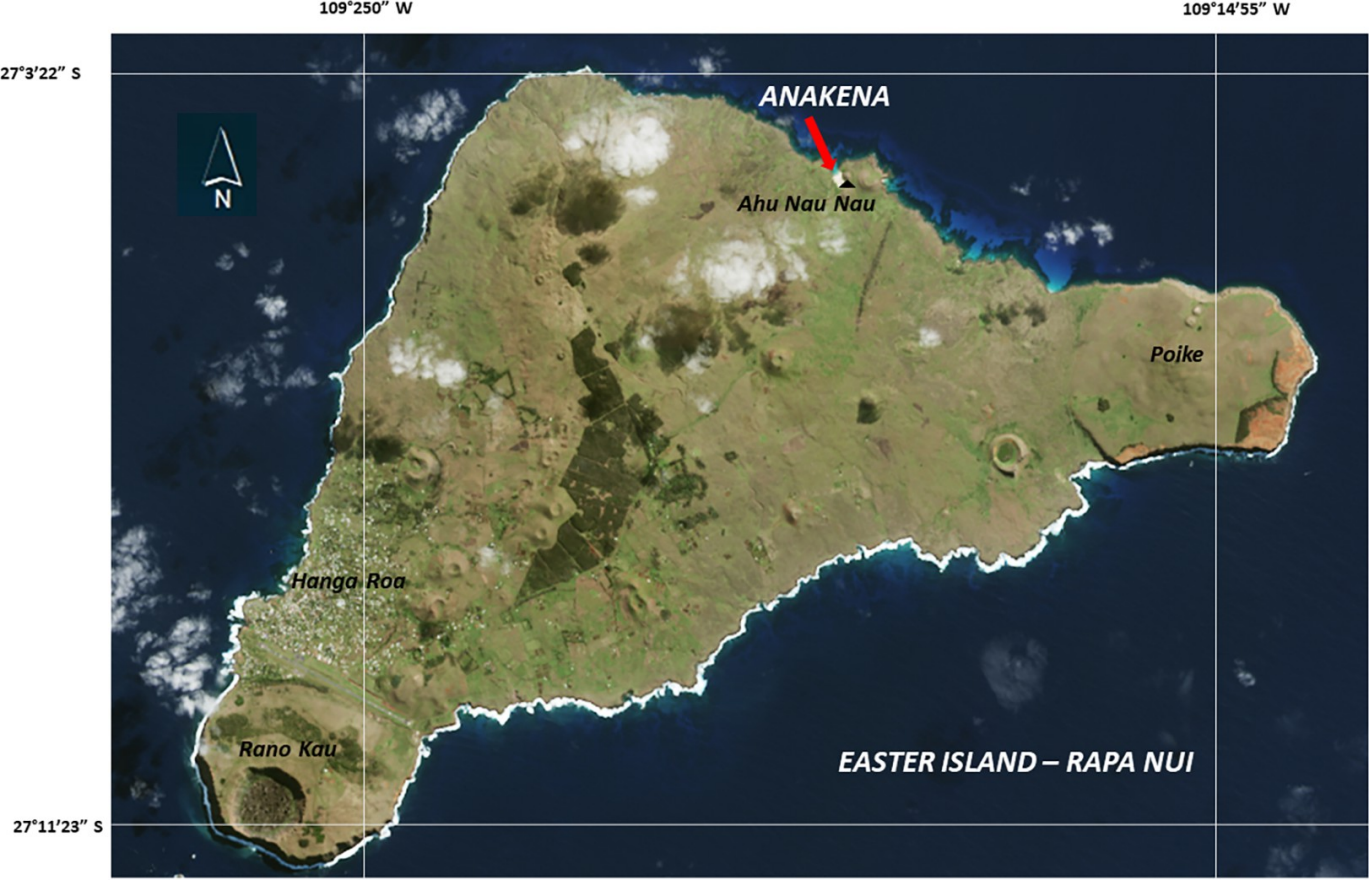
Map of Easter Island (Rapa Nui) showing the location of Anakena and the archaeological site. (base map adapted from https://eoimages.gsfc.nasa.gov/images/imagerecords/90000/90027/easterisland_oli_2015210_lrg.jpg).

**Fig 2:**
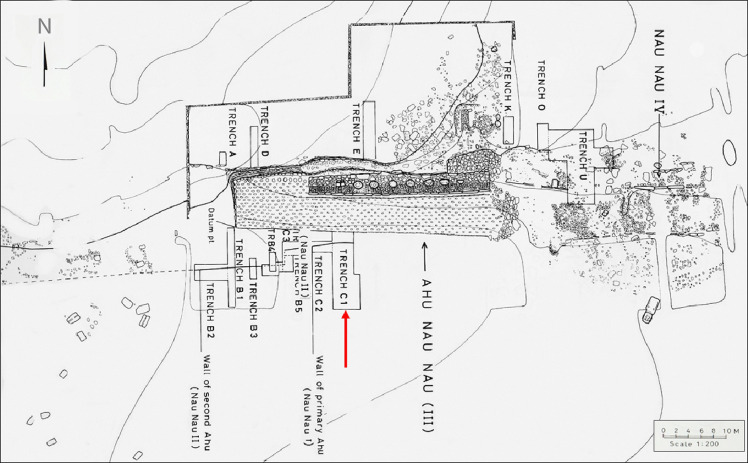
Plan view with location of excavated trenches in relation to the Ahu Nau Nau III structure. Red arrow indicates Trench C1 in front of the paved ramp of the main Ahu (Extract of map reprinted from Skjølsvold, 1994 under a CC BY license, with permission from Kon Tiki Museum).

Much of the archaeological research on Rapa Nui has focused on the study of the monumental ceremonial complexes known as Ahu [57]. One of these, Ahu Nau Nau, located on the north coast of the island, and facing Anakena Bay (**Fig 1**) was first investigated in 1934 by Henry Lavachery of the Franco-Belgian scientific mission, and then during 1955–1956 by the Norwegian expedition under the direction of Thor Heyerdahl. In 1978, extensive excavations and the restoration of Ahu Nau Nau were carried out by Sergio Rapu. Subsequently, the site was investigated between 1986 and 1988 by a team from the Kon Tiki Museum in Oslo, under the direction of Arne Skjølsvold, who was one of the members of the pioneering Norwegian archaeological expedition. The Norwegian team excavated several trenches at Ahu Nau Nau, with the main aim of documenting the early phases of the site’s architecture and to date the structure and deposits under the Ahu. In addition, this team also excavated other trenches in the vicinity of Ahu Nau Nau to study its prehistoric context [29, 57–59]. Skjølsvold [58] defined three main occupational phases of the Ahu: Ahu Nau Nau I, Ahu Nau Nau II, and Ahu Nau Nau III. Next to this platform is another platform, Nau Nau IV, which was not part of our study. The first and earliest structure of the site consists of an elevated platform without statues and a partially paved plaza area on the inland side. Cultural remains were found on top of the bedrock, under the earliest dated Ahu structure. The samples investigated in this paper derived from this cultural layer in Trench C1 (**Fig 2**). The bone remains from this same early cultural layer have been discussed elsewhere [29].

### The obsidian artifacts

A great diversity of artifacts was found in the different contexts of the various phases and particularly, the early cultural layer under the Ahu Nau Nau [58]. Radiocarbon-dated samples of rat bones and carbonized nutshells from the various contexts placed Ahu Nau Nau phase I to c. 1300 AD or a little later; construction of the phase II Ahu was placed between c. 1350–1400 AD and finally, the restored Nau Nau III phase, at around 1400 AD or a little afterwards [6, 31, 60]. A few stemmed obsidian tools known as *mata’a* were found in the most recent sand layer; basalt adzes and axes were mostly found in relationship to phases II and III [58]. Numerous obsidian tools, such as scrapers, knives, and drills were recovered mostly from under the ahu in the early cultural layer [29]. In addition, bones from several bird species, the Polynesian rat (*Rattus exulans*), and various fish species, were also found in this early layer. Martinsson-Wallin & Crockford [29] analysed the fishbones from the early cultural layer and found that pelagic fish were more abundant in the early layer and near shore fishing at later settlement stages. This is in correspondence to fishing strategies seen in other early East Polynesian sites [61]. Carbonized human bones were also found [58]. Published ^14^C dates from several ahu across the island, including Ahu Nau Nau, suggest that initial construction of these complexes likely occurred around 1250–1400 AD, but possibly as early as 1100–1200 AD. DiNapoli et al. [5], in a more recent analysis of select ^14^C samples using refined Bayesian estimates, challenge these dates, suggesting that the onset of colonization and of ahu construction occurred at a later date. Using the Bayesian model, the authors propose a colonization period between 1150–1280 cal AD and estimate the construction of the platform of Ahu Nau Nau between 1410–1450 cal AD [5], which is about 145–285 years after the initial colonization.

Anakena is one of the few sites on the island with deep and well-stratified cultural deposits, with evidence of extensive use [29, 59]. The numerous investigations that have been carried out in Anakena have allowed to date and characterize the different occupational phases, making this the earliest dated settlement on the island. The availability of the obsidian tool collection from the bottom stratum of this site offered an ideal archeological context to answer the question of the initial plant inventory.

## Material and methods

### The obsidian tool collection

**Fig 3:**
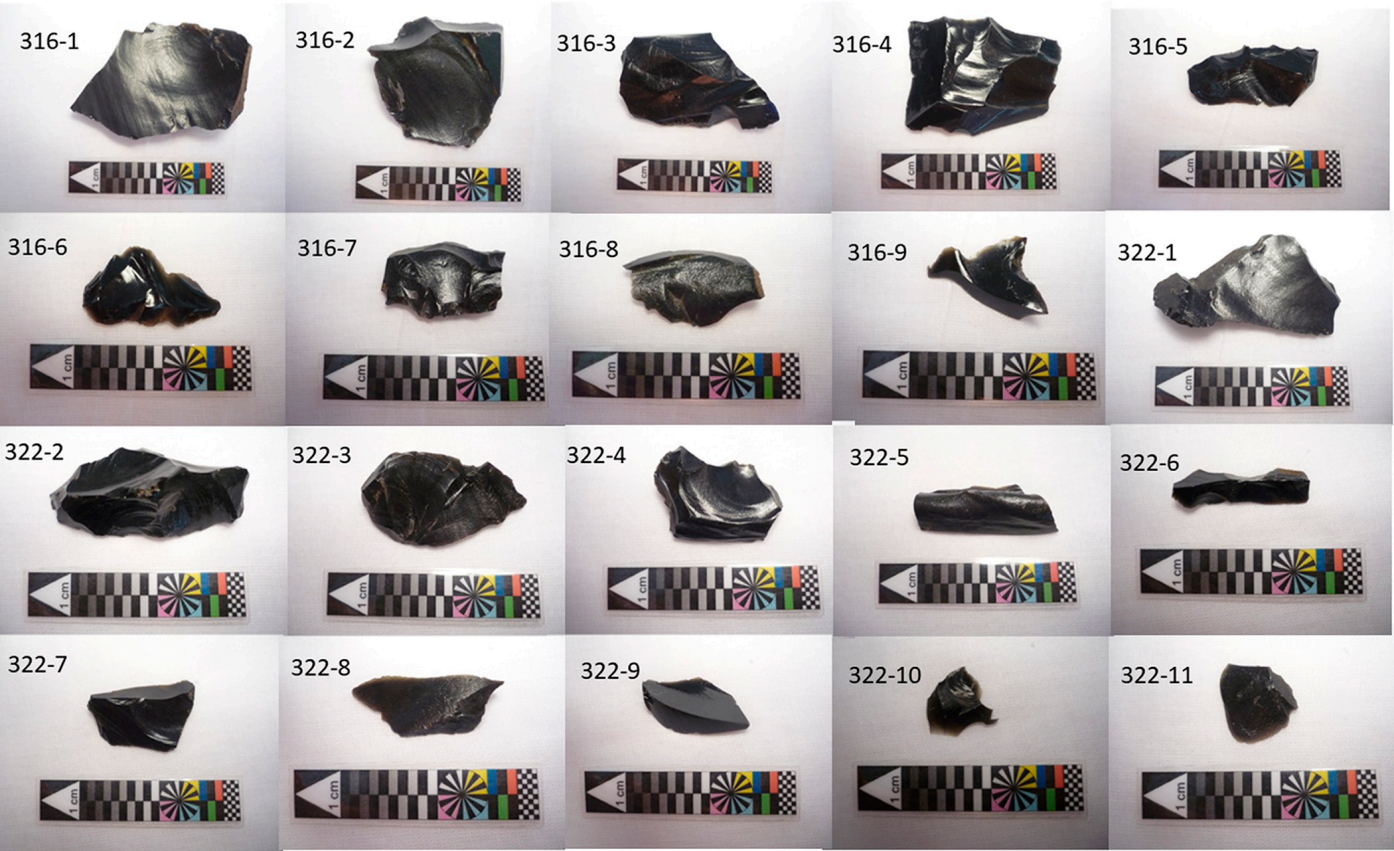
Artifacts sampled for this study.

**Fig 4:**
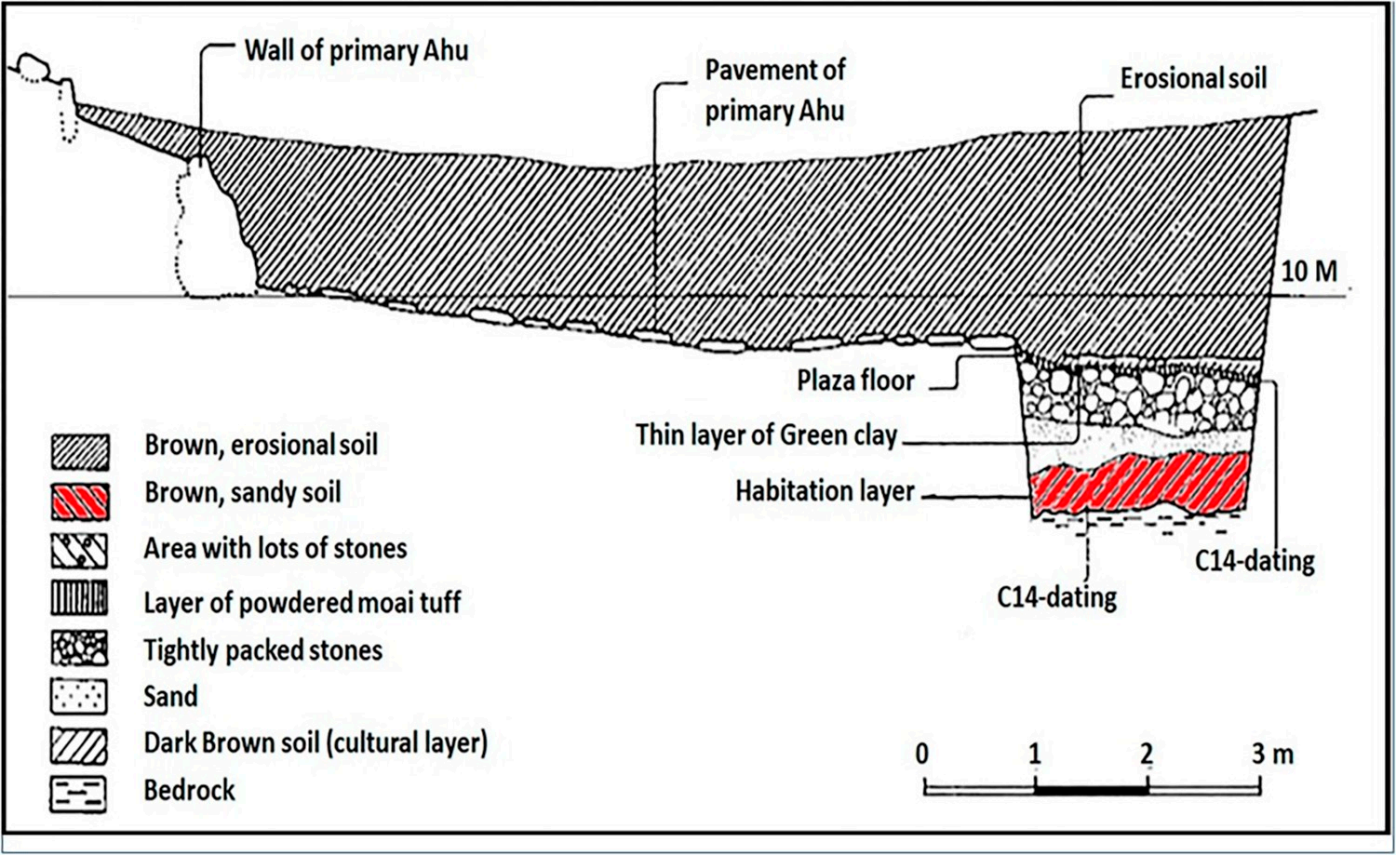
Longitudinal cross section of the profile of Trench C1. Artifacts were recovered from the layer marked in red. (Extract of origal figure in Skjølsvold, 1994 reprinted under a CC BY license, with permission from the Kon Tiki Museum).

Starch residues were analyzed from a collection of 20 obsidian artifacts excavated under the leadership of A. Skjølsvold between 1986 and 1988, and currently housed as part of the Heyerdahl collection at the Sebastian Englert Anthropological Museum (MAPSE) of Easter Island. These are sharp-edged flakes ranging in size from 16 x 15 x 2 mm to 54 x 38 x 11 mm **(Fig 3**). The artifacts were from two levels of the basal stratum of Trench C1 (**Fig 4**), located on the inland side (south side), under the plaza of Ahu Nau Nau I (**Table 1**). This trench has seven layers and extends about six meters from the ahu towards the interior of the island (**Fig 4**). The bottom layer is a c. 40–50 centimeters thick darkbrown cultural layer that sits on the bedrock. This layer was originally dated dated using traditional methods on unidentified charcoal by Skjølsvold [58]. These samples gave dates of 900±120 BP and 1170±140 BP, but due to their broad ranges they were later discarded. Later a new sample using a rat bone (*Rattus exulans*) from the bottom layer was dated by Martinsson-Wallin & Crockford [29] giving a date of 1015±65 BP (AD 890–920 at 2 SD). Additional samples from the same context were dated by Wallin et al. [31]. One sample was from the femur of an adult Polynesian rat (*Rattus exulans*), was dated to 915±65 BP (1040–1247 AD 2SD). However, carbonized endocarps of the Rapa Nui palm (*Paschalococos disperta*) from what appears to be the same level, place the occupation at 565 ±35 BP (1326–1448 AD) [29, 31]. However, Wallin et al. [31] discuss that the movement of large boulders or stones during constructing of the first ahu probably affected the bottom cultural layer. The construction phase of the first ahu above the earliest cultural layer was dated by two rat bones to 710±75 BP (AD 1210–1420 2 SD) and 610±50 BP (1300–1439 AD) and by one unidentified charcoal sample to 710±70 BP (AD 1240–1410 2 SD). The use phase of the earliest ahu was dated with a nutshell to 535±35 BP (AD 1400–1452 2 SD) and a rat bone to 640±65 BP (AD 1285–1433 2 SD). Additionally coral file sample from the habitation layer sitting on the bedrock provided dates between 956–1020 BP [31]. Considering the results from the recent dates of the bottom layer and the earliest ahu it is suggested that the early layer from this site, dates somewhere between 1000–650 BP (AD 1000–1300).

**Table 1:** List of artefacts sampled for this study.

Accession number	Museum (MAPSE) code	Collection	Original accession numbers*	Excavation unit	Artifact type	size (mm.)
0316–1	17-03-0316	03 Heyerdahl	A515-A516-A517	C1 (*bottom layer*)	flake	55.93 x 36.19 x 10.75
0316–2	17-03-0316	03 Heyerdahl	A515-A516-A518	C1 (*bottom layer*)	flake	49.63 x 43.43 x 9.27
0316–3	17-03-0316	03 Heyerdahl	A515-A516-A519	C1 (*bottom layer*)	flake	54.49 x 38.43 x 11.45
0316–4	17-03-0316	03 Heyerdahl	A515-A516-A520	C1 (*bottom layer*)	flake	47.44 x 34.14 x 11.02
0316–5	17-03-0316	03 Heyerdahl	A515-A516-A521	C1 (*bottom layer*)	flake	39.08 x 18.68 x 4.57
0316–6	17-03-0316	03 Heyerdahl	A515-A516-A522	C1 (*bottom layer*)	flake	32.37 x 19.35 x 3.85
0316–7	17-03-0316	03 Heyerdahl	A515-A516-A523	C1 (*bottom layer*)	flake	27.49 x 20.11 x 5.50
0316–8	17-03-0316	03 Heyerdahl	A515-A516-A524	C1 (*bottom layer*)	flake	31.93 x 18.26 x 3.86
0316–9	17-03-0316	03 Heyerdahl	A515-A516-A525	C1 (*bottom layer*)	flake	29.42 x 18.71 x 6.43
0322–1	17-03-0322	03 Heyerdahl	A515-A516-A517	C1 (*bottom layer*)	flake	52.04 x 25.52 x 7.91
0322–2	17-03-0322	03 Heyerdahl	A515-A516-A518	C1 (*bottom layer*)	flake	46.65 x 20.97 x 18.32
0322–3	17-03-0322	03 Heyerdahl	A515-A516-A519	C1 (*bottom layer*)	flake	40.31 x 22.02 x 7.21
0322–4	17-03-0322	03 Heyerdahl	A515-A516-A520	C1 (*bottom layer*)	flake	31.91 x 23.50 x 7.22
0322–5	17-03-0322	03 Heyerdahl	A515-A516-A521	C1 (*bottom layer*)	flake	37.07 x 13.01 x 10.18
0322–6	17-03-0322	03 Heyerdahl	A515-A516-A522	C1 (*bottom layer*)	flake	31.46 x 10.09 x 5.44
0322–7	17-03-0322	03 Heyerdahl	A515-A516-A523	C1 (*bottom layer*)	flake	28.41 x 18.13 x 4.94
0322–8	17-03-0322	03 Heyerdahl	A515-A516-A524	C1 (*bottom layer*)	flake	31.92 x 12.52 x 5.45
0322–9	17-03-0322	03 Heyerdahl	A515-A516-A525	C1 (*bottom layer*)	flake	27.06 14.46 x 6.46
0322–10	17-03-0322	03 Heyerdahl	A515-A516-A526	C1 (*bottom layer*)	flake	16.89 x 15.65 x 2.68
0322–11	17-03-0322	03 Heyerdahl	A515-A516-A527	C1 (*bottom layer*)	flake	19.14 x 17.12 x 4.00

The obsidian artifacts were chosen because they belong to an early domestic context and were potentially usable for processing plant resources. After excavation, the tools were bagged in plastic bags in the field and shipped to the Fonck Museum in Viña del Mar in mainland Chile, where basic morphometric analysis was performed. This was carried out by the team members at the archaeological laboratory of the center. Once analyzed, the obsidian collection was rebagged in plastic bags and returned to the island and stored in plastic boxes since the mid-1990’s in the museum storeroom until the current study.

### Sampling protocol of archaeological starch grains

The issue of contamination from modern airborne starch has been raised by several authors [62, 63]. Laurence et al. [64] and Sikoparija et al. [65], refer to the possibility that airborne starch particles, which are part of a wide spectrum of suspended biological materials may contaminate samples. The issue is relevant for our samples that were analyzed in the laboratory of the Fonck Museum almost 30 years ago. Starch granules under certain conditions may settle on surfaces and glass slides if these are left open. However, according to these and other authors [66, 67] abundance of these vary according to climate conditions, particularly rainfall and wind directions and thunderstorms. Sikoparija et al. [65] additionaly indicate that most events of “starch rain are associated to specific anthropogenic activites. In addition, Mercader et al. [68] show that starch grains derived from contemporary plants may be present in topsoils.

The obsidian collection was stored in the museum in lidded plastic boxes, which minimizes possible contamination from airborne starch during this period. Since our obsidian flakes selected for starch extraction were excavated many years previous to the present study, it was not possible to obtain soil samples for comparison with surface soil material or soil from the excavation context. However, since the tools are from the bottom layer of a trench 3,5 m below modern ground surface (and under a sand dune), contamination from modern vegetation is unlikely.

Sampling of starch residues was undertaken at the conservation laboratory of the museum. For this study, flakes were rinsed with distilled water to eliminate any surface sediments that could have accumulated during storage in the Museum and that could carry possible contaminants [see 69, 70]. Flakes were observed under a binocular microscope for evidence of starch remains and were scraped directly onto a microscope slide using a single use disposable plastic toothpick. Slides and coverslips were previously cleaned with distilled water and ethyl alcohol to avoid sample contamination. This cleaning protocol was also applied to all instruments used during the preparation of the samples [71–73]. Flakes that did not show evident starch residues under the microscope were still sampled. In this case, flake edges were scraped directly over a microscope slide. As mentioned by Babot [72], it is important to sample surfaces even if obvious starch residues are not visible even under the binocular microscope, since starch grains get lodged in the rock fissures and striations or may be too small to be seen under small magnification. A drop of glycerin was added to the sample, which was homogenized with the aid of a disposable agitator. Slides were then covered with a 25 x 25 mm coverslip and sealed with nail polish. As indicated, all possible safeguards were taken to avoid contamination with contemporary starches. This slide collection was then taken to Santiago for analysis in our laboratory at the Facultad de Ciencias Básicas, Universidad Metropolitana de Ciencias de la Educación.

### The starch reference collection

The starch reference collection was prepared from 13 plant species (**Table 2**) in a separate laboratory from where the archaeological samples were stored. The focus of this study was on starch from i) traditional Polynesian economic plants, ii) South American crops, and iii) one Eurasian crop species introduced to the Pacific and the Americas after European contact as an external control species. Plants that currently do not grow on Easter Island, such as *Inocarpus fagifer* or *Spondias dulcis*, as well as some economic species from South America that could have been transported along with sweet potato as part of the crops taken by Polynesians on return voyages from South America, were also included, in particular since some of these species were seen growing on the island in 1770 [74]. The starch reference collection used in this research is composed of the following food and utilitarian species: i) Polynesian canoe plants, *Artocarpus altilis*, *Colocasia esculenta*, *Curcuma longa*, *Dioscorea alata*, *Inocarpus fagifer*, *Manihot esculenta*, *Musa* sp., *Spondias dulcis*, and *Zingiber officinale*, ii) plants of South American origin, *Canna* sp., *Ipomoea batatas*, and *Xanthosoma* sp., and iii) *‎Triticum aestivum*, since this species does not belong to the plant groups (Polynesian or South American) described above. *Triticum* was included as a control species since it is frequently used in Chile and its presence would indicate modern contamination of the archaeological samples. Of the other plants, *Sponidas dulcis*, *Inocarpus fagifer*, and *Triticum* sp. are currently not being cultivated or planted on the island. Specimens were collected by AS in Tonga, Tahiti, and Easter Island. Samples from Santiago were collected by PB and AS. The sample of *Dioscorea alata* from the Solomon Islands was collected by Anne di Piazza (CREDO, France). Vouchers were housed at the Biology Laboratory of Universidad Metropolitana de Ciencias de la Educación, Santiago, Chile.

**Table 2:** List of 13 sampled plant species, plant parts, geographic origin, number of organs sampled per plant, and number of starch grains considered for the reference collection of this study.

Family	Species	Common name	Organ	Number of organs	Provenance	Number of starch grains
Anacardiaceae	*Spondias dulcis*	Tahitian apple	Fruit	1	Tahiti	100
Araceae	*Colocasia esculenta*	*Taro*	Corm	2	Rapa Nui	200
Araceae	*Xanthosoma sp*.	Malanga, *Taro vaihi vaihi*	Corm	1	Rapa Nui	100
Cannaceae	*Canna* sp.	Achira	Rhizome	1	Santiago	100
Convovulaceae	*Ipomoea batatas*	Sweet potato	Tuber	1	Rapa Nui	100
Convovulaceae	*Ipomoea batatas*	Sweet potato	Tuber	1	Santiago	100
Dioscoreaceae	*Dioscorea alata*	Yam	Tuber	1	Solomon Isl.	100
Euforbiaceae	*Manihot esculenta*	Manioc	Tuber	1	Santiago	100
Fabaceae	*Inocarpus fagifer*	Tahitian chestnut/*mape*	Seed	1	Tahiti	100
Moraceae	*Artocarpus altilis*	Breadfruit	Fruit	1	Tahiti	100
Moraceae	*Artocarpus altilis*	Breadfruit	Fruit	1	Tonga	100
Musaseae	*Musa* sp.	Banana	Fruit	1	Tahiti	100
Zingiberaceae	*Curcuma longa*	Turmeric	Rhizome	1	Tahiti	100
Zingiberaceae	*Zingiber officinale*	Ginger	Rhizome	1	Santiago	100
Poaceae	*Triticum aestivum*	Wheat	Seed	1	Santiago	100

All specimens were mounted in duplicate and for each mounting, 50 starch grains were analyzed. The sampling methodology was based on the protocols proposed by Babot [71] and Scott Cummings [73]. In the case of fresh specimens, each organ was cut with a sterile razor blade to expose its interior. In some cases, the starch grains were obtained by grinding on a mortar and adding drops of distilled water when needed. With a sterile syringe, some drops were added over one drop of immersion oil above the slide. In other cases, a direct smear of the organ on the glass slide was obtained. Dry samples were hydrated in distilled water for 30 minutes, prior to the sample extraction protocol described above. Immersion oil 300 (Thomas Scientific) was added to the sample and homogenized with the aid of a disposable agitator. Slides were then covered with a 25 x 25 mm coverslip and sealed with nail polish. Finally, each slide was labeled with a unique number consisting of the acronym SGO-AL, the number assigned to each mounted copy, and a letter "a" or "b" to differentiate the duplicate mountings.

### Data collection

All samples (reference collection and archaeological samples) were observed with a trinocular transmitting bright field microscope (Leitz, Dialux 22) fitted with a polarizing filter, using 40x magnification. A Moticam 10 MP digital camera and the Micrometrics software (Motic Images plus 3.0) were used for image capturing, archiving, and measuring. Each starch grain received an individual accession number.

Archaeological starch grains were distinguished by the prefix “Ala”. The mounted slides with the archaeological starch grains were observed under the microscope traversing the slide from left to right and from top to bottom, in each ocular field. The four inner edges of each slide were also scanned. Each archaeological grain was photographed under visible and polarized light, measuring the same parameters as for grains in the reference collection.

All starch grains of the reference collection were individually measured, and quantitative and qualitative attributes were registered for each grain. Quantitative variables used follow Torrence et al. [75] and correspond to the maximum and minimum length of the starch grain, total area, total perimeter, maximum and minimum distance between the hilum, and the edge of the grain. Briefly, four measurements for maximum and minimum length were made using the Motic Image plus 3.0 software line tool, considering the symmetry axis of each grain. Variables were measured as depicted in **S1 Fig.** With the aim of adding new metric parameters that would allow improving the possibilities of accurately distinguishing between starch grains, we established three indexes based on quantitative measurements. The first index was **compactness** (*cI*), based on the Gravelius index [76] used in geomorphology to describe landform shapes. The *cI* index was calculated with the following formula: (0.282*P)/(√A), where P is the perimeter and A is the area of the starch grain. Obtained values were divided in three qualitative categories: circular (= 1), elongated (1.01–1.25), and irregular (>1.26). Additionally, we established an **elongation** index (*eI*), and a **centricity** index (*ceI*). The compactness and elongation indexes are related to the morphology of the starch grain, while the centricity index relates to the hilum. The result provides a simplified version of of the starch grain shapes and the hilum, and are calculated using the following formulas:

The *el* index is calculated by dividing the maximum length by the minimum length of the starch grain. Obtained values were grouped into four ranges: not elongated (= 1), slightly elongated (1.01–2), moderately elongated (2.01–3.0), and highly elongated (>3.01). The *ceI* index (*d max*/(*d* max + *d* min)) allows determining the degree of deviation of the hilum with respect to the center of the starch grain. The degree of centricity was classified as centric (0.5–0.6), eccentric (0.61–0.70), or hyperexcentric (0.71–1.0).

Seven qualitative variables were also considered, based on descriptive variables and values, as defined by Torrence et al. [75], Allen and Ussher [21], and Pagan [77]: the two- dimensional shape of the starch grain, visibility and the location of the hilum, type of hilum (open or closed), fissure in the hilum, shape of the fissure in the hilum, pressure facets, lamellae, and style of the extinction cross. In terms of shape, the most common two-dimensional shapes were considered: circular, oval, oblong, oblanceolate, polyhedral, polymorphic, or bell-shaped. The categorization of the 2-dimensional shapes of starch grains is shown in **S2 Fig**. The shape of the arms (style) of the extinction cross is one of the key variables for the identification of starch grains. These were recorded as straight, curved, or wavy (**S3 Fig**). For the hilum, we considered three types: non-visible, closed, or open. Since some starch grains have fissures that originate in the hilum, we recorded their shapes in the following categories: circular, simple, v-shaped, y-shaped, elongated, hat/square-shaped, and absent (**S3 Fig)**. Starch grain facets were registered as absent, flat, concave, flat multi-faceted, concave multifaceted, and mixed multi-faceted. Lamellae were considered as present or absent. However, since archaeological grains did not present this feature, this variable was not considered in the final analysis. For a general description of the morphotypes associated with each species in the reference collection, see **S1 Text**. All data were transferred to a spreadsheet and qualitative variables were categorized for the statistical analysis. The joint analysis of quantitative and qualitative variables in the same matrix is justified on the basis that these variables can be related, and therefore, this type of analysis provides a more complete understanding of the phenomena under study. From a holistic point of view, this approach allows capturing both numerical variation and category or group differences. This is especially relevant when trying to gain a fuller understanding of a phenomenon or research problem, as it considers the complexity of reality, and the observer can obtain a more complete view of the relationships between variables. By considering quantitative and qualitative variables together, greater analytical power can be obtained to detect underlying relationships and patterns. Information obtained from data considering combined quantitative and qualitative variables helps to avoid simplistic or biased conclusions. By jointly analyzing quantitative and qualitative variables, complex interactions between different dimensions or aspects of a phenomenon can be captured.

### Exploratory data analysis

To maximize precision in the identification of the archaeological grains, an exploratory analysis of the data [78] was carried out using the R program [79]. This exploratory analysis permits a quick understanding of the nature of the data in the reference collection, considering summary statistics and data visualization. Summary statistics provide values that explain data properties, such as central tendency and data dispersion, thus indicating a measure of data variability. For data visualization, and to find patterns or groups (clusters) within the reference collection, we carried out an unsupervised analysis. The unsupervised analysis ignores the response variable that indicates to which group each observation belongs; that is, the program works with the complete data set and allows to define to which group each observation belongs [80]. As part of this unsupervised analysis, and to determine which variables were more informative, a principal component analysis (PCA) was carried out with the data matrix of the reference collection. In addition, the following classification algorithms were implemented: Vector support machine with Kernel (Support Vector Machine SVM) [81], and classifier with Random Forest [82].

Because the scale of the variables affects the clustering results, the reference and archaeological data matrixes were scaled to give equal weight to all variables used and to ensure that when constructing the classification algorithms, the classification is based only on similarity [83]. To validate the classification algorithms, the reference collection database was subsequently used to obtain a training set (75%) and a validation set (25%) [80].

### Identification of archaeological starch grains

Based on the results obtained from the unsupervised analysis (described in the results section), a Linear Discriminant Analysis was performed as a supervised classification method [80], hereby obtaining a data subset of the reference collection matrix that was then used to identify archaeological starch grains. Finally, the identification of the archaeological starch grains was carried out by means of a Linear Discriminant Analysis. This supervised analysis was performed with the combination of the data subset of the starches from the reference collection, combined with the data matrix of the archaeological starches.

## Results

### Exploratory analysis of the data matrix of the reference collection

The reference dataset consisted of 1598 starch grain observations from the following species: *Artocarpus altilis* (n = 200), *Canna* sp. (n = 100), *Colocasia esculenta* (n = 200), *Curcuma longa* (n = 100), *Dioscorea alata* (n = 100), *Inocarpus fagifer* (n = 100), *Ipomoea batatas* (n = 200), *Manihot esculenta* (n = 100), *Musa* sp. (n = 100), *Spondias dulcis* (n = 100), *Xanthosoma* sp. (n = 98), *Zingiber officinale* (n = 100), and wheat (*Triticum eastivum*) (n = 100). The statistical summary of the variables of the reference collection data set shows variability and scale differences between the minimum and maximum values (see **(Tables 1 to 13 in S1 File)**). Therefore, variables of the data matrix were rescaled.

All slides with the archaeological samples were viewed under polarized and visible light at 40x magnification. In total, 78 items that presented starch characteristics, such as birefringence under polarized light, were photographed. Of these 78 items, six were later discarded since after further observation they were identified as microcrystaline structures. The remaining 72 had the classic characteristics of starch granules, as described in the literature [77, 84, 85]. Of these, 26 grains were discarded from the analysis because they were either damaged, were found on the edge of the slide and under the nailpolish, or measurements of all variables could not be performed under visible light (see **S4 Fig**). Consequently, the statistical analysis included 46 archaeological grains. Quantitative measurements and categorization of the qualitative variables taken on each archaeological starch grain are shown in **(Table 14 in S1 File).**

#### Non-supervised analysis

**Fig 5:**
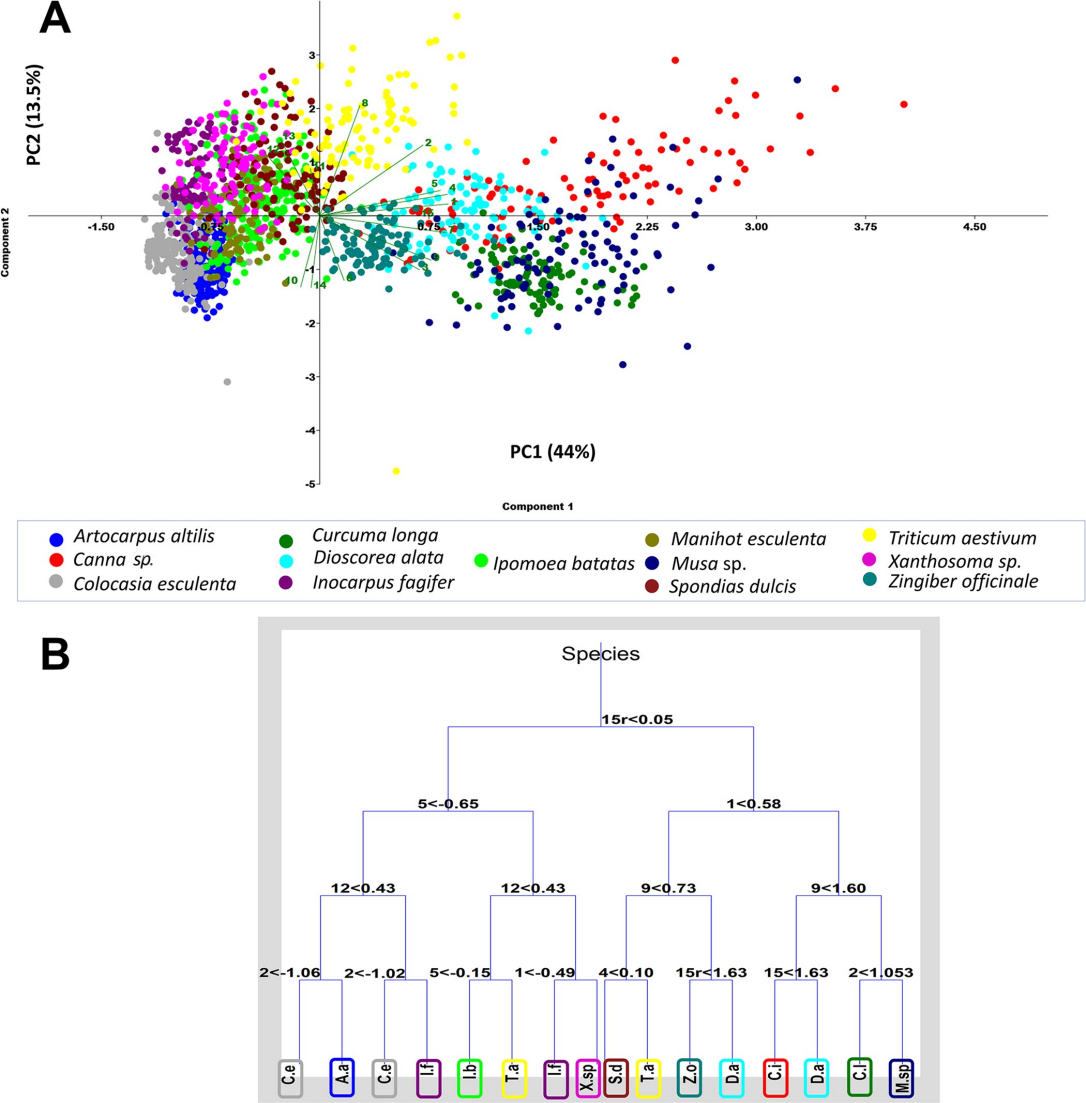
Principal Component Analysis (A) and Classification Tree (B) with the 15 variables measured in the 13 plant species of the reference collection (1598 observations). Variable codes are shown in the Biplot (Eigenvalue scale) and in the Decision tree: 1: Maximum grain length. 2: Minimum grain length. 3: Elongation index. 4: Total perimeter. 5: Total area. 6: Compactness index. 7: Maximum distance between the hilum and edge. 8: Minimum distance between the hilum and edge. 9: Centricity index. 10: 2D shape. 11: Hilum type. 12: Hilum fissure. 13: Hilum fissure shape. 14: Facets. 15: Style of extinction cross. The percentage of variance explained by components 1 and 2 are shown on the axes. The decision tree shows 31 nodes, fifteen of which are associated with a binary decision with branches to the left and right. Sixteen nodes are terminal branches.

For the initial unsupervised analysis, a PCA was carried out to condense the information provided by the 15 analyzed variables to only a few major components. The PCA also allows to determine which variables are more informative. The first two main components were plotted **(Fig 5A**), which account for 57.5% of the data variability: 44% in the case of the first component (PC1), and 13.5% for the second component (PC2). This suggests that 42.5% of the data variability is accounted for by the other 13 components. Therefore, by reducing the dimensionality, original data information is condensed to simplify the analysis; however, part of the information is also lost. The eigenvalues and percentages of variation explained by the principal components for each of the 15 variables analyzed are specified in **(Table 15 in S1 File)**.

#### Classification algorithm of unsupervised analysis

*Classification tree*. A decision tree was built with the original data set and the 15 variables. The diagram in **Fig 5B** shows that 77.53% of the starch grains could be classified correctly and that some of the binary and terminal nodes included the same species, indicating that intraspecific variability was greater than interspecific variability, hence precluding classification precision to 100% for each of the species.

**Fig 6:**
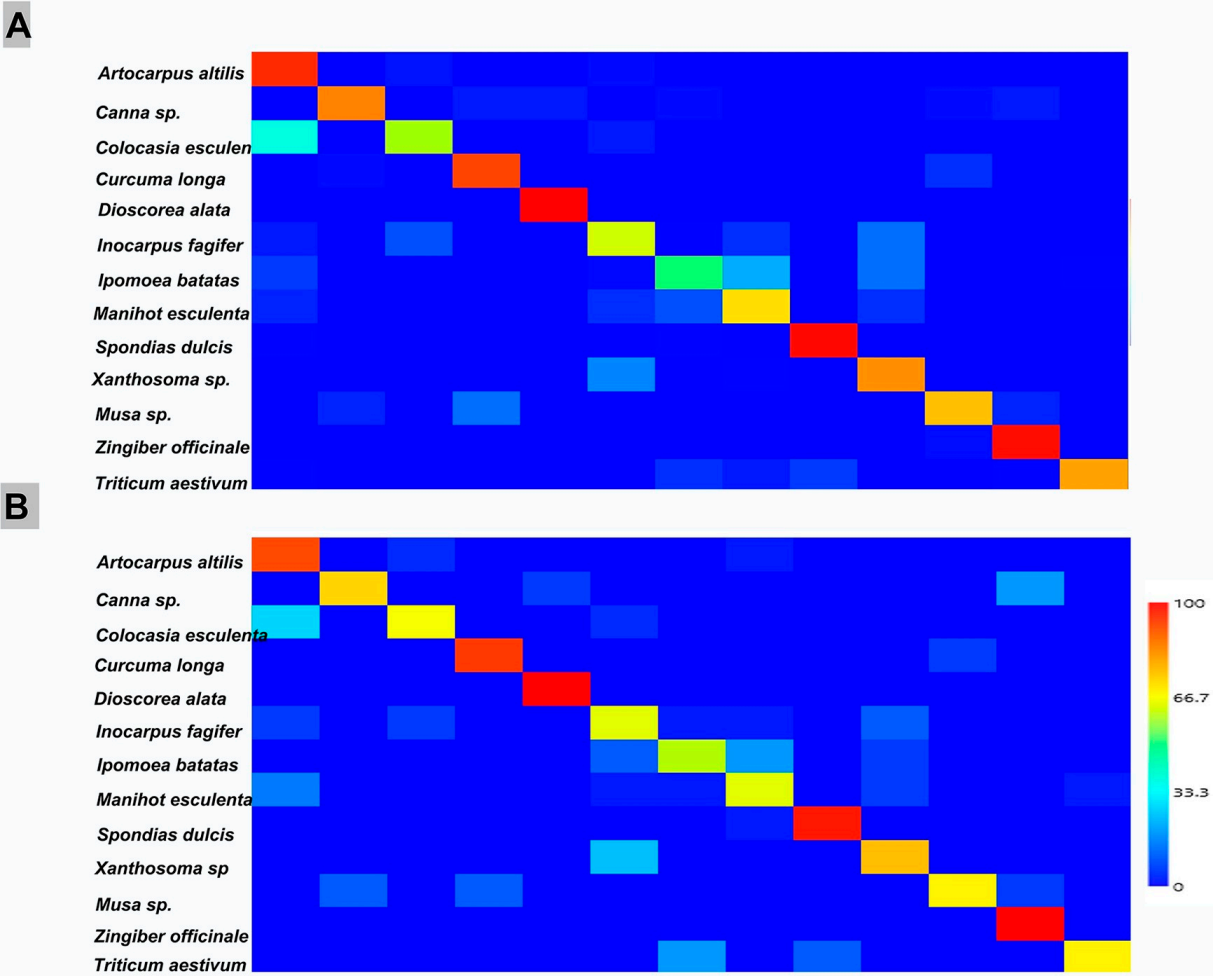
Heat map of the two confusion matrixes generated with the complete and the training data set. **A)** Complete data set; B) Training set.

To validate the quality of the classification algorithms, a 75% training set and a 25% validation set were established from the original data set of 1598 observations. The confusion matrix generated by both the Support Vector Machine (SVM) with kernel and the Random Forest classifier, did not provide a prediction accuracy of 100% for each species of starch grain. This is congruent with the great intraspecific variability observed in the data matrix with the 15 variables analyzed. **Fig 6** shows a heat map built from the two confusion matrices generated with the training set (A) and with the original data set (B). Both matrices show that the *A*. *altilis*, *S*. *dulcis*, *D*. *alata*, and *Z*. *officinale* species showed high prediction precision (> 80%, range 81–100%), while the species that presented the lowest prediction precision in the total data set were *I*. *fagifer* and *I*. *batatas* (<50%).

#### Classification algorithms of the supervised analysis for the identification of archaeological starch grains

Considering that the unsupervised analysis was unable to predict correct species allocation with 100% accuracy and that reducing the dimensionality by means of the PCA resulted in the loss of original data, we chose to perform a discriminant analysis with the total data set (1598 observations). Of these, a data subset of 1240 observations that corresponded to the number of starch grains were correctly classified. As a result, the comparative collection analysis matrix used to classify the archaeological starches was constructed on a reduced number of starch grains from each species: *A*. *altilis* (n = 187), *Canna* sp. (n = 81), *C*. *esculenta* (n = 121), *C*. *longa* (n = 94), *D*. *alata* (n = 100), *I*. *fagifer* (n = 59), *I*. *batatas* (n = 103), *M*. *esculenta* (n = 71), *Musa* sp. (n = 71), *S*. *dulcis* (n = 97), *Triticum* sp. (n = 78), *Xanthosoma* sp. (n = 80), and *Z*. *officinale* (n = 98).

**Fig 7:**
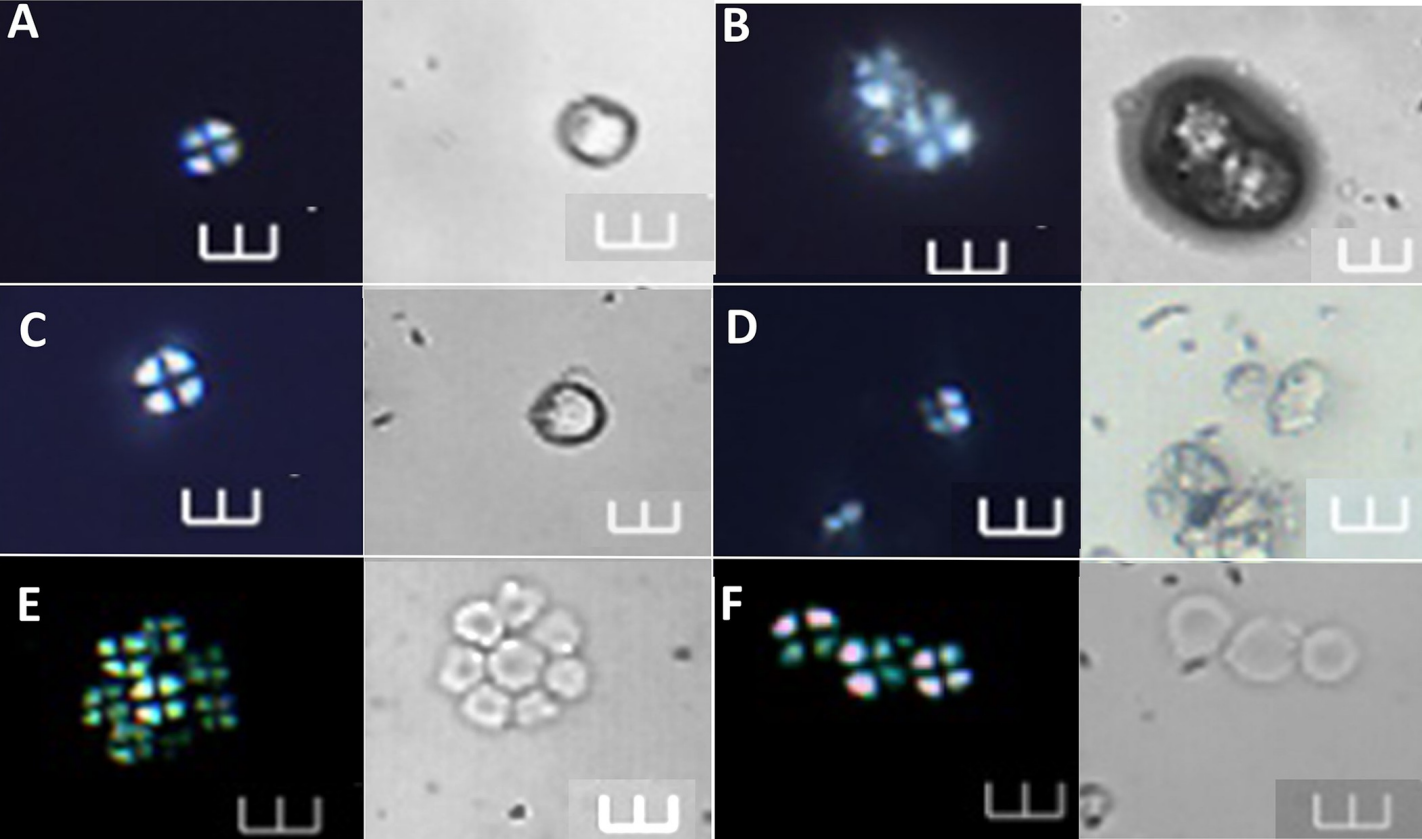
Starch grains identified at >90% confidence to *Artocarpus altilis*, shown under polarized and brightfield light. A–D Archaeological starch grains under polarized and brightfield light (Ala 43, Ala 52, Ala 56, and Ala 07, respectively). E-F Starch grains of *A*. *altilis* from the reference collection. Scale bars = 10 μm.

**Fig 8:**
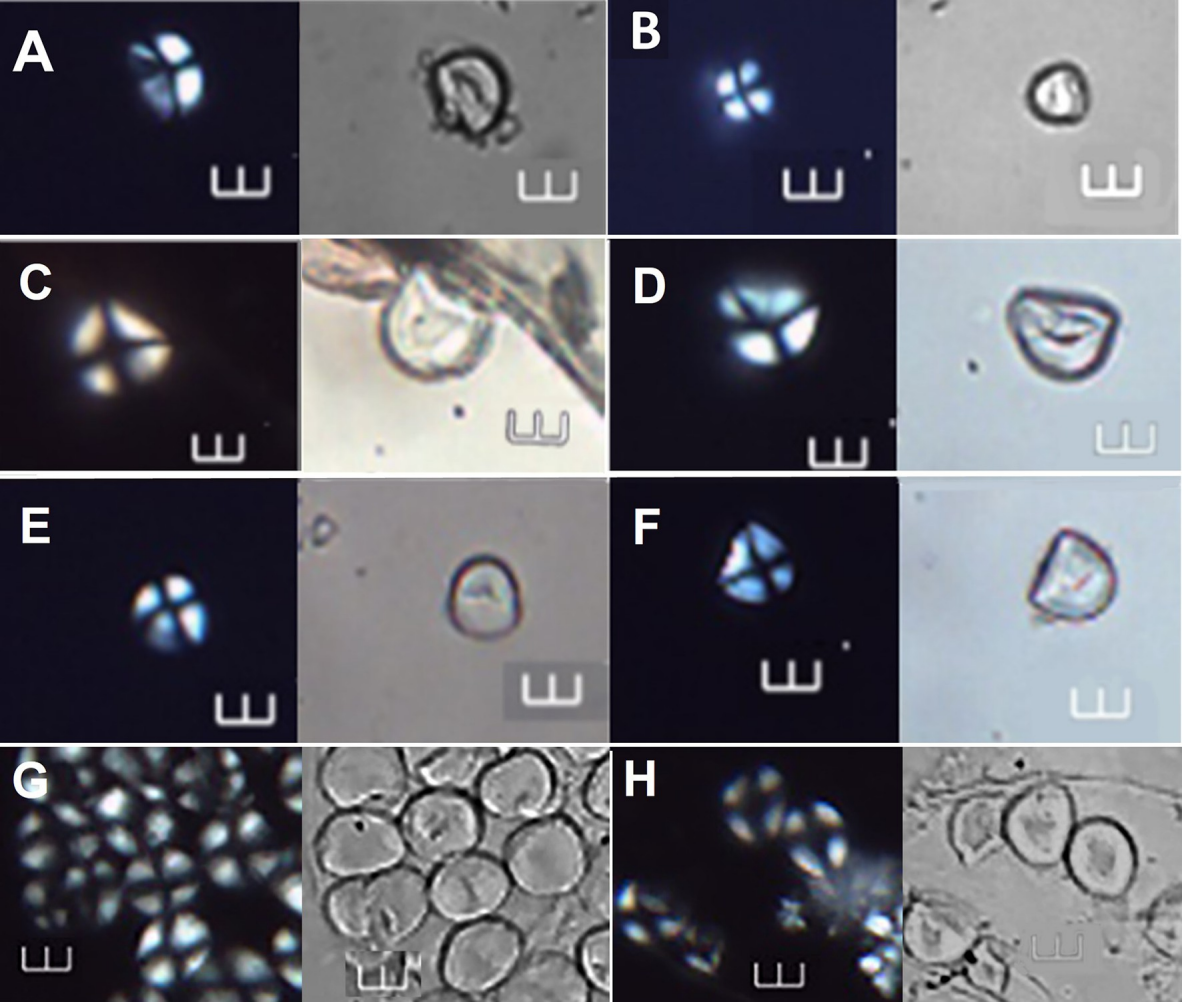
Starch grains identified at >90% confidence to *Spondias dulcis*, shown under polarized and brightfield light. A–F Archaeological starch grains under polarized and brightfield light (Ala 13, Ala 48, Ala 21, and Ala 23, respectively). G-H. Comparative starch grains of *S*. *dulcis* from the reference collection. Scale bars = 10 μm.

**Fig 9:**
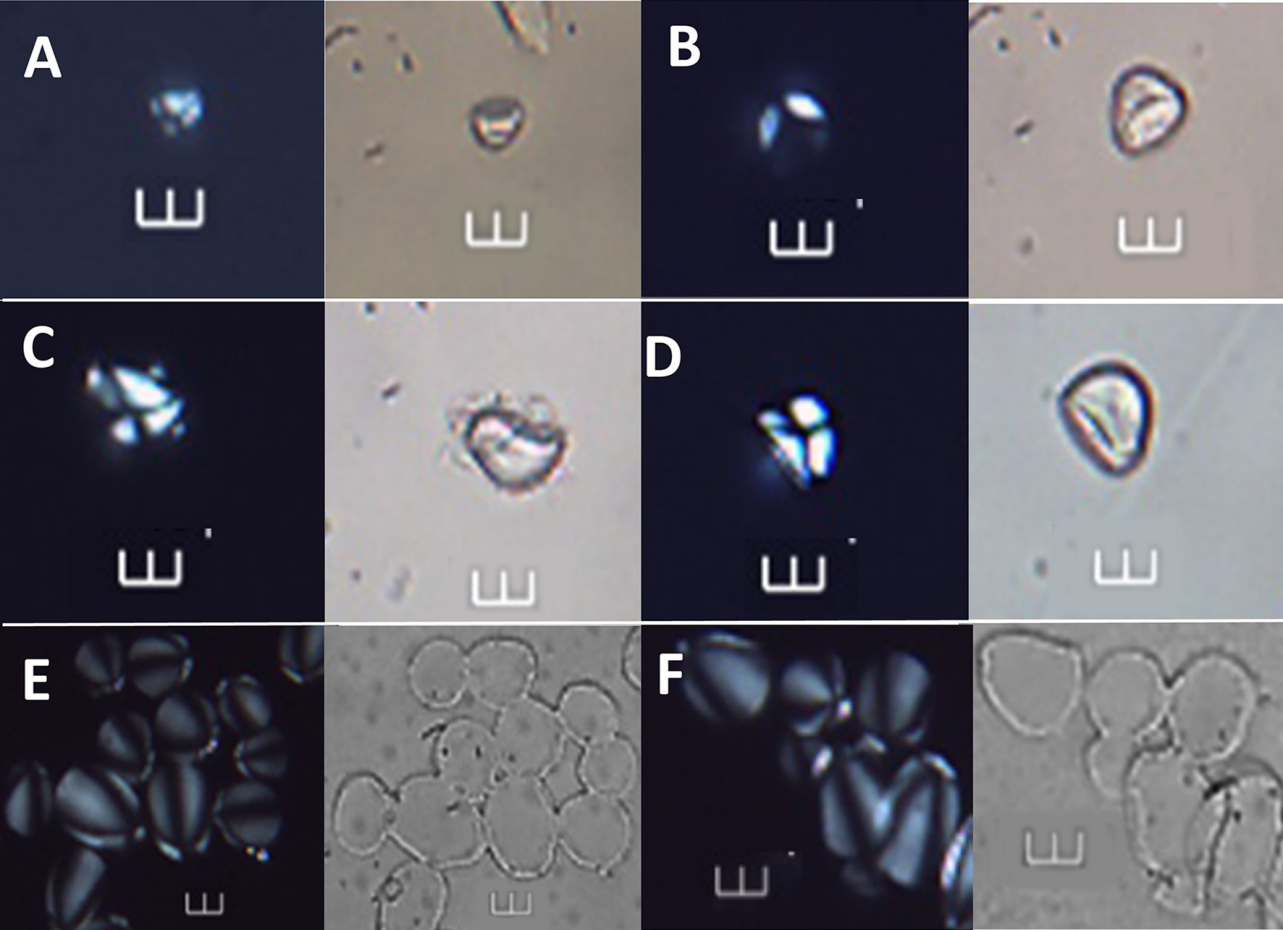
Starch grains identified at >90% confidence to *Zingiber officinale*, shown under polarized and brightfield light. A–D (Ala 14, Ala 47, Ala 46, and Ala 57, respectively). E-F Starch grains of *Z*. *officinale* from the reference collection. Scale bars = 10 μm.

**Fig 10:**
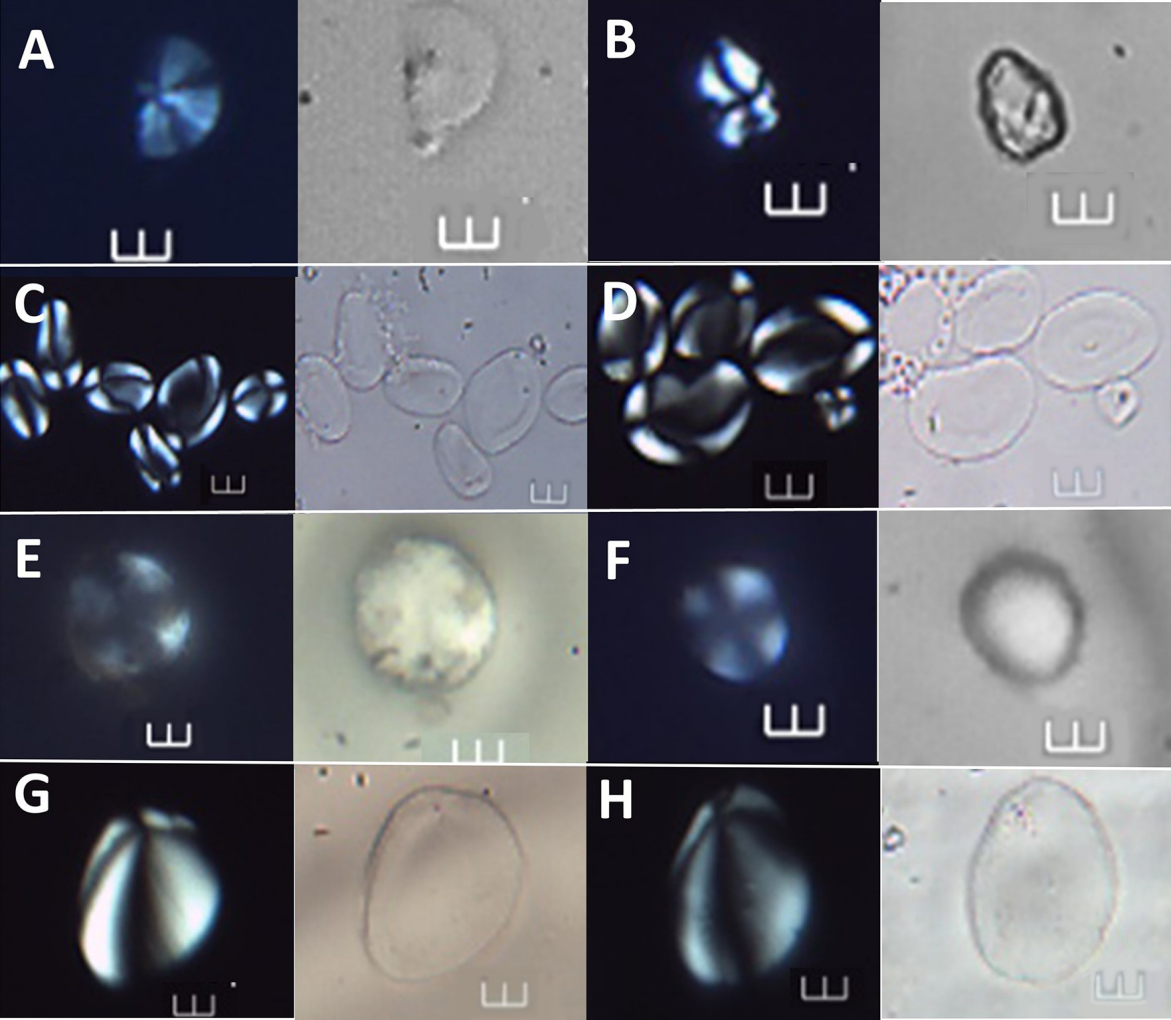
Starch grains identified at >90% confidence to *Dioscorea alata* and *Canna* sp., shown under polarized and brightfield light. A–B Archaeological starch grains (Ala 06 and Ala 75) assigned to *D*. *alata*. C—D Starch grains of the reference collection of *D*. *alata* under polarized and brightfield light. E–F Archaeological starch grains (Ala 15 and Ala 41) assigned to *Canna* sp. G—H Starch grains of *Canna* sp. from the reference collection Scale bars = 10 μm.

**Fig 11:**
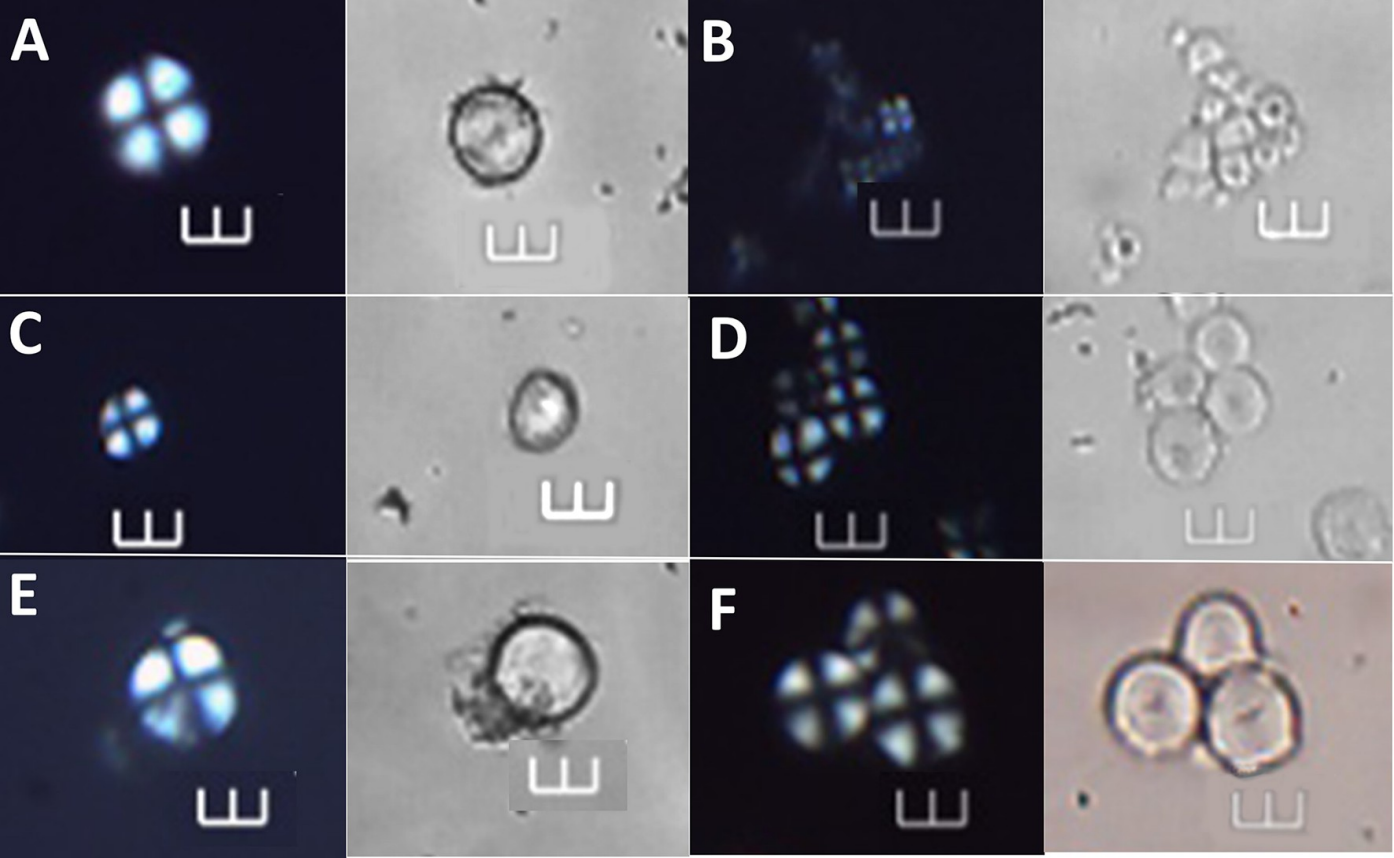
Starch grains identified at >90% confidence to *Colocasia esculenta*, *Ipomoea batatas*, *and Manihot esculenta*, shown under polarized and brightfield light. A, C and E Archaeological starch grains under polarized and brightfield light (Ala 37, Ala 24, and Ala 39) identified to the following species: *C*. *esculenta*, *I*. *batatas*, and *M*. *esculenta*, respectively. B, D and F Starch grains of the reference collection under polarized and brightfield light of *C*. *esculenta*, *I*. *batatas*, and *M*. *esculenta*, respectively. Scale bars = 10 μm.

**Fig 12:**
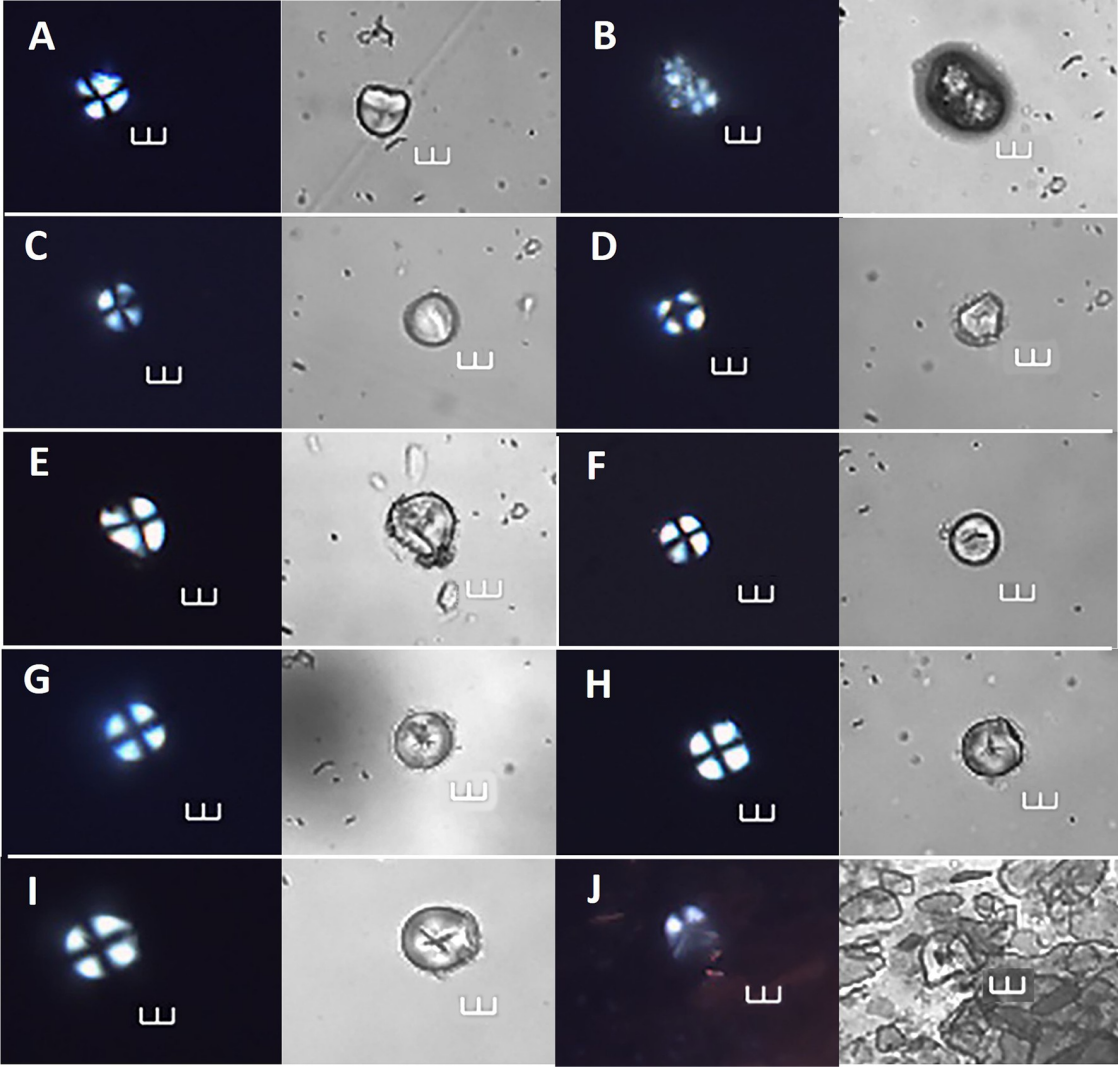
Archaeological starch grains identified with >90% probablity as most similar to other archaeological grains, under cross polarized and brightfield light. A = Ala 50, B = Ala 52 and 53, C = 55, D = Ala 60; E = Ala 61, F = Ala 65, G = Ala 71, H = Ala 72, I = Ala 74, and J = Ala 77.Visual inspection allowed assignation of grains as follows: Ala 50 to *I*. *batatas* or *M*. *esculenta;* Ala 53 to *A*. *altilis;* Ala 55 to *I*. *batatas;* Ala 60 and 61 to *I*. *fagifer;* Ala 61 and 65 to *I*. *batatas;* Ala 71, Ala 72, and Ala 74 *to M*. *escuelnta;* and Ala 77 to *Xanthosoma* sp. Scale bars = 10 μm.

**Table 3** shows the results of using the derived discriminant functions to classify the 46 starch grains from the archaeological collection. It lists the two highest scores among the classification functions for each of the starches in the archaeological collection within the reference collection. Of the 46 archaeological starches found on the obsidian artifacts, 21 were securely classified with probabilities greater than 90%, and assigned to a species in the reference collection (*A*. *altilis* = 5; *Canna* sp. = 2; *C*. *esculenta* = 1; *D*. *alata* = 2; *I*. *batatas* = 2; *M*. *esculenta* = 1; *S*. *dulcis* = 4; and Z. *officinale sp*. = 4) **(Figs 7–11)**). Eight grains were classified as closer to other archaeological grains than to starches from the reference collection (**Fig 12**). Furthermore, **five** archaeological grains were classified with probabilities between 80% and 90% (*A*. *altilis* = 1, *D*. *alata = 1*, *M*. *esculenta* = 1, and *S*. *dulcis* = 2). Finally, 12 other starch grains could not be securely classified since the classification was below p <0.80 (**Table 3**). Two of these grains were classified closest to other archaeological grains, and ten had similar characteristics to several superimposed species, such as *I*. *batatas*, *I*. *fagifer*, and *M*. *escuenta*, as shown in **Fig 5A,** or may belong to a species not included in the reference collection.

**Table 3:** Species allocation of the 46 archaeological starch grains based on results of the derived discriminant functions analysis.

Archaeological starch grain	Highest group	Highest value	Squared distance	Prob.	2nd highest group	2nd highest value	Squared distance	Prob.
Ala 0002	*Ipomoea batatas*	-0,80	32,87	0,78	*Manihot esculenta*	-2,08	35,43	0,22
Ala 0004	*Ipomoea batatas*	-3,89	26,33	0,56	Archeo	-4,15	26,86	0,43
Ala 0005	***Artocarpus altilis***	0,82	117,88	0,93	*Ipomoea batatas*	-2,08	123,67	0,05
Ala 0006	***Dioscorea alata***	18,65	146,02	1,00	*Curcuma longa*	3,15	177,01	0,00
Ala 0007	***Artocarpus altilis***	7,89	21,75	0,97	*Manihot esculenta*	4,32	28,90	0,03
Ala 0010	*Ipomoea batatas*	0,33	12,80	0,52	*Manihot esculenta*	0,23	13,00	0,47
Ala 0011	*Inocarpus fagifer*	-2,85	33,23	0,43	*Ipomoea batatas*	-3,06	33,64	0,35
Ala 0012	*Spondia dulcis*	-2,61	40,07	0,52	Archeo	-2,70	40,25	0,48
Ala 0013	***Spondia dulcis***	0,91	52,04	1,00	Archeo	-4,94	63,73	0,00
Ala 0014	***Zingiber officinale***	3,34	102,70	1,00	Archeo	-2,54	114,44	0,00
Ala 0015	***Canna* sp.**	22,32	112,31	1,00	*Musa* sp.	0,69	155,57	0,00
Ala 0016	*Xanthosoma sp*.	-2,62	86,46	0,55	*Ipomoea batatas*	-2,89	86,99	0,42
Ala 0021	***Spondia dulcis***	0,04	31,26	0,98	Archeo	-3,76	38,86	0,02
Ala 0023	***Spondia dulcis***	5,13	20,88	1,00	*Zingiber_officinale*	-3,27	37,69	0,00
Ala 0024	***Ipomoea batatas***	0,88	12,81	0,92	*Manihot_esculenta*	-1,73	18,02	0,07
Ala 0027	*Ipomoea batatas*	0,46	10,45	0,57	*Manihot_esculenta*	0,17	11,03	0,42
Ala 0037	***Colocasia esculenta***	26,16	106,89	1,00	*Inocarpus_fagifer*	16,43	126,33	0,00
Ala 0039	***Manihot esculenta***	1,94	42,56	0,95	*Ipomoea_batatas*	-1,13	48,71	0,04
Ala 0041	***Canna* sp.**	3,51	75,19	0,97	*Ipomoea_batatas*	0,11	81,99	0,03
Ala 0043	***Artocarpus altilis***	9,52	7,34	0,99	*Manihot_esculenta*	4,47	17,43	0,01
Ala 0044	***Ipomoea batatas***	1,12	51,57	0,95	Archeo	-1,76	57,33	0,05
Ala 0045	*Dioscorea alata*	13,82	47,72	0,86	*Spondia_dulcis*	11,99	51,37	0,14
Ala 0046	***Zingiber officinale***	2,23	53,18	0,99	Archeo	-2,74	63,12	0,01
Ala 0047	***Zingiber officinale***	5,95	31,18	1,00	Archeo	-0,52	44,11	0,00
Ala 0048	***Spondia dulcis***	1,95	33,15	0,94	Archeo	-0,81	38,67	0,06
Ala 0049	*Manihot esculenta*	0,35	48,60	0,81	Archeo	-1,57	52,45	0,12
Ala 0050	**Archeo**	0,46	41,65	0,98	*Spondia dulcis*	-3,69	49,96	0,02
Ala 0051	***Zingiber officinale***	4,91	37,00	0,98	*Spondia dulcis*	0,72	45,39	0,01
Ala 0052	***Artocarpus altilis***	35,38	148,82	1,00	*Inocarpus fagifer*	24,42	170,75	0,00
Ala 0053	Archeo	-2,98	71,02	0,52	*Ipomoea batatas*	-3,06	71,18	0,48
Ala 0055	**Archeo**	-0,64	31,85	0,98	*Ipomoea batatas*	-4,70	39,96	0,02
Ala 0056	***Artocarpus altilis***	4,99	8,92	0,95	*Manihot esculenta*	1,84	15,23	0,04
Ala 0057	*Artocarpus altilis*	15,79	33,33	0,85	*Manihot esculenta*	14,04	36,83	0,15
Ala 0058	*Spondia dulcis*	0,57	27,43	0,82	Archeo	-0,97	30,51	0,18
Ala 0060	**Archeo**	-2,27	16,51	0,95	*Ipomoea batatas*	-5,27	22,51	0,05
Ala 0061	Archeo	1,95	79,13	0,66	*Curcuma longa*	1,23	80,57	0,32
Ala 0063	*Spondia dulcis*	1,23	21,13	0,85	Archeo	-0,50	24,59	0,15
Ala 0064	*Manihot esculenta*	0,46	13,37	0,49	*Ipomoea batatas*	0,44	13,41	0,48
Ala 0065	**Archeo**	0,30	45,52	0,99	*Ipomoea batatas*	-4,88	55,86	0,01
Ala 0066	*Ipomoea batatas*	-1,32	95,14	0,65	*Zingiber officinale*	-1,96	96,43	0,34
Ala 0071	**Archeo**	0,52	76,75	0,99	*Artocarpus altilis*	-5,56	88,92	0,00
Ala 0072	**Archeo**	1,01	62,59	1,00	*Ipomoea batatas*	-6,41	77,42	0,00
Ala 0073	*Ipomoea batatas*	-0,77	55,16	0,73	Archeo	-1,76	57,12	0,27
Ala 0074	**Archeo**	-0,37	61,11	1,00	*Xanthosoma* sp.	-7,12	74,61	0,00
Ala 0075	***Dioscorea alata***	20,47	56,15	1,00	*Spondia dulcis*	9,52	78,06	0,00
Ala 0077	**Archeo**	-0,3897	33,45	0,93	*Spondia dulcis*	-3,00	38,67	0,07

In bold and underlined 21 starch grains correctly assigned to species with certainty of correct assignment above 90% probability. The columns list the two highest scores among the classification functions for each of the observations used to fit the model (based on the 1240 observations of the reference collection) of the 46 observations of the archaeological starch grains. For example, in row 1 (starch grain Ala 002), the highest score corresponds to *Ipomoea batatas*, while the second highest score corresponds to *Manihot esculenta*. In bold, eight grains identified as Archeo indicate that closest similarty is with another archaeological starch grains.

#### Species identification *versus* archaeological artifacts

**Table 4** shows the number of starch grains found attached on each individual artifact. Artifact 322–4 registered the highest number of starch grains (n = 7). Starch grains on this artifact were assigned with over 90% probability to the following species: *A*. *altilis*, *S*. *dulcis*, and *Z*. *officinale*, while the identification of *M*. *esculenta* corresponded to a lower percentage (80%). Eight grains were most similar to other archaeological starch grains and therefore, could not be identified. Artifacts 316–1 and 322–7 had the second-highest number of starch grains attached (n = 6). Species found on artifact 316–1 belonged to the following: *A*. *altilis and Dioscorea sp*. with over 90% probability, while three grains were identified with low probability to *Ipomoea batatas*. All species on artifact 322–7 were identified with < 90% probability as *I*. *batatas*, *M*. *esculentat*, and *S*. *dulcis*. The remaining grains were most similar to other archaeological starch grains and could not be identified. Artifacts 322–3 and 322–9 each had four grains represented by the following species: Artifact 322–3, contained *A*. *altilis*, *I*. *batatas*, and *Z*. *officinale*, with over 90% probability, and *D*. *alata* with 84% probability. Artifact 322–9 had one grain of *D*. *alata*, one possible grain of *I*. *batatas*, and three unidentifiable grains. Artifacts 316–2, 316-3-316-4, and 322–5 had three grains each, while of the remaining six artifacts, one had two grains and the rest only a single starch grain. On three artifacts no measurable grains were found. For details of the distribution of all measurable starch grains on each artifact, see **Table 5**.

**Table 4:** The number of starch grains found on each of the archaeological artifacts.

Artifact Code	Artifact type	N° starch grains
0316–1	non-retouched flake	6
0316–2	non-retouched flake	3
0316–3	non-retouched flake	3
0316–4	non-retouched flake	3
0316–5	non-retouched flake	0
0316–6	non-retouched flake	1
0316–7	non-retouched flake	0
0316–8	non-retouched flake	0
0316–9	non-retouched flake	0
0322–1	non-retouched flake	1
0322–2	non-retouched flake	2
0322–3	non-retouched flake	4
0322–4	non-retouched flake	7
0322–5	non-retouched flake	3
0322–6	non-retouched flake	1
0322–7	non-retouched flake	6
0322–8	non-retouched flake	1
0322–9	non-retouched flake	4
0322–10	non-retouched flake	0
0322–11	non-retouched flake	1
Total number of grains: 46		

**Table 5:** Species distribution on archaeological artifacts and number of grains per artifact.

* *	316–1	316–2	316–3	316–4	316–6	322–1	322–2	322–3	322–4	322–5	322–6	322–7	322–8	322–9	322–11	total number of starch grains per species
*Artocarpus altilis*	2 **(05) (07)**							1 **(43)**	1 **(52)**	2 **(56)** (57)						6
*Canna* sp.			**1 (15**)				1 **(39)**									2
*Colocasia esculenta*						1 **(37)**										1
*Dioscorea alata*	1 **(06)**							1 (45)						1 (**75)**		3
*Inocarpus fagifer*		1 (11)														1
*Ipomoea batatas*	3 (02), (03) (10)			1 (24)	1(27)			1 **(44)**				1 (66)		1 (73)		8
*Manihot esculenta*							1 **(39)**		1 (49)			1 (64)				3
*Spondias dulcis*		2 (12) **(13)**	** **	2 **(21) (23)**					1 **(48)**	** **	1 58)	1 (63)				7
*Xanthosoma* sp.			1 (16)									* *		* *		1
*Zingiber officinale*	* *	* *	1 **(14)**	* *	* *	* *	* *	1 **(46)**	2 **(47) (51)**	** **	* *	* *	* *	* *	* *	4
*Archaeological starch*	* *	* *	** **	* *	* *	* *	* *	** **	2 **(50)** (53)	1 (55)	* *	3 **(60)** (61) (65)	1 (71)	1 (72) **(74)**	1 **(77)**	10
**Number of measurable starch grains on each artifact (n = 46)**	**6**	**3**	**3**	**3**	**1**	**1**	**2**	**4**	**7**	**3**	**1**	**6**	**1**	**4**	**1**	
**Numbers in parentheses indicate the Ala starch number.**						**Numbers in bold indicate species identification with >90% probability.**										

Note: *Musa* sp., *Curcuma longa*, and *Triticum aestivum* were not found.

Grains that matched other archaeological grains were examined visually. This observation and comparison with our reference collection permitted their identification based on the criteria described in **S1 Text** and in the descriptions published by Pagan [77]. Three of these grains could be assigned to *I*. *batatas* and three to *M*. *esculenta*. One grain could be assigned to *I*. *fagifer* and one to *Xanthosoma* sp. Finally, one grain showed features that could either be adscribed to *I*. *batatas* or to *M*. *esculenta*, because these two species have extensive overlaps, as mentioned earlier (**Fig 12**). No grains of *Musa* sp., *Curcuma longa*, and *Triticum aestivum* were found on any of the analyzed tools.

## Discussion

### Methodological aspects and successful species identification

As a starting point, we defined and standardized several variables, as suggested by Torrence et al. [75], Allen and Ussher [21], and Pagán [77] through images and explanatory drawings and complemented them with indexes we defined. Our aim was to develop a replicable methodology that would reduce the subjectivity of each observer as much as possible, particularly that inherent in qualitative parameters. The subjectivity unavoidably arises from the fact that each person’s brain selects and processes images in a unique way and therefore, builds an image that is almost impossible to reproduce by another individual [86].

The number of species represented in our reference collection, and the possibility of including additional species in the future, induced us to choose a method that would facilitate the classification of samples from unexpected sources, limit observer bias, and allow us to rely on the power of statistical tools. Considering that the aim of this study was to investigate the presence of common and uncommon starchy plant organs in Rapa Nui during early settlement, of either Pacific or American origin at initial colonization, our reference collection needed to be diverse, and include traditional crops from both regions. Although visual assessment of starch grains and comparison with a reference collection is a valid approach commonly used in the analysis of archaeological starch grains [see 87], we relied primarily on objective parameters and analysis for this study. Therefore, we discarded direct identification methods that solely rely on the analyst’s expertise, or a method based on the use of a limited number of variables, such as size, shape, and features of the hilum. As discussed by Leonel [84] and Horrocks & Wozniak [55], many taxa have starch grains that are similar in shape and size, in addition to the intraspecific variability that starch grains present, contrary to the idea that they have fixed and immutable characters. The starch identification method used in this study combines traditional morphological-size observations with two new metric indices and a two-step statistical analysis. We first conducted an exploratory unsupervised analysis of the data matrix from the reference collection and associated classification scheme. This resulted in a large overlap of starch morphotypes. To overcome this problem and increase the percentage of correctly assigned granules to each species, we reduced our data set to those grains in the reference collection data set that could accurately assign the allocation of unknown grains, a method previously proposed by Coster and Field [88], even though they used different algorithms. The second step consisted of establishing a classification algorithm in which only a subset of the data set was used, enabling us to increase correct species allocation to over 90% accuracy in almost fifty percent of the analyzed grains. This subset of the reference collection was then used for comparative purposes in the allocation of the archaeological grains.

The statistical analysis allowed us to discriminate between starch grains from species that present extensive morphological overlap between species and are very variable within species. By using a data subset of correctly classified grains using discriminant function analysis, we were able to discriminate between taxa, allowing unambiguous identification at the species level of 21 starch grains from the archaeological collection with a probability of >90% and five additional grains with over 80% probability. With this analysis ten plant species were identified, of which eight species were identified with >90% certainty (*A*. *altilis*, *Canna* sp., *C*. *esculenta*, *D*. *alata*, *I*. *batatas*, *M*. *esculenta*, *S*. *dulcis*, *and Z*. *officinale*, see **Table 3**).

A group of 26 grains could not be included in the statistical analysis either because they were partially damaged, or the total number of variables could not be measured. Ten grains could not be assigned by the statistical analysis to any species included in the reference collection, because they matched better to other archaeological grains than to any of the species in the reference collection. However, visual examination of these grains and comparison with our reference collection permitted their identification. These grains could be assigned to *I*. *batatas*, *M*. *esculenta*, *I*. *fagifer*, and *Xanthosoma* sp.

### Cultural use of obsidian artifacts that hold starch grains

The number of starch grains found on each individual tool is small compared to the number of starch grains reported in other case studies [e.g. 89, 90]. However, most of these studies extracted starch grains from coarse-grained grinding or pounding stone tools, where the starch grains got trapped in cracks or crevices found on the surface of these tools. In our case, we were dealing with natural glass flakes with smooth surfaces and only some striation and scarring on the edges due to use wear, in which starch grains can get trapped [91, 92]. It is therefore not unlikely that the number of starch grains that adhere to these surfaces is much smaller than those recovered from coarser materials.

With regards to the processing of plant remains, the presence of mostly unmodified starches is indicative that these obsidian tools were used mainly for the processing of raw organs. The presence of unmodified starch grains suggests that the tools were used for processing tubers, rhizomes, fruits, and/or kernels before cooking. The presence of multiple species on a single artifact implies that these small flakes were multipurpose tools that were possibly used repeatedly and not washed between uses. This could have involved either cutting, scraping off the skins, or some other type of processing. In traditional Polynesian cooking, food may need to be husked, scraped, peeled, or grated before transforming into a pudding (*po’e*), or before grilling, roasting, or placing inside the earth oven [93]. Most of the species identified in this study required cooking to make them edible; the only exception is sweet potato (*I*. *batatas*), which can be consumed raw. Interestingly, Tromp & Dudgeon [43] extracted starch from dental calculus from Rapanui human remains and only found remains of *I*. *batatas* that had been consumed raw.

### Identification of Pacific species from the early settlement site on Rapa Nui

The literature indicates that over 300 plant species were transported at one time or another across the Pacific as part of the west-to-east colonization process. Of these, only about 20 to 30 species are supposed to have reached Rapa Nui [13]. Whistler [14] has posited that the cooler climate encountered by the early colonists in southern Polynesia constrained tropical crop production, such as taro, breadfruit, or *I*. *fagifer*. However, in the southern Polynesian islands of Rapa and Raivavae, taro was grown successfully in the lowland areas of these islands [20, 94].

Our results show the presence of species that are part of the traditional Rapa Nui crop inventory, such as *D*. *alata* and *C*. *esculenta*, a subset of the canoe plants previously recorded for this island. In addition, we found some species not previously recorded for Rapa Nui, such as *A*. *altilis*, *S*. *dulcis*, and *Z*. *officinale*. The first two species are known to have reached other Pacific Islands but have not been mentioned either in oral tradition or historic reports as being part of the plants or crops that reached Rapa Nui. On the other hand, the finding of *Z*. *officinale* is, to our knowledge, the first report for Remote Oceania and will be discussed below.

The presence of grains from both *Dioscorea* sp. (n = 3, with over 80% probability) and *C*. *esculenta* (n = 1, with 99% probability) is in line with similar studies for the rest of the Pacific, where they are the predominant species [95–99]. The local tradition mentions both crops, but particularly *Dioscorea* sp., as one of the cultivated plants already present on the island when the first emissaries from the founding king, Hotu A Matu’a, visited the island of Rapa Nui [11]. Martinsson-Wallin’s research in the Vinapu area [100, 101] has indicated that elongated holes found in prehistoric contexts could indicate the cultivation of *Dioscorea* sp. or yams.

The unexpected finding of three species, i.e., breadfruit (*A*. *altilis*), Tahitian apple (*S*. *dulcis*), and ginger (*Z*. *officinale*), not previously recorded for Rapa Nui, requires some discussion. Breadfruit is thought to have been domesticated somewhere in Island Southeast Asia and western Melanesia, including New Guinea [102, 103] and was dispersed by humans since at least early Lapita times [104, 105], is one of the most important food plants of the Pacific. According to the literature, breadfruit is not considered part of the suite of plants that reached Rapa Nui. Some authors have discussed that, if ever present, it did not survive because of the subtropical climate conditions of the island [106]. There are only two previous archaeological records for the presence of breadfruit in the wider region of East Polynesia. The first corresponds to starch grains attached to shell artifacts dated to 1400 to 1600 AD in the Marquesas [21], and the second corresponds to charred exocarp remains from late contexts of the Society Islands [107]. Our findings therefore represent the third, and earliest record of this species in the archaeological context of East Polynesia. Breadfruit was a central crop in East Polynesia at the time of European contact, both as a staple for food and for its wood, which was used in the construction of elite or ritual buildings and the carving of sacred objects [see 108, 109]. Carpological remains of breadfruit have also been identified in 13th-14th century archaeological contexts on Maupiti islands [18], Mangareva [110], and Hawaii [111].

The presence of breadfruit starch on the tools from Anakena provides the first evidence that this species was introduced to Easter Island as part of the colonizing strategy. It probably survived and grew for several generations before disappearing. Until now, we lacked physical evidence (macro or microremains) for its presence in antiquity. On Rapa Nui today, this tree grows and bears fruit, but islanders hardly ever eat it and it does not form part of the staple diet. The available climate data bases indicate that between ca. 800 AD and 1200 AD, the island was affected by the warmer conditions of the Medieval Climate Anomaly, after which temperatures dropped at the onset of the Little Ice Age [112]. Although other explanations can be advanced, it is possible that the drop in temperatures beginning in 1300 AD may have caused the disappearance of this and other species. Breadfruit is represented by four clearly identified starch grains with an over 90% probability.

The presence of *S*. *dulcis* on Rapa Nui, another widely distributed Polynesian crop, is also surprising. This species, also known as Tahitian or golden apple, grows best in the subhumid and frost-free tropics with temperatures not above 27°C, where it is found from sea level up to 700 meters of altitude. *Spondias* does not require cooking for human consumption. To our knowledge, no previous record of the presence of this tree exists for Rapa Nui. Today, in East Polynesia, *S*. *dulcis* grows along the edges of old gardens, riverbanks, coastal areas, and coconut plantations. *Spondias dulcis* has been identified through anthracological studies in the Society Islands [113] as a tree commonly used for fuel. This species is represented by four distinctly identified starch grains (>90% probability).

Ginger (*Z*. *officinale*) is one of the 50 to 60 species of the *Zingiber* genus that grow in the tropics, from Asia to New Guinea [114]. The original homeland is uncertain as ginger no longer grows wild [115, 116], and the origin of its domestication is unclear, but some species of the genus may be native to the Pacific Islands [114]. Ginger has been used as medicine and a spice in India and China since ancient times [116]. In India, ginger starch has been identified at the site of Farmana [117], and in China, ginger starch granules have been identified at the Neolithic Dadiwan site [118], and Liu et al. [119] found starch remains associated with the production of alcoholic beverages in containers dating to 9000–7000 cal BP. Recently, ginger starch grains have been identified on grinding stones from Vietnam [117]. Ginger was one of the first Oriental spices to reach the Greeks and Romans about 2000 years ago, was known in France and Germany since the ninth century, England since the tenth century [115, 116] and reached Scandinavia only in the 15^th^ century [120]. However, information about its eastward spread is scant. Kaushal et al. [121] state that ginger is one of the traditional medicinal plants that have been used for over 2000 years by Polynesians to treat diabetes, cancer, and many other diseases. Lepofsky [122] comments that in the Society Islands, wild ginger (*Z*. *zerumbet*) was probably more common in the past than it is today, and the early observers were largely silent regarding ginger in general. The same author remarks that ginger was probably cultivated, based on its association with archaeological sites.

The finding of four archaeological starch grains identified with high probability (>90%) as ginger on three obsidian tools is, to our knowledge, the first evidence that this species was introduced to Easter Island during early settlement. As stated above, this result was unexpected, particularly as this species is not mentioned in legends, nor was it described by any of the early European explorers or visitors to Rapa Nui. Although there might be previous clues for this highly valued spice in the Society Islands, we consider this result with great caution while waiting for additional and independent evidence for its presence on Rapa Nui.

Finally, one grain was visually identified as belonging to *Inocarpus fagifer*, or the Tahitian chestnut. This tree is commonly found in Oceania and grows mainly in lowland areas associated with riverbanks along the shoreline or close to coconut plantations. It is planted for its wood and nuts, which need cooking to make them edible. According to Pauku [123], it was cultivated more intensively in the past than today. The starch grains of these species have an important overlap with the grains of *I*. *batatas*, and therefore, we need to exercise caution regarding this identification.

### Identification of American species from the earliest settlement site on Rapa Nui

We also found evidence for three species of South American origin, commonly not recognized as part of the prehistoric starchy foods available on this island. These are *Canna* sp. (achira), *I*. *batatas* (sweet potato or *kumara*), and *M*. *esculenta* (manioc). First, we will discuss the case of *Canna* sp., or “achira”, native to South America and the Caribbean. It is possibly one of the very early domesticates of the Northeastern South American region [124]. Even today, in Ecuador and Colombia, *C*. *edulis* roots provide starch for cooking, and the leaves are used for wrapping food. Its roots can also be eaten raw. *C*. *indica* is a close relative of *C*. *edulis*, is grown for its flowers, and is found today on many Pacific Islands.

The Spanish Expedition to Easter Island in 1770 mentions the presence of achira. The cartographer José de Moraleda, writing from Chiloé in January 1771, stated explicitly that *achiraf* (sic) was one of the Easter Islanders’ food items [74]. Achira is also mentioned in one of the maps signed by Juan Hervé of the same expedition and reproduced by Mellén-Blanco [67], which includes several cultivated plant species. Also, according to a letter written later by the Viceroy Amat to his successor, Viceroy Manuel de Guirior in 1761, achira is on the list of species described for the island [74].

Langdon [125], discussing the early presence of both achira and manioc on Rapa Nui, raised the possibility that some of these crops may have been misidentified. However, the Spanish probably recognized the few South American plant species observed. Langdon [125] has pointed out that they might have confused turmeric with achira since they belong to the same plant family. Nonetheless, they were quite clear in stating that one of these was used to stain cloth (turmeric), and the other was used as a food item. Unlike turmeric, achira is not known to produce a yellow dye. *Canna* sp. is represented by two starch grains identified with 96% and 100% probability.

Sweet potato (*I*. *batatas)* and manioc (*M*. *esculenta)* starch grains exhibit a substantial morphological overlap, as shown in **Fig 5A**. The presence of sweet potato in archaeological contexts in the Pacific has been subject to extensive scientific discussion, as for a long time it was the sole evidence regarding contact between Polynesia and South America[e.g., references in 52, 87, 126–128]. Barrau [129] originally proposed that its presence in the Pacific was the result of three independent introductions. This hypothesis was further discussed and developed by Yen [130] and Green [131] and taken up again by Roullier et al. [52] based on genetic studies of the different variants introduced in prehistoric and historic times across the Pacific. The genetic and archaeological evidence indicate that the first introduction and wide dispersal of this species throughout East Polynesia occurred on the west coast of South America and took place around 1000–1100 AD [131]. Sweet potatoes could have been introduced into the Pacific as early as 1200–1400 AD, as reported by Allen and Ussher [21] in the Marquesas. Additionally, archaeological remains from Mangaia Island in the Cook Islands have been dated to 1361–1466 AD [132] and in Hawaii to 1297–1422 AD [133]. On Rapa Nui, Skjølvold [134] described finding carbonized remains of sweet potato, sugar cane, and makoi nuts at site E-2 in Anakena which were dated to 1437–1619 AD [135]. Horrocks and Wozniak [55] dated sweet potatoe starch grains from a garden at Te Niu, obtaining dates both older and younger than 1400 AD. The available evidence so far suggests an introduction around 1200–1300 AD, in conjunction with the appearance of new types of archaeological features [136]. Additional radiocarbon dates of 1322–1438 AD (2 sigma, NZA-37006, 587±30 BP) were reported by Horrocks et al. [50].

The identification of sweet potato starch grains in the lower levels of the Anakena site suggests an introduction of this species to Rapa Nui during the earliest settlement period. However, our results call for caution because, although we found 11 starch grains, only two of them were identified with over 90% probability (see **Table 5**). Six grains were identified with low probability and overlap with *M*. *esculenta*, and three were identified visually. We need to point out that another species of the genus, *Ipomoea pes-caprae*, is indigenous to the island [137]. Ethnographic information indicates that stems, leaves, and roots were consumed as famine food [106, 137]. As *I*. *pes-caprae* does not have underground starchy organs, the starch grains found in this study cannot be attributed to the indigenous species (See http://legacy.tropicos.org/Image/101438018). In addition, our assessment considers the characteristics of the morphotypes described by us for *I*. *batatas* roots (see **S1 Text**) that are like the ones described by other authors and used to distinguish *I*. *batatas* starch from other Polynesian geophytes.

*Manihot esculenta* is the third of the South American species identified in this study. It is generally known by the names of cassava, mandioca, or manioc; the common indigenous name in the Andean region of Peru and Ecuador, and Northern South America is *yuca* [138]. It has generally been assumed that manioc was introduced by missionaries into the Pacific in the 1850s [139, 140]. After the Spanish Expedition of 1770, the presence of manioc on the island was only noted again in 1911 by Martínez [141] and Fuentes [142], both members of the 1911 Chilean scientific expedition to Rapa Nui [143], who saw it growing in the islanders´ garden plots. It was also mentioned later by other scholars [10, 106]. Whether the plants seen in the early 20th century are descendants of old stock or recent re-introductions from Tahiti, as posed by Barrau [139], is still an open question. Métraux [106] considered manioc to be a recent European introduction and lists it together with peas, melons, onions, and the like, while Heyerdahl and Ferdon [10] considered that the evidence was not conclusive. This controversy can only be assessed through direct genetic analyses of the plant samples collected by Fuentes for the Herbarium of the National Natural History Museum in Santiago (SGO). Blixen [144] posed that if the identification of manioc by the Spanish was correct, this would signify its presence on the island since times long preceding a modern introduction from Tahiti. This hypothesis is now better supported by our findings, but our results for this species are still not conclusive. On the other hand, recent introductions cannot be excluded.

We need to be cautious with the identification of this species; the allocation score of starch grains to this species is of two grains, of which only one is identified with over 90% probability. Three additional grains were identified visually based on specific characteristics of this species, such as the size and shape of the grains, an open hilum with curvy or y-shaped fissures, and mixed pressure facettes.

Finally, the visual examination also shows the presence of one grain assigned to *Xanthosoma* sp., which is one of the many edible plants in the Aroid family. In the Pacific, it is commonly referred to or confused with taro and is consumed very much like it [145]. This is also the case for Rapa Nui, where *Xanthosoma* sp. plants are known under the common name “*taro vaihi*”, from where our reference material originates. The native distribution range of the most common edible species (*X*. *sagittifolium*) is unclear, but it has been suggested that it is native to northern South America, comprising Colombia, Peru, Ecuador, and Venezuela [146, 147]. In this region, it is commonly known under the names “*malanga*” or “*yautía*” [148]. Almost nothing is known about its introduction into the Pacific Islands, and it has commonly been assumed that its introduction dates to historic times. Today, it is even considered an invasive species on many islands [149]. Pollock [150] also mentioned *Xanthosoma* as one of the “new introductions” to the Tahitian food inventory. In this study, it is represented only by one starch grain that shows the characteristic features of this species in our collection, such as its size, bell shape, and open hilum with a large fissure. Our study shows for the first time that this crop might have been present on the island in pre-European times. It could have been carried on the canoes on the return voyages from the continent, in particular because of its similarities to taro.

The introduction of these South American species strongly suggests that some type of contact was established between Polynesian seafarers and South American populations. Our findings provide the first evidence that these species could have been introduced much earlier than previously thought, as part of a package of South American plants that reached the Pacific during return voyages from the Americas (see, for example, Ioannides et al. [151], on new human genetic studies, as discussed below). It is most likely that the species chosen for a return voyage would be those whose edible parts would travel well or that could be transported easily as potted plants. An additional property of all these species is that they can be propagated vegetatively, in accordance with the cultural practices of tropical Pacific agriculture. These plants were seen and described by the Spanish who visited Easter Island in 1770. In his logbook, Juan Hervé, commander of the San Lorenzo ship, refers to sweet potatoes, sugar cane, and bananas, but also to yuca, ñame, and red and white gourds [74]. The term *ñame* refers to yams (*Dioscorea* sp.), while yuca is the common indigenous name for manioc in the Andean region. This log is the earliest mention of manioc on the island [125].

Our general results strengthen the evidence of contacts between Pacific Island peoples and South American populations. The proof of when this process took place and the number and intensity of these contact events have been discussed repeatedly in the literature since at least the 1920s [e.g., 152–155]. More recently, this topic has resurfaced with new studies [e.g. 87, 151, 156–166] however, there is still little evidence of the nature and intensity of the interaction between Polynesians and American populations. By including a set of South American tuber plants in our comparative collection, we provide the first empirical evidence that *I*. *batatas* was not the only food transported from the Americas into the Pacific. The presence of *Canna* sp. and possibly manioc starch grains, offers the first evidence that these species were introduced to the Pacific islands in prehistoric times. Whether their introduction was directly to Rapa Nui or via other islands can only be addressed through further studies that actively search for these plant remains in the wider Pacific. Recently, evidence from human genetic studies has been accumulating for a sustained interaction between Pacific and American populations. One of the scenarios posed by Ioannides et al. [151] is that groups of Polynesian people voyaged back from specific localities in northern South America, with or without American people on board, suggesting interactions on the American continent. Likely landing points for return voyages from the Americas may initially have been Fatu Hiva, as suggested by these authors, and from there to some other islands, including Rapa Nui. For return voyages, boats needed to be stocked with sufficient food for several weeks. These crops may also have been transported as live plants, in accordance with the Polynesian voyaging tradition. The finding of starch grains on Rapa Nui of these species implies the translocation of surplus living specimens or organs for planting on arrival. Most of these species reproduce vegetatively through their edible underground tubers, corms, and/or rhizomes. In our opinion, a fleeting or single encounter seems highly improbable or unlikely for the prehistoric introduction of a suit of edible crops from the American coast to the Pacific islands, because the sharing of knowledge and resources requires some sustained interaction. Our results contribute independent evidence to this likely scenario. The use of common words in South American and Polynesian languages, like “kumara” for sweet potato and others not related to plants [167], suggests sustained (and at least partly peaceful) interactions. Our results show that the menu of the first voyagers and colonizers living at the Anakena site was much more varied than previously assumed. Their menu of staples included not only the traditional Polynesian canoe plants, but also several tuberous crops native to South America. This study also provides independent support for the general conclusions reached by recent human genetic studies [151], which indicate direct contact between Pacific populations and individuals from northern South America.

Finally, we would like to stress that it is important that future studies in this field contemplate including a large range and variety of starchy species from both Pacific and South American origins, such as the ones sampled here. A robust and varied reference collection is instrumental in discovering staples that otherwise would go unnoticed. Our findings do not preclude the transport of additional plant species that remain invisible in current reference collections.

## Supporting information

S1 Fig Explanatory figure to show how measurements were taken. A. Maximum and minimum length of starch grains. B. Maximum and minimum distance of hilum to the rim of the grain. C. Perimeter and total area of the grain.
(TIF)

S2 Fig Categorization of the 2-dimensional shapes used in the description of the starch grains. Scale bars correspond to 10 μm.
(TIF)

S3 Fig Descriptive images of qualititative variables measured in individual starch grains. A. Style of the extinction cross, where A1 = curvy, A2 = straight, and A3 = wavy. B. Shape of the hilum fissure, in which B1 = absent, B2 = circular, B3 = simple, B4 = v shape, B5 = y shape, B6 square, B7 = hat shaped, and B8 = elongated. C. Shape of grain facettes, where C1 = absent, C2 = flat, C3 = Concave, C4 = multi-facetted, C5 = multi-concave, and C6 = Multi-mixed.
(TIF)

S4 Fig Non-measurable starch grains discarded from the statistical analysis. A- D Damaged grains, under polarized and brightfield light. E-F Starch grains found inside the nail polish on the edge of the slides. Scale bars = 10 μm.
(TIF)

S1 File Tables with the statistical summary of quantitative variables of the reference collection data set, quantitative measurements and categorization of qualitative variables taken on archaeological starch grains, and eigenvalues and percentages of variation as explained by the principal components for each of the 15 variables analyzed. (DOCX)

S1 Text Description of the characteristics of morphotypes associated to species in the reference collection. (DOCX)

## References

[1] Hunt TL, Lipo CP. Late colonization of Easter Island. Science. 2006; 311:1603–1606. doi: 10.1126/science.1121879 16527931

[2] Wilmshurst J, Anderson AJ, Higham TFG, Worthy TH. Dating the late prehistoric dispersal of Polynesians to New Zealand using the commensal Pacific rat. P Natl Acad Sci. 2008; 105 (22): 676–7680. doi: 10.1073/pnas.0801507105 18523023 PMC2409139

[3] Mulrooney MA. An island-wide assessment of the chronology of settlement and land use on Rapa Nui (Easter Island) based on radiocarbon data. J Archaeol Sci. 2013; 40:. 4377–4399. doi: 10.1016/j.jas.2013.06.020

[4] Jacomb C, Holdaway RN, Allentoft ME, Bunce M, Oskam CL, Walter R, et al. High-precision dating and ancient DNA profiling of moa (Aves: Dinornithiformes) eggshell documents a complex feature at Wairau Bar and refines the chronology of New Zealand settlement by Polynesians. J Archaeol Sci. 2014; 50: 24–30. doi: 10.1016/j.jas.2014.05.023

[5] DiNapoli R, Rieth T, Lipo C, Hunt T. A model-based approach to the tempo of “collapse”: The case of Rapa Nui (Easter Island). J Archaeol Sci. 2020; 116:105094. doi: 10.1016/j.jas.2020.105094

[6] Wallin P, Martinsson-Wallin H. Anakena Re-visited: New Perspectives on Old problems at Anakena, Rapa Nui. In: Rull V, Stevenson CM. Editors. The Prehistory of Easter Island (Rapa Nui): Towards an Integrative Interdisciplinary Framework. New York: Springer, Cham. 2022. Pp. 109–140. doi: 10.1007/978-3-030-91127-0_6

[7] Irwin G. The Prehistoric Exploration and Colonization of the Pacific. Cambridge: Cambridge University Press. 1992.

[8] Anderson A. Current approaches in East Polynesian colonization research. J Polynesian Soc. 1995; 104 (1): 110–132.

[9] Sear DA, Allen MS, Hassall JD, Maloney A, Langdon PG, Morrison A, et al. Human settlement of East Polynesia earlier, incremental, and coincident with prolonged South Pacific drought. P Natl Acad Sci. 2020; 117 (16): 8813–8819. doi: 10.1073/pnas.1920975117 32253300 PMC7183181

[10] Heyerdahl T, Ferdon E. Reports of the Norwegian Archaeological Expedition to Easter Island and the East Pacific. Vol. 1. Archeology of Easter Island. Monograph of the School of American Research and the Kon-Tiki Museum no. 24. part. 1. Stockholm: Forum publishing. 1961.

[11] Barthel T. The Eighth Land: The Polynesian Discovery and Settlement of Easter Island. Honolulu: University Press of Hawai’i, 1978.

[12] Horsburgh A, McCoy MD. Dispersal, Isolation, and Interaction in the Islands of Polynesia: A Critical Review of Archaeological and Genetic Evidence. Diversity. 2017; 9(3):37. doi: 10.3390/d9030037

[13] Banack SA. Plants and Polynesian voyaging. In: Cox PA, Banack SA. editors. Island Plants, and Polynesians. Oregon: Dioscorides Press. 1991. pp 25–40.

[14] Whistler WA. Polynesian plant introductions. In: Cox P.A., Banack S.A., Islands, Plants and Polynesians: An introduction to Polynesian ethnobotany, Dioscorides Press, Portland. 1991. pp. 41–66.

[15] Kirch P V. The wet and the dry: irrigation and agricultural intensification in Polynesia. Chicago: University of Chicago Press. 1994.

[16] Prebble M, Wilmshurst JM. Detecting the initial impact of humans and introduced species on island environments in Remote Oceania using palaeoecology. Biol Invasions. 2009:11: 1529–1556. doi: 10.1007/s10530-008-9405-0

[17] Maxwell JJ, Howarth JD, Vandergoes MJ, Jacobsen GE, Barber IG. The timing and importance of arboriculture and agroforestry in a temperate East Polynesia Society, the Moriori, Rekohu (Chatham Island). Quat. Sci. Rev. 2016; 149: 306–325. doi: 10.1016/j.quascirev.2016.08.006

[18] Dotte-Sarout E, Kahn JG. Ancient woodlands of Polynesia: a pilot anthracological study on Maupiti Island, French Polynesia. Quat Intern. 2017; 457: 6–28. doi: 10.1016/j.quaint.2016.10.032

[19] Torrence R, Barton H. Ancient Starch Research. Walnut Creek: Left Coast Press. 2006.

[20] Prebble M, Anderson AJ, Emmitt PAJ, Fallond SJ, Furey LL, Holdaway SJ, et al. Early tropical crop production in marginal subtropical and temperate Polynesia. P Natl Acad Sci. 2019; 116 (18): 8824–8833. doi: 10.1073/pnas.1821732116 30962379 PMC6500154

[21] Allen M, Ussher E. Starch analysis reveals prehistoric plant translocations and shell tool use, Marquesas Islands. Polynesia. J Archaeol Sci. 2013; 40:2799–2812. doi: 10.1016/j.jas.2013.02.011

[22] Flenley JR, King M. Late Quaternary pollen records from Easter Island. Nature. 1984; 307: 47–50. doi: 10.1038/307047a0

[23] Bahn PG, Flenley JR. Easter Island, Earth Island. London: Thames and Hudson. 1992.

[24] Diamond J. Collapse: How societies choose to fail or succeed. New York: Viking Press. 2005.

[25] Hunt TL. Rethinking the fall of Easter Island. Am. Scientist. 2007; 94:412–419. doi: 10.1511/2006.61.412

[26] Mann D, Edwards J, Chase J, Beck W, Reanier R, Mass M, et al. Drought, vegetation change and human history on Rapa Nui (Isla de Pascua, Easter Island). Quatern Res. 2008; 69: 16–28. doi: 10.1016/j.yqres.2007.10.009

[27] Rull V, Cañellas-Boltà N, Sáez A, Giralt S, Margalef O. Paleoecology of Easter Island: Evidence and uncertainties. Earth-Science Rev. 2010; 99: 50–60. doi: 10.1016/j.earscirev.2010.02.003

[28] Martinsson-Wallin H, Wallin P. Excavation at Anakena. The Easter Island Settlement Sequence and Change of Subsistence? In: Vargas P. Editor. Easter Island and East Polynesian Prehistory. Santiago, Chile: Universidad de Chile, Facultad de Arquitectura y Urbanismo, Instituto de Estudios Isla de Pascua. 1999. Pp.179–186.

[29] Martinsson-Wallin H, Crockford SJ. Early settlement of Rapa Nui (Easter Island). Asian Perspec 2001; 40(2): 244–278. https://www.jstor.org/stable/42928504.

[30] Mulrooney M, Ladefoged T, Stevenson C, Haoa S The myth of A.D. 1680: New evidence from Hanga Ho’Onu, Rapa Nui (Easter Island). Rapa Nui J. 2009; 23(2):94–05.

[31] Wallin P, Martinsson-Wallin H, Possnert G. Re-dating Ahu Nau Nau and the Settlement at ‘Anakena, Rapa Nui. In: Wallin P, Martinsson-Wallin H. editors. The Gotland Papers: Selected Papers from the VII International Conference on Easter Island and the Pacific: Migration, Identity, and Cultural Heritage. Visby: Gotland University. 2010. pp. 37–46.

[32] Flenley JR. The late quaternary vegetational and climatic history of Easter Island. J Quaternary Sci. 1991; 6:85: 115. doi: 10.1002/jqs.3390060202

[33] Nunn PD. Environmental catastrophe in the Pacific islands around A.D. 1300. Geoarchaeol. 2000; 15:715–740. doi: 10.1002/1520-6548

[34] Orliac C, Orliac M. La flore disparue de l’île de Pâques. Les Nouvelles de l’Archéologie 2005; 10: 29–33.

[35] Mieth A, Bork HR. Humans, climate or introduced rats—which is to blame for the woodland destruction on prehistoric Rapa Nui (Easter Island)? J Archaeol Sci. 2010; 37: 417–426. doi: 10.1016/j.jas.2009.10.006

[36] Horrocks M, Baisden WT, Nieuwoudt MK, Flenley J, Feek D, González L, et al. Microfossils of Polynesian cultigens in lake sediment cores from Rano Kau, Easter Island. Journal of Paleolimnology 2012; 47:185–204. doi: 10.1007/s10933-012-9643-0

[37] Cañellas-Boltà N, Rull V, Sáez A, Margalef O, Bao R, Pla-Rabes S, et al. Vegetation changes and human settlement of Easter Island during the Last millennia: a multiproxy study of the Lake Raraku sediments. Quat Sci Rev. 2013; 72: 36–48. doi: 10.1016/j.quascirev.2013.04.004

[38] Rull V, Cañellas-Boltà N, Margalef O, Pla-Rabes S, Sáez A, Giralt S. Three Millennia of Climatic, Ecological, and Cultural Change on Easter Island: An Integrative Overview. Front. Ecol. Evol. 2016; 4:29. doi: 10.3389/fevo.2016.00029

[39] Rull V. The deforestation of Easter Island. Biol Rev. 2020; 95(1):124–141. doi: 10.1111/brv.12556 31599482

[40] Rull V. Contributions of paleoecology to Easter Island’s prehistory: A thorough review. Quat Sci Rev 2021; 252:106751. doi: 10.1016/j.quascirev.2020.106751

[41] Leach F, Quinn C, Morrison J, Lyon G. The Use of Multiple Isotope Signatures in Reconstructing Prehistoric Human Diet from Archaeological Bone from the Pacific and New Zealand. NZ J Archaeol. 2003; 23:31–98, Available at: https://ssrn.com/abstract=2198511

[42] Commendador A, Dudgeon JV, Finney B, Fuller B, Esh K. A Stable Isotope (d13C and d15N) Perspective on Human Diet on Rapa Nui (Easter Island) ca. AD 1400–1900. Am J Physical Anthrop. 2013; 152: 173–185. doi: 10.1002/ajpa.22339 23996514

[43] Tromp M, Dudgeon JV. Differentiating dietary and non-dietary microfossils extracted from human dental calculus: The importance of sweet potato to ancient diet on Rapa Nui. J. Archaeol. Sci. 2015; 54:54–63. doi: 10.1016/j.jas.2014.11.024

[44] Jarman C, Larsen T, Hunt T, Lipo C. Solsvik R, Wallsgrove N, Lyons C, Close H, Popp B. Diet of the prehistoric population of Rapa Nui (Easter Island, Chile) shows environmental adaptation and resilience. Am J Phys Anthrop. 2017; 164:343–361. doi: 10.1002/ajpa.23273 28664976 PMC5637906

[45] Polet C, Bocherens H. New insights into the marine contribution to ancient Easter Islanders’ diet. J Archaeol Sci Rep. 2016; 6: 709–719. doi: 10.1016/j.jasrep.2015.09.013

[46] Commendador A, Finney B, Fuller B, Tromp M, Dudgeon JV. Multiproxy isotopic analyses of human skeletal material from Rapa Nui: Evaluating the evidence from carbonates, bulk collagen, and amino acids. Am J Physical Anthrop. 2019; 169 (4): 714–729. doi: 10.1002/ajpa.23851 31062347

[47] Wozniak J. Palm Forest to Gardens and Grassland: A Study of Environmental and Geomorphological Changes of the Te Niu, Rapa Nui Landscape. In: Rull V, Stevenson CM. Editors. The Prehistory of Easter Island (Rapa Nui): Towards an Integrative Interdisciplinary Framework. New York: Springer. 2022. Pp. 449–480.

[48] Flenley JR, Bahn P. Conflicting views of Easter Island. Rapa Nui J. 2007; 21:11–13. https://kahualike.manoa.hawaii.edu/rnj/vol21/iss1/4

[49] Bork H, Mieth A. The key role of *Jubaea* palm trees in the history of Rapa Nui: A provocative interpretation. Rapa Nui J. 2003; 17(2): 119–122.

[50] Horrocks M, Baisden T, Flenley J, Feek D, González-Nualart L, Haoa-Cardinali S, et al. Fossil plant remains at Rano Raraku, Easter Island’s statue quarry: Evidence for past elevated lake level and ancient Polynesian agriculture. J. Paleolimnol. 2012; 38:767–783. doi: 10.1007/s10933-012-9643-0

[51] Clarke A, Burtenshaw M, McLenachan P, Erickson D, Penny D. Reconstructing the origins and dispersal of the Polynesian bottle gourd (*Lagenaria siceraria*). Mol Biolo & Evol. 2006. 23: 893–900. doi: 10.1093/molbev/msj092 16401685

[52] Roullier C, Kambouo R, Paofa J, McKey D, Lebot V. On the origin of sweet potato (*Ipomoea batatas* (L.) Lam.) genetic diversity in New Guinea, a secondary centre of diversity. Heredity 2013; 110: 594–604. doi: 10.1038/hdy.2013.14 23531982 PMC3656641

[53] Orliac C. Données nouvelles sur la composition de la flore de l’île de Pâques. J Soc des Océanistes. 1998; 107(2):135–143. doi: 10.3406/jso.1998.2053

[54] Cummings LS. A review of recent pollen and phytolith studies from various contexts on Easter Island. In: Stevenson C, Lee G, Morin F. editors. Easter Island in Pacific Context, Los Osos, California: Easter Island Foundation. 1998. pp. 100–106.

[55] Horrocks M, Wozniak J. Plant microfossil analysis reveals disturbed forest and a mixed-crop, dryland production system at Te Niu, Easter Island. J Archaeol Sci. 2008; 35: 26–142. doi: 10.1016/j.jas.2007.02.014

[56] Sherwood SC, Van Tilburg JA, Barrier CR, Horrocks M, Dunn RK, Ramírez-Aliaga JM. New excavations in Easter Island’s statue quarry: Soil fertility, site formation and chronology. J. Archaeol Science. 2019; 111:104994. doi: /10.1016/j.jas.2019.104994

[57] Martinsson-Wallin H, Wallin P. The Settlement Activity Area Nau Nau East at Anakena, Easter Island. In: Skjøllsvold A. Editor. Archaeological Excavation at Anakena Easter Island. The Kon-Tiki Museum Occasional Papers 3. Oslo. Norway: The Kon-Tiki Museum Institute for Pacific Archaeology and Cultural History. 1994. Pp. 112–216.

[58] Skjølsvold A. Archaeological investigations at Anakena, Easter Island. In: Skjolsvold A. editor. The Kon-Tiki Museum Occasional Papers vol. 3. Oslo: The Kon-Tiki Museum. 1994. pp. 5–121.

[59] Vargas P, Cristino C, Izaurieta R. 1000 años en Rapa Nui: Arqueología del asentamiento. Santiago: Editorial Universitaria. 2006.

[60] Martinsson-Wallin H, Wallin P, Anderson A, Solsvik R. Chronogeographic Variation in Initial East Polynesian Construction of Monumental Ceremonial Sites. J of Isl & Coast Archaeol. 2013; 8(3): 405–421. doi: 10.1080/15564894.2013.834009

[61] Allen M. Spatial variability and human eco-dynamics in central East Polynesian fisheries. In: Albarella U, Rizzetto M, Russ H, Vickers K, Viner-Daniels S. editors. The Oxford Handbook of Zooarchaeology. Oxford Handbooks Online, Oxford, UK: Oxford University Press. 2017. Pp. 737–756. doi: 10.1093/oxfordhb/9780199686476.013.51

[62] Crowther A, Haslam M, Oakden N, Walde D, Mercader J. Documenting contamination in ancient starch laboratories. J. Archaeol. Sci. 2014; 49: 90–104. doi: 10.1016/j.jas.2014.04.023

[63] Haslam M. The decomposition of starch grains in soils: implications for archaeological residue analyses. J. Archaeol. Sci. 2004; 31, 1715–1734. doi: 10.1016/j.jas.2004.05.006

[64] Laurence AR., Alston VT, Vaughn MB, McDonough C. Airborne Starch Granules as a Potential Contamination Source at Archaeological Sites. Journal of Ethnobiology. 2011; 31(2):213–232. doi: 10.2993/0278-0771-31.2.213

[65] Sikoparija B, Matavulj P, Mimic G, Smith M, Grewling L, and Podrascanin Z. Real-time automatic detection of starch particles in ambient air. Agricultural and Forest Meteorology 2022; 323:109034. doi: 10.1016/j.agrformet.2022.109034 36003366 PMC9391928

[66] Suphioglu C, Singh MB, Taylor P, Knox RB, Bellomo R, Holmes P, Pu, R. Mechanism of grass-pollen-induced asthma. Lancet N. Am. Ed. 1992; 339(8793): 569–572. doi: 10.1016/0140-6736(92)90864-Y 1347092

[67] Schappi GF, Taylor PE, Staff IA, Rolland JM, Suphioglu C. Immunologic significance of respirable atmospheric starch granules containing major birch allergen Bet v 1. Allergy. 1999; 54: 478–483. doi: 10.1034/j.1398-9995.1999.00838.x 10380779

[68] Mercader J, Abtosway M, Baquedano E, Bird RW, Díez-Martín F, Domínguez-Rodrigo M, et al., Starch contamination landscapes in field archaeology: Olduvai Gorge, Tanzania. Boreas. 2017; 47 (4): 918–934. doi: 10.1111/bor.12241

[69] Barton H, Torrence R. Cooking up recipes for ancient starch: assessing current methodologies and looking to the future. J Archaeol Sci. 2015; 56: 194–201. doi: 10.1016/j.jas.2015.02.031

[70] Washburn DK, Washburn WN, Shipkova P, Pelleymounter MA. Chemical analysis of cacao residues in archaeological ceramics from North America: considerations of contamination, sample size and systematic controls. J Archaeol Sci. 2014; 50: 191–207. doi: 10.1016/j.jas.2014.07.011

[71] Babot MP. Tecnología y utilización de artefactos de molienda en el noroeste prehispánico. PhD tesis, Facultad de Ciencias Naturales e Instituto Miguel Lillo, Universidad Nacional de Tucumán, San Miguel de Tucumán, Argentina. 2004. Available from: https://www.academia.edu/6763539/Tesis_doctoral_Tecnolog%C3%ADa_y_utilizaci%C3%B3n_de_artefactos_de_molienda_en_el_Noroeste_Prehisp%C3%A1nico_Babot_M_P

[72] Babot MP. Granos de almidón en contextos arqueológicos: posibilidades y perspectivas a partir de casos del noroeste argentino. In: Marconetto B, Babot P, Oliszewski O. Editors. Paleoetnobotánica del cono sur: Estudios de caso y propuestas metodológicas. Córdoba: Ferreyra Editor. 2007. pp. 95–125.

[73] Field J. Reference collections. In: Torrence R, Barton H. Editors, Ancient Starch Research. Walnut Creek, California: Left Coast Press. 2006. pp. 95–113.

[74] Mellén-Blanco F. Manuscrito y documentos españoles para la historia de la Isla de Pascua. Madrid: Biblioteca CEHOPU. 1986.

[75] Torrence R, Wrig R, Conway R. Identification of starch granules using image analysis and multivariate techniques. J Archaeol Sci. 2004; 31:519–532. doi: 10.1016/j.jas.2003.09.014

[76] Bendjoudi H, Hubert P. Le coefficient de compacité de Gravelius: analyse critique d’un indice de forme des bassins versants, Hydrol J. 2002; 47(6): 921–930. doi: 10.1080/02626660209493000

[77] Almidones Pagán J. Guía de material comparativo moderno del Ecuador para los estudios paleoetnobotánicos en el neotrópico. Buenos Aires: Aspha Ediciones. 2015.

[78] Gareth J, Witten D, Hastie T, Tibshirani R. An introduction to statistical learning: with applications in R. New York: Springer. 2013.

[79] R Core Team. R: A language and environment for statistical computing. Vienna, Austria: R Foundation for Statistical Computing. 2021. https://www.R-project.org/

[80] James G, Witten D, Hastie T, Tibshirani R. An Introduction to Statistical Learning: with Applications in R. Springer Texts in Statistics. New York: Springer; 1st ed. 2013, Corr. 7th printing, Book 103. 2017.

[81] Sánchez D. Advanced support vector machines and kernel methods. Neurocomp. 2003; 55 (1–2): 5–20. doi: 10.1016/S0925-2312(03)00373-4

[82] Belgiu M, Drăguţ L. Random forest in remote sensing: A review of applications and future directions, ISPRS J Photogram and Remote Sensing. 2016; 114: 24–31. doi: 10.1016/j.isprsjprs.2016.01.011

[83] Kassambara A. Practical Guide to Cluster Analysis in R: Unsupervised Machine Learning (Vol. 1). STHDA. 2017.

[84] Leonel M. Análise da forma e tamanho de grânulos de amidos de diferentes fontes botánicas. Analysis of the shape and size of starch grains from different botanical species. Ciênc. Tecnol. Aliment., Campinas. 2007; 27(3): 579–588. doi: 10.1590/S0101-20612007000300024

[85] Arráiz H, Barbarin N, Pasturel M, Beaufort L, Domínguez-Rodrigo M, Barboni D. Starch granules identification and automatic classification based on an extended set of morphometric and optical measurements. J. Archaeol. Sci. Rep. 2016; 7: 169–172. doi: 10.1016/j.jasrep.2016.03.039

[86] Tulver K. The factorial structure of individual differences in visual perception. Consciousness & Cognit. 2019; 73:102762. doi: 10.1016/j.concog.2019.102762 31176848

[87] Barber IG, Higham TFG. Archaeological science meets Māori knowledge to model pre-Columbian sweet potato (*Ipomoea batatas*) dispersal to Polynesia’s southernmost habitable margins. PLoS ONE. 2021; 16(4): e0247643. doi: 10.1371/journal.pone.0247643 33852587 PMC8046222

[88] Coster ACF, Field JH. What starch grain is that? A geometric morphometric approach to determining plant species origin. J Archaeol Sci. 2015.58: 9–25. doi: 10.1016/j.jas.2015.03.01.4

[89] Field JH, Summerhayes GR, Luu S, Coster ACF, Ford A, Mandui H, et al. Functional studies of flaked and ground stone artefacts reveal starchy tree nut and root exploitation in mid-Holocene highland New Guinea. The Holocene. 2020; 30(9): 1360–1374. doi: 10.1177/09596836209199

[90] Hayes EH, Nango M, Field JH, Coster ACF, Fullagar R, Matheson C, et al. Holocene grinding stones at Madjedbebe reveal the processing of starchy plant taxa and animal tissue. J Archaeol Sci Rep. 2021; 35:102754. doi: 10.1016/j.jasrep.2020.102754

[91] Piperno DR, Ranere AJ, Holst I, Hansell P. Starch granules reveal early root crop horticulture in the Panamanian tropical forest. Nature 2000; 407: 894e897. doi: 10.1038/35038055 11057665

[92] Piperno DR, Weiss E, Holst I, Nadel D. Processing of wild cereal grasses in the Upper PAlaeolithic revealed by starch grain analysis. Nature 2004; 430: 670e673. doi: 10.1038/nature02734 15295598

[93] Leach H. Cooking without pots: aspects of prehistoric and traditional Polynesian cooking. NZ J Archaeol. 1982; 4:149–156.

[94] Prebble M, Anderson A, Kennett DJ. Forest clearance and agricultural expansion on Rapa, Austral Archipelago, French Polynesia. Holocene. 2013; 23:179–196. doi: 10.1177/0959683612455551

[95] Barton H, White P. Use of Stone, and Shell Artifacts at Balof 2, New Ireland, Papua New Guinea. Asian Perspectives. 1993. 32 (2): 169–181.

[96] Denham TP, Haberle SG, Lentfer C, Fullagar R, Field J, Therin M, et al. Origins of Agriculture at Kuk Swamp in the Highlands of New Guinea. Science. 2003; 301(5630): 189–193. doi: 10.1126/science.1085255 12817084

[97] Horrocks M, Shane PA, Barber IG, D’Costa DM, Nichol SL. Microbotanical remains reveal Polynesian agriculture and mixed cropping in early New Zealand. Rev Palaeobot & Palynol. 2004; 131(3–4):147–157. doi: 10.1016/j.revpalbo.2004.03.003

[98] Fullagar R, Field J, Denham T, Lentfer C. Early and mid Holocene tool-use and processing of taro (*Colocasia esculenta*), yam (*Dioscorea* sp.) and other plants at Kuk Swamp in the highlands of Papua New Guinea. J Archaeol Science. 2006; 33 (5): 595–614. doi: 10.1016/j.jas.2005.07.020

[99] Horrocks M, Nunn PD. Evidence for introduced taro (*Colocasia esculenta*) and lesser yam (*Dioscorea esculenta*) in Lapita-era (c. 3050e2500 cal. yr BP) deposits from Bourewa, southwest Viti Levu Island, Fiji. J Archaeol Sci. 2007; 34:739–748. doi: 10.1016/j.jas.2006.07.011

[100] Martinsson-Wallin H. Archaeological Excavation at Vinapu (Rapa Nui). Rapa Nui J. 2004; 18(1): 7–8.

[101] Martinsson-Wallin H. Vinapu area re-visited. In: Rull V. and Stevenson C.M. editors. The Prehistory of Easter Island (Rapa Nui): Towards an Integrative Interdisciplinary Framework. New York: Springer. 2022. Pp. 173–204. doi: 10.1007/978-3-030-91127-0

[102] Zerega NJC, Ragone D, Motley T. Complex origins of breadfruit (*Artocarpus altilis*, Moraceae): implications for human migrations in Oceania. Am J Bot. 2004; 91 (5):760–6. doi: 10.3732/ajb.91.5.760 21653430

[103] Bellwood P. First Islanders. Prehistory and Human Migration in Island Southeast Asia. New Jersey: Wiley-Blackwell. 2017.

[104] Kirch PV. On the road of the winds: an archaeological history of the Pacific Islands before European contact. Berkeley, California: University of California Press. 2000.

[105] Gibbons A. The peopling of the Pacific. Science 2001; 291:1735–1737. doi: 10.1126/science.291.5509.1735 11249818

[106] Métraux A. Ethnology of Easter Island. B.P. Bishop Museum Bulletin 160. Honolulu: Bishop Museum, 1971.

[107] Kahn JG, Ragone D. Identification of Carbonized Breadfruit (*Artocarpus altilis*) Skin: Refining Site Function and Site Specialization in the Society Islands, East Polynesia. J Ethnobiol. 2013; 33(2): 237–258. doi: 10.2993/0278-0771-33.2.237

[108] Kahn J, Coil J. What housepost tell us about status difference in Prehistoric Society: An Interpretation of Charcoal Analysis, Sacred Woods and Inter-site Variability. J Polyn Soc. 2006; 15 (4): 319–352.

[109] Huebert J M, Allen MS. Anthropogenic forests, arboriculture, and niche construction in the Marquesas Islands (Polynesia). J Anthrop Archaeol. 2020; 57:101122. doi: 10.1016/j.jaa.2019.101122

[110] Kirch PV, Molle G, Nickelsen C, Mills P, Dotte-Sarout E, Swift J, et al. Human ecodynamics in the Mangareva Islands: a stratified sequence from Nenega-Iti Rock Shelter (site AGA-3, Agakauitai Island). Archaeol in Oceania. 2015; 50 (1): 23–42. doi: 10.1002/arco.5050

[111] McCoy MD, Graves MW, Murakami G. Introduction of Breadfruit (*Artocarpus altilis*) to the Hawaiian Islands. Econ Bot. 2010; 64: 374–381. doi: 10.1007/s12231-010-9140-1

[112] Sáez A, Valero-Garcés B-L, Giralt S, Moreno A, Bao R, Pueyo JJ, et al. Glacial to Holocene climate changes in the SE Pacific. The Raraku Lake sedimentary record (Easter Island, 27°S), Quaternary Sci Rev, 2009; 28 (25–26): 2743–2759. doi: 10.1016/j.quascirev.2009.06.018

[113] Orliac C, Orliac M. Les structures de combustion et leur interprétation archéologique: quelques exemples en Polynésie. J Soc des Océanistes. 1980; 36:66–67.

[114] Bailey LH. Manual of cultivated plants. New York. London: Macmillan & Co., Ltd. 1924. pp. 202–203.

[115] Swahn JO. The lore of spices Their history, nature and uses around the world. Gothenburg, Sweden: AB Nordbok 1991.

[116] Munda S., Dutta S., Haldar S., Lal M. 2018. Chemical Analysis and Therapeutic Uses of Ginger (*Zingiber officinale* Rosc.) Essential Oil: A Review. Journal of Essential Oil-Bearing Plants 21(4): 994–1002. doi: 10.1080/0972060X.2018.1524794

[117] Wang W, Nguyen KTK, Zhao C, Hung H-C. Earliest curry in Southeast Asia and the global spice trade 2000 years ago. Sci Advan. 2023; 9 (29): eadh5517. doi: 10.1126/sciadv.adh5517 37478176 PMC10361603

[118] Wang J, Zhao X, Wang H, Liu L. Plant exploitation of the first farmers in northwest China: Microbotanical evidence from Dadiwan. Quat. Int. 2019; 529: 3–9. doi: 10.1016/j.quaint.2018.10.019

[119] Liu L, Wang J, Levin MJ, Sinnott-Armstrong N, Zhao H, Zhao Y, et al, The origins of specialized pottery and diverse alcohol fermentation techniques in Early Neolithic China. P Nat Acad Sci. 2019; 116 (26): 12767–12774. doi: 10.1073/pnas.1902668116 31160461 PMC6600912

[120] Larsson M, Foley B. The king’s spice cabinet–Plant remains from Gribshunden, a 15^th^ century royal shipwreck in the Baltic Sea. PLoS One. 2023;18(1): e0281010. doi: 10.1371/journal.pone.0281010 36701280 PMC9879437

[121] Kaushal M, Gupta A, Vaidya D, Gupta M. Postharvest Management and Value Addition of Ginger (Zingiber Officinale Roscoe): A Review. Inter J Environ, Agric & Biotech (IJEAB). 2017; 2(1): 397–412. doi: 10.22161/ijeab/2.1.50

[122] Lepofsky D. The Ethnobotany of Cultivated Plants of the Maohi of the Society Islands. Econ Bot. 2003; 57(1): 73–92. http://www.jstor.org/stable/4256644.

[123] Pauku RL. *Inocarpus fagifer* (Tahitian chestnut). In. Elevith CR. Editor. Species profiles for Pacific Island agroforestry: Ecological, economic, and cultural renewal. Hawaii: Permanent Agriculture Resources. 2006. ISBN: 0970254458.

[124] Pearsall DM. Plant Domestication and the Shift to Agriculture in the Andes. In: Silverman H, Isbell WH. editors. The Handbook of South American Archaeology. New York, NY: Springer, 2008. doi: 10.1007/978-0-387-74907-5_7

[125] Manioc Langdon R., a Long-Concealed Key to the Enigma of Easter Island. Geogr J. 1988; 154 (3): 324–336. doi: 10.2307/634606

[126] Ballard C, Brown P, Bourke RM, Harwood T. Editors. The sweet potato in Oceania: A reappraisal. Ethnology Monographs 19, Oceania Monograph 56. Sydney: University of Sydney, 2005.

[127] Scaglion R. Kumara in the Ecuadorian Gulf of Guayaquil? In: Ballard C, Brown P, Bourke RM, Harwood T. editors. The Sweet Potato in Oceania: A Reappraisal, New South Wales, Australia: University of Sydney Press. 2005. pp 35–42.

[128] Scaglion R, Cordero MA. Did ancient Americans reach the New World? Evaluating evidence from the Ecuadorian Gulf of Guayaquil. In: Jones TL, Storey AA, Matisoo Smith EA, Ramírez JM. Editors. Polynesians in America: Pre-Columbian Contacts with the New World. Plymouth: Altamira Press. 2011. pp.171–193.

[129] Barrau J. L’énigme de la patate douce en Océanie. Marseille: Etudes d’Outre-Mer. 1957; 40: 3–87.

[130] Yen DE. The Sweet Potato and Oceania: An Essay in Ethno-Botany. Honolulu, Hawaii: Bishop Museum Press. 1974.

[131] Green RC. Sweet potato transfers in Polynesian prehistory. In: Ballard C, Brown P, Bourke RM, Harwood T. Editors. The Sweet Potato in Oceania: A Reappraisal, Oceania Monograph 56. Sydney: University of Sydney. 2005. pp. 43–62.

[132] Niespolo EM, Sharp WD, Kirch PV. The dating of coral abraders from stratified deposits at Tangatatau Rockshelter, Mangaia, Cook Islands: Implications for building precise chronologies in Polynesia. J Archaeol Sci. 2019; 101:21–33. doi: 10.1016/j.jas.2018.11.001

[133] Ladefoged TN, Graves MW, Coil JH. The introduction of sweet potato in Polynesia: Early remains in Hawai’i. J Polyn Soc. 2005; 114 (4): 359–73. https://www.jstor.org/stable/20707306.

[134] Skjølsvold A. Site E-2 a circular stone dwelling, Anakena. In: Heyerdahl T, Ferdon E. editors. Reports of the Norwegian Expedition to Easter Island and the East Pacific, Vol. 1. Archaeology of Easter Island. Monographs of the School of American Research and the Museum of New Mexico, No. 24, Santa Fe: School of American Research. 1961.

[135] Smith CS. Radiocarbon dates from Easter Island. In: Heyerdahl T, Ferdon E. editors. Reports of the Norwegian Archaeological Expedition to Easter Island and the East Pacific. Vol. 1: Archaeology of Easter Island. Monographs of the School of American Research and the Museum of New Mexico, No. 24, Santa Fe: School of American Research. 1961. pp. 393–96.

[136] Wallin P, Stevenson C, Ladefoged T. Sweet potatoe production on Rapa Nui. In: Ballard C, Brown P, Bourke RM, Harwood T. editors. The sweet potato in Oceania: A reappraisal. Ethnology Monographs 19. Oceania Monograph 56. Sydney: University of Sydney. 2005. pp. 85–88.

[137] Dubois A, Lenne P, Nahoe E, Rauch M. Plantas de Rapa Nui. Guía ilustrada de la flora de interés ecológico y patrimonial. Umanga mo te Natura, Santiago, Chile: CONAF, ONF International. 2013.

[138] Cartay R. Difusión y comercio de la yuca (*Manihot esculenta*) en Venezuela y en el mundo. Agroalim [online]. 2004; 9(18): 13–22. Available at: https://ve.scielo.org/scielo.php?script=sci_arttext&pid=S1316-03542004000100001.

[139] Barrau J. Useful Plants of Tahiti. Paris: Société des Océanistes. Dossier 8. 1971.

[140] Sardos J, McKey D, Duval MF, Malapa R, Noyer JL, Lebot V. Evolution of cassava (*Manihot esculenta* Crantz) after recent introduction into a South Pacific Island system: the contribution of sex to the diversification of a clonally propagated crop. Genome. 2008; 51(11): 912–921. doi: 10.1139/g08-080 18956024

[141] Martínez E. Vocabulario de la Lengua Rapa-Nui. Santiago de Chile: Instituto Central Meteorológico y Geofísico de Chile. 1913.

[142] Fuentes F. Reseña botánica sobre la Isla de Pascua. Bol Mus Nac Chile. 1913; 5: 320–337.

[143] Muñoz D, Seelenfreund A, Fajreldin V. La antropología chilena en Rapa Nui: una retrospectiva. Rev Antropol del Sur. 2020; 7(14): 89–126. doi: 10.25074/rantros.v7i14.1889

[144] Blixen O. La expedición española de 1770 a la Isla de Pascua. Moana. 1977; 1(9):1–18.

[145] O’Hair SK, Maynard DN. Vegetables of tropical climates. Edible Aroids. In: Encyclopedia of Food Sciences and Nutrition (Second Edition), Cambridge, Massachusetts: Academic Press. 2003. pp. 5970–5973. doi: 10.1016/B0-12-227055-X/01245-1

[146] Manner HI. Farm and forestry production and marketing profile for Tannia (*Xanthosoma* spp.). In: Elevitch CR. editor. Specialty crops for Pacific Island Agroforestry. Holualoa, Hawaii, USA: Permanent Agriculture Resources (PAR). 2011. pp. 1–16.

[147] Govaerts R. World Checklist of Araceae. Richmond, UK: Royal Botanic Gardens, Kew. 2013. http://apps.kew.org/wcsp/.

[148] Giacometti D, Leó, J. La agricultura amazónica y caribeña. Yautía o malanga (*Xanthosoma sagittifolium*). In: Hernández Bermejo J.E, León J. (Eds.), Cultivos marginados otra perspectiva de 1492. Rome: FAO Published in collaboration with the Jardín Botánico de Córdoba, (España) as part of the Programa Etnobotánica 92 (Andalucía). 1994. Available from: http://www.fao.org/tempref/GI/Reserved/FTP_FaoRlc/old/prior/segalim/prodalim/prodveg/cdrom/contenido/libro09/home9.htm.

[149] PIER. Pacific Islands Ecosystems at Risk. Honolulu, Hawaii, USA: HEAR, University of Hawaii. 2013. http://www.hear.org/pier/index.html.

[150] Pollock N. These roots remain. Food habits in islands of the Central and Eastern Pacific since Western Contact. Laie, Hawaii: The Institute of Polynesian Studies. 1992.

[151] Ioannidis AG, Blanco-Portillo J, Sandoval K, Hagelberg E, Miquel-Poblete J., Moreno- Mayar J, Rodríguez-Rodríguez J, et al. Native American gene flow into Polynesia predating Easter Island settlement. Nature. 2020; 583, 572–577. doi: 10.1038/s41586-020-2487-2 32641827 PMC8939867

[152] Buck PH. Vikings of the Sunrise. New York: Frederick A Stokes Company. 1938.

[153] Dixon RB. Culture contact and migration versus independent origin: A plea for more light. Am Anthropol. 1918; 20: 124–8.

[154] Emory K. Oceanian influence on American Indian culture. Nordenskjold’s view. J Polynesian Soc. 1942; 51 (2): 126–35. https://www.jstor.org/stable/20702896.

[155] Heyerdahl T. American Indians in the Pacific: The Theory behind the Kon Tiki Expedition. London: Allen & Unwin. 1952.

[156] Green RC. A range of disciplines support a dual origin for the bottle gourd in the Pacific. J Polynesian Soc. 2000; 109(2): 191–97. www.jstor.org/stable/20706916.

[157] Green RC. Commentary on the sailing raft, the sweet potato and the South American connection. Rapa Nui J. 2001; 15(2): 69–77. https://kahualike.manoa.hawaii.edu/rnj/vol15/iss2/2.

[158] Jones TL, Klar KA. Diffusionism reconsidered: Linguistic and archaeological evidence for prehistoric Polynesian contact with Southern California. Am Antiquity. 2005; 70: 457–84. doi: 10.2307/40035309

[159] Klar KA, Jones TL. Linguistic evidence for a prehistoric Polynesia—Southern California contact event. Anthrop Linguist. 2005;47(4): 369–400. https://www.jstor.org/stable/25132351.

[160] Ramírez JM. Transpacific Contacts. Rapa Nui J. 1990; 4(4): 53–55.

[161] Ramírez JM. Contactos transpacíficos: Un acercamiento al problema de los supuestos rasgos polinésicos en la cultura mapuche. Clava. 1992; 5: 41–74.

[162] Jones TL. The artifact record from North America. In: Jones TL, Storey AA, Matisoo-Smith EA, Ramirez-Aliaga J M. editors. Polynesians in America. Pre-Columbian Contacts with the New World, Plymouth: Altamira Press. 2011. pp. 71–94.

[163] Matisoo-Smith E. Human biological evidence for Polynesian contacts with the Americas: Finding Maui on Mocha? In: Jones TL, Storey AA, Matisoo-Smith EA, Ramirez-Aliaga JM. Editors. Polynesians in America: Pre-Columbian contacts with the New World. Plymouth: Altamira Press. 2011. pp. 208–222.

[164] Matisoo-Smith E, Ramírez JM. Human skeletal evidence of Polynesian presence in South America. Metric analyses of six crania from Mocha Island, Chile. J Pac Archaeol. 2010; 1 (1): 76–88. https://pacificarchaeology.org/index.php/journal/article/view/11.

[165] Storey AA, Ramírez JM, Quiroz D, Burley DV, Addison DJ, Walter R, et al. Radiocarbon and DNA evidence for a pre-Columbian introduction of Polynesian chickens to Chile. P Natl Acad Sci. 2007; 104 (25): 10335–9. doi: 10.1073/pnas.0703993104 17556540 PMC1965514

[166] Storey AA, Quiróz D, Matisoo-Smith EA. A reappraisal of the evidence for pre-Columbian introduction of chickens to the Americas. In: Jones TL, Storey AA, Matisoo Smith EA, Ramírez JM. editors. Polynesians in America. Pre-Columbian contact with the New World, Plymouth: Altamira Press. 2011. pp. 139–170.

[167] Ramírez-Aliaga JM, Matisoo-Smith E. Polinesios en el sur de Chile en tiempos prehispánicos: evidencia dura, nuevas preguntas y una nueva hipótesis. (Polynesians of prehistoric times in Southern Chile: hard evidence, new questions and a new hypothesis). Clava. 2008; 7: 85–100.

