# Correction: Identification of breadfruit (*Artocarpus altilis*) and South American crops introduced during early settlement of Rapa Nui (Easter Island), as revealed through starch analysis

**DOI:** 10.1371/journal.pone.0317977

**Published:** 2025-02-12

**Authors:** Paloma Berenguer, Claudia Clavero, Mónica Saldarriaga-Córdoba, Antonio Rivera-Hutinel, Daniela Seelenfreund, Helene Martinsson-Wallin, Patricia Castañeda, Andrea Seelenfreund

The image data and artifact descriptions and measurements presented in Fig 3 and Table 1 were previously presented in [2]. Consequently, the figure legends of Fig 3 and Table 1 are updated as below.

**Fig 3:**
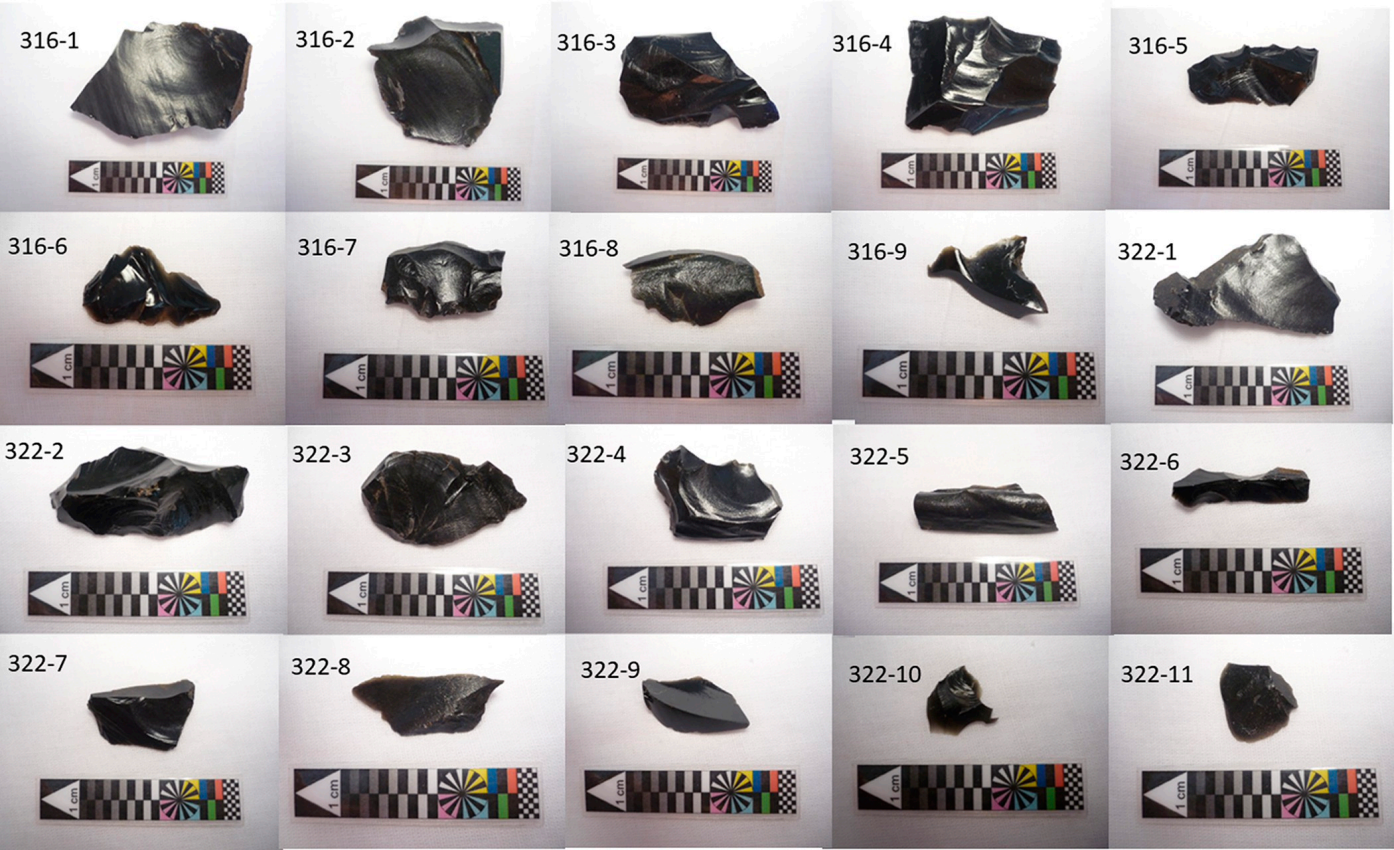
Artifacts sampled for this study. Image data were originally presented in [2].

**Table 1:** List of artifacts sampled for this study. Artifact descriptions and measurements were originally presented in [2].

Accession number	Museum (MAPSE) code	Collection	Original accession numbers*	Excavation unit	Artifact type	size (mm.)
0316–1	17-03-0316	03 Heyerdahl	A515-A516-A517	C1 (*bottom layer*)	flake	55.93 x 36.19 x 10.75
0316–2	17-03-0316	03 Heyerdahl	A515-A516-A518	C1 (*bottom layer*)	flake	49.63 x 43.43 x 9.27
0316–3	17-03-0316	03 Heyerdahl	A515-A516-A519	C1 (*bottom layer*)	flake	54.49 x 38.43 x 11.45
0316–4	17-03-0316	03 Heyerdahl	A515-A516-A520	C1 (*bottom layer*)	flake	47.44 x 34.14 x 11.02
0316–5	17-03-0316	03 Heyerdahl	A515-A516-A521	C1 (*bottom layer*)	flake	39.08 x 18.68 x 4.57
0316–6	17-03-0316	03 Heyerdahl	A515-A516-A522	C1 (*bottom layer*)	flake	32.37 x 19.35 x 3.85
0316–7	17-03-0316	03 Heyerdahl	A515-A516-A523	C1 (*bottom layer*)	flake	27.49 x 20.11 x 5.50
0316–8	17-03-0316	03 Heyerdahl	A515-A516-A524	C1 (*bottom layer*)	flake	31.93 x 18.26 x 3.86
0316–9	17-03-0316	03 Heyerdahl	A515-A516-A525	C1 (*bottom layer*)	flake	29.42 x 18.71 x 6.43
0322–1	17-03-0322	03 Heyerdahl	A515-A516-A517	C1 (*bottom layer*)	flake	52.04 x 25.52 x 7.91
0322–2	17-03-0322	03 Heyerdahl	A515-A516-A518	C1 (*bottom layer*)	flake	46.65 x 20.97 x 18.32
0322–3	17-03-0322	03 Heyerdahl	A515-A516-A519	C1 (*bottom layer*)	flake	40.31 x 22.02 x 7.21
0322–4	17-03-0322	03 Heyerdahl	A515-A516-A520	C1 (*bottom layer*)	flake	31.91 x 23.50 x 7.22
0322–5	17-03-0322	03 Heyerdahl	A515-A516-A521	C1 (*bottom layer*)	flake	37.07 x 13.01 x 10.18
0322–6	17-03-0322	03 Heyerdahl	A515-A516-A522	C1 (*bottom layer*)	flake	31.46 x 10.09 x 5.44
0322–7	17-03-0322	03 Heyerdahl	A515-A516-A523	C1 (*bottom layer*)	flake	28.41 x 18.13 x 4.94
0322–8	17-03-0322	03 Heyerdahl	A515-A516-A524	C1 (*bottom layer*)	flake	31.92 x 12.52 x 5.45
0322–9	17-03-0322	03 Heyerdahl	A515-A516-A525	C1 (*bottom layer*)	flake	27.06 14.46 x 6.46
0322–10	17-03-0322	03 Heyerdahl	A515-A516-A526	C1 (*bottom layer*)	flake	16.89 x 15.65 x 2.68
0322–11	17-03-0322	03 Heyerdahl	A515-A516-A527	C1 (*bottom layer*)	flake	19.14 x 17.12 x 4.00

In addition, the following section is added to the article:

**Acknowledgements**

The photographs, artifact descriptions, provenance data and measurements presented in Fig 3 and Table 1, and the preparation of the microscope slides of the archaeological residues were conducted by a former member of the research group (undergraduate student).
