# Supplementary figures and images for: Identification of breadfruit (Artocarpus altilis) and South American crops introduced during early settlement of Rapa Nui (Easter Island), as revealed through starch analysis
Source: PLoS One. 2024 Mar 20;19(3):e0298896. doi: 10.1371/journal.pone.0298896 (PMC10954183; doi:10.1371/journal.pone.0298896)

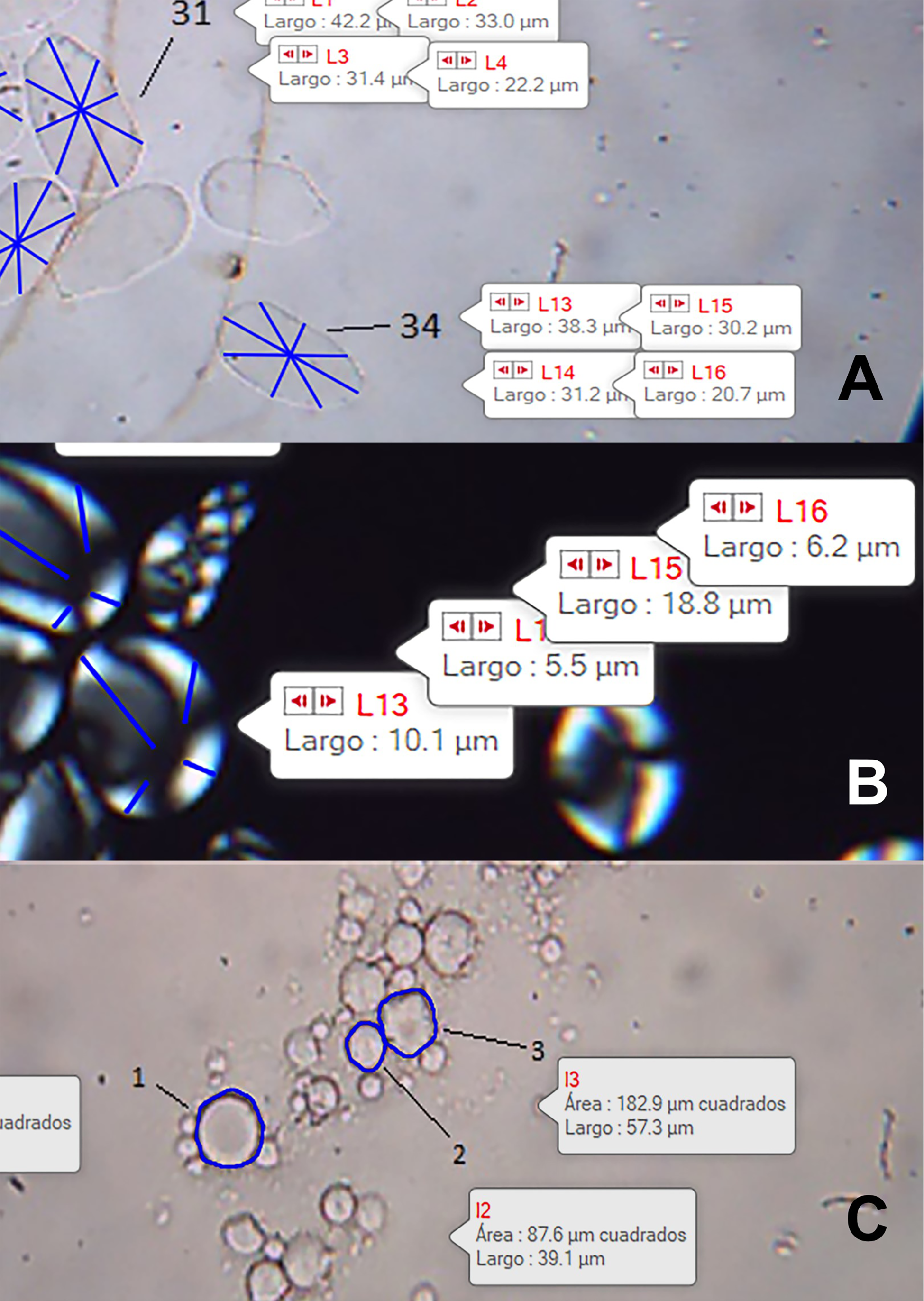

Supplement: S1 Fig — A. Maximum and minimum length of starch grains. B. Maximum and minimum distance of hilum to the rim of the grain. C. Perimeter and total area of the grain. (TIF) [file pone.0298896.s001.tif]

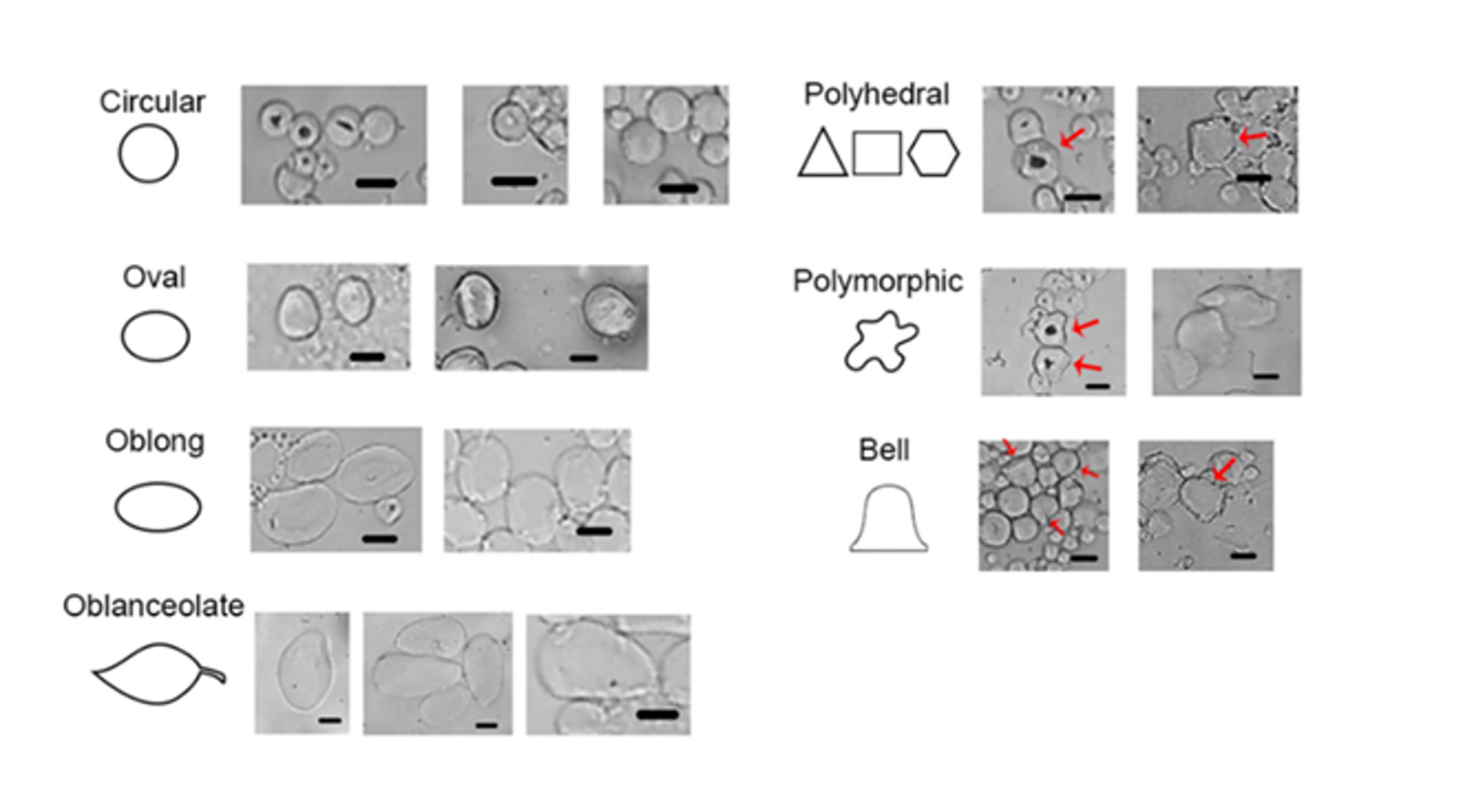

Supplement: S2 Fig — Scale bars correspond to 10 μm. (TIF) [file pone.0298896.s002.tif]

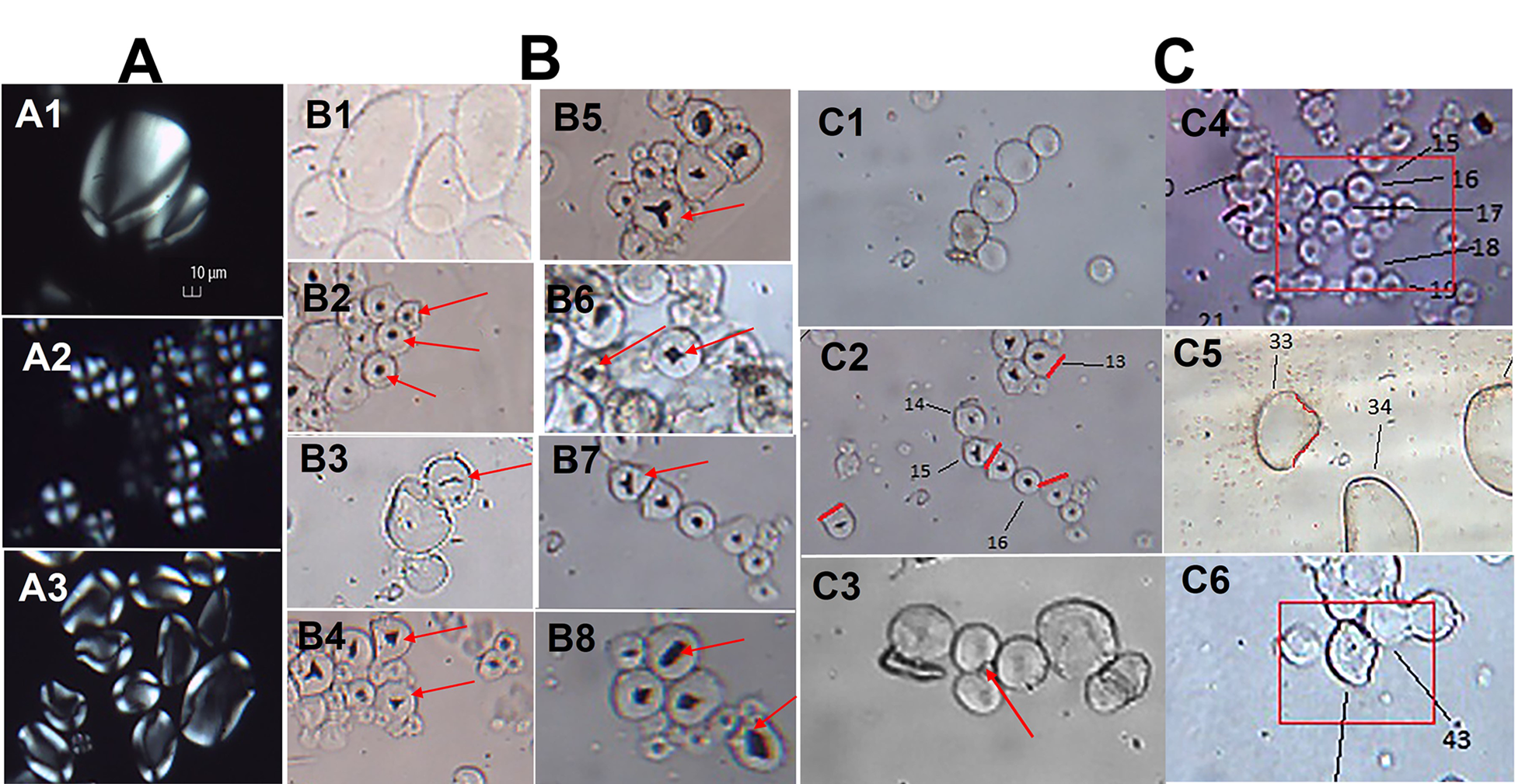

Supplement: S3 Fig — A. Style of the extinction cross, where A1 = curvy, A2 = straight, and A3 = wavy. B. Shape of the hilum fissure, in which B1 = absent, B2 = circular, B3 = simple, B4 = v shape, B5 = y shape, B6 square, B7 = hat shaped, and B8 = elongated. C. Shape of grain facettes, where C1 = absent, C2 = flat, C3 = Concave, C4 = multi-facetted, C5 = multi-concave, and C6 = Multi-mixed. (TIF) [file pone.0298896.s003.tif]

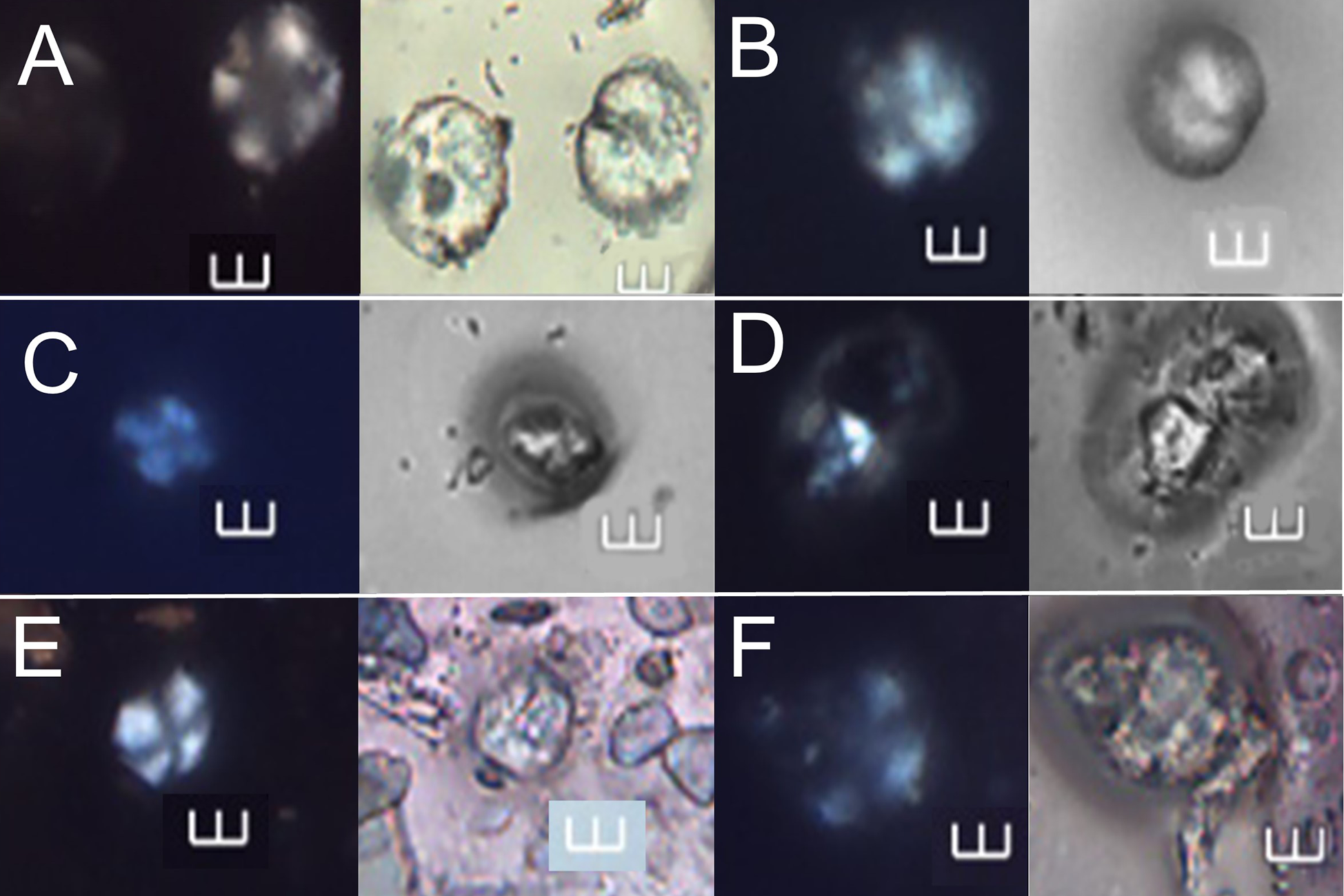

Supplement: S4 Fig — A- D Damaged grains, under polarized and brightfield light. E-F Starch grains found inside the nail polish on the edge of the slides. Scale bars = 10 μm. (TIF) [file pone.0298896.s004.tif]
