# Supplementary material for: Identification of breadfruit (Artocarpus altilis) and South American crops introduced during early settlement of Rapa Nui (Easter Island), as revealed through starch analysis
Source: PLoS One. 2024 Mar 20;19(3):e0298896. doi: 10.1371/journal.pone.0298896 (PMC10954183; doi:10.1371/journal.pone.0298896)
Supplement: S1 File — (DOCX) [file pone.0298896.s005.docx]

**Supplementary Tables**

**Tables 1 - 13: Statistical summary of the quantitative variables of the reference collection data set for each species considered in the reference collection**. Variables on all tables are identified by numbers: 1: Maximum length of the starch grain, 2: Minimum length of the starch grain, 3: Elongation index, 4: Total perimeter, 5: Total area, 6: Compactness Index, 7: Maximum distance between hilum and edge, 8: Minimum distance between hilum and edge, and 9: Centricity index.

**Table 1: *Artocarpus altilis***

| **Variables** | **1** | **2** | **3** | **4** | **5** | **6** | **7** | **8** | **9** |
| --- | --- | --- | --- | --- | --- | --- | --- | --- | --- |
| **N** | 200 | 200 | 200 | 200 | 200 | 200 | 200 | 200,00 | 200 |
| **Min** | 4,20 | 3,50 | 0,85 | 25,10 | 33,40 | 1,14 | 2,10 | 1,40 | 0,5 |
| **Max** | 12,90 | 11,00 | 1,69 | 45,80 | 102,00 | 1,47 | 5,10 | 3,90 | 0,67 |
| **Sum** | 1853,80 | 1584,00 | 235,32 | 7007,80 | 12887,60 | 248,01 | 661,10 | 511,90 | 112,95 |
| **Mean** | 9,27 | 7,92 | 1,18 | 35,04 | 64,44 | 1,24 | 3,31 | 2,56 | 0,56 |
| **Std. error** | 0,09 | 0,08 | 0,01 | 0,29 | 1,04 | 0,00 | 0,04 | 0,04 | 0,00 |
| **Variance** | 1,57 | 1,28 | 0,01 | 17,16 | 216,55 | 0,00 | 0,31 | 0,25 | 0,00 |
| **Stand. dev** | 1,25 | 1,13 | 0,11 | 4,14 | 14,72 | 0,06 | 0,55 | 0,50 | 0,03 |
| **Median** | 9,20 | 8,00 | 1,16 | 35,20 | 64,00 | 1,23 | 3,30 | 2,50 | 0,56 |
| **25 prcntil** | 8,50 | 7,10 | 1,10 | 32,00 | 52,75 | 1,19 | 2,90 | 2,20 | 0,54 |
| **75 prcntil** | 10,00 | 8,68 | 1,22 | 38,10 | 75,95 | 1,27 | 3,60 | 2,90 | 0,58 |
| **Mode** | 9,40 | 8,10 | NA | NA | NA | NA | 3,50 | 2,30 | 0,56 |
| **Skewness** | -0,16 | -0,10 | 1,39 | -0,04 | 0,18 | 0,83 | 0,49 | 0,24 | 0,66 |
| **Kurtosis** | 1,12 | 0,60 | 3,71 | -0,43 | -0,62 | 0,73 | 0,30 | -0,35 | 0,33 |
| **Geom. mean** | 9,18 | 7,84 | 1,17 | 34,79 | 62,73 | 1,24 | 3,26 | 2,51 | 0,56 |
| **Coeff. var** | 13,52 | 14,28 | 9,59 | 11,82 | 22,84 | 4,67 | 16,74 | 19,64 | 5,62 |

**Table 2: *Canna* sp*.***

| **Variables** | **1** | **2** | **3** | **4** | **5** | **6** | **7** | **8** | **9** |
| --- | --- | --- | --- | --- | --- | --- | --- | --- | --- |
| N | 100 | 100 | 100 | 100 | 100 | 100 | 100 | 100 | 100 |
| Min | 22,80 | 10,00 | 1,28 | 70,20 | 324,30 | 1,03 | 12,80 | 2,40 | 0,72 |
| Max | 71,60 | 46,10 | 3,15 | 251,70 | 3262,20 | 1,39 | 58,30 | 13,90 | 0,91 |
| Sum | 4469,70 | 2528,40 | 180,79 | 14333,30 | 119857,30 | 120,73 | 2953,30 | 508,60 | 84,94 |
| Mean | 44,70 | 25,28 | 1,81 | 143,33 | 1198,57 | 1,21 | 29,53 | 5,09 | 0,85 |
| Std. error | 1,11 | 0,72 | 0,03 | 3,61 | 60,03 | 0,00 | 0,93 | 0,20 | 0,00 |
| Variance | 124,31 | 52,25 | 0,09 | 1303,38 | 360376,40 | 0,00 | 85,98 | 3,89 | 0,00 |
| Stand. dev | 11,15 | 7,23 | 0,30 | 36,10 | 600,31 | 0,05 | 9,27 | 1,97 | 0,04 |
| Median | 45,20 | 24,95 | 1,79 | 146,20 | 1132,30 | 1,21 | 30,30 | 4,65 | 0,86 |
| 25 prcntil | 36,45 | 19,43 | 1,64 | 112,38 | 697,58 | 1,18 | 21,33 | 3,80 | 0,82 |
| 75 prcntil | 52,30 | 30,40 | 1,93 | 169,00 | 1550,80 | 1,24 | 36,80 | 5,88 | 0,89 |
| Mode | 45,70 | NA | NA | NA | NA | NA | NA | NA | 0,80 |
| Skewness | 0,16 | 0,37 | 1,39 | 0,24 | 0,80 | -0,06 | 0,18 | 1,70 | -0,70 |
| Kurtosis | -0,62 | -0,24 | 4,54 | -0,29 | 0,56 | 3,92 | -0,35 | 3,93 | -0,21 |
| Geom. mean | 43,28 | 24,25 | 1,79 | 138,72 | 1051,47 | 1,21 | 28,01 | 4,78 | 0,85 |
| Coeff. var | 24,94 | 28,59 | 16,72 | 25,19 | 50,09 | 3,73 | 31,40 | 38,76 | 5,08 |

**Table 3: *Colocasia esculenta***

| **Variables** | **1** | **2** | **3** | **4** | **5** | **6** | **7** | **8** | **9** |
| --- | --- | --- | --- | --- | --- | --- | --- | --- | --- |
| N | 200 | 200 | 200 | 200 | 200 | 200 | 200 | 200 | 200 |
| Min | 5,20 | 4,30 | 1,00 | 19,80 | 22,30 | 0,86 | 1,90 | 1,00 | 0,50 |
| Max | 10,80 | 8,50 | 1,51 | 42,10 | 81,50 | 2,13 | 4,60 | 3,10 | 1,00 |
| Sum | 1510,50 | 1281,50 | 236,30 | 5633,00 | 8862,10 | 240,96 | 562,40 | 420,10 | 114,90 |
| Mean | 7,55 | 6,41 | 1,18 | 28,17 | 44,31 | 1,20 | 2,81 | 2,10 | 0,57 |
| Std. error | 0,08 | 0,06 | 0,01 | 0,28 | 0,84 | 0,01 | 0,04 | 0,03 | 0,00 |
| Variance | 1,17 | 0,76 | 0,01 | 16,00 | 140,61 | 0,01 | 0,27 | 0,16 | 0,00 |
| Stand. dev | 1,08 | 0,87 | 0,10 | 4,00 | 11,86 | 0,09 | 0,52 | 0,40 | 0,04 |
| Median | 7,50 | 6,40 | 1,17 | 27,90 | 42,50 | 1,19 | 2,80 | 2,10 | 0,57 |
| 25 prcntil | 6,80 | 5,80 | 1,11 | 25,10 | 35,73 | 1,17 | 2,40 | 1,80 | 0,55 |
| 75 prcntil | 8,30 | 7,10 | 1,24 | 31,08 | 50,98 | 1,23 | 3,10 | 2,40 | 0,59 |
| Mode | NA | 6,50 | NA | NA | NA | NA | 2,90 | 1,80 | 0,57 |
| Skewness | 0,30 | 0,16 | 0,63 | 0,37 | 0,66 | 5,90 | 0,65 | 0,20 | 4,78 |
| Kurtosis | -0,12 | -0,58 | 0,48 | -0,03 | 0,25 | 63,83 | 0,30 | -0,48 | 44,04 |
| Geom. mean | 7,48 | 6,35 | 1,18 | 27,89 | 42,79 | 1,20 | 2,77 | 2,06 | 0,57 |
| Coeff. var | 14,30 | 13,64 | 8,11 | 14,20 | 26,76 | 7,27 | 18,46 | 18,87 | 7,62 |

**Table 4: *Curcuma longa***

| **Variables** | **1** | **2** | **3** | **4** | **5** | **6** | **7** | **8** | **9** |
| --- | --- | --- | --- | --- | --- | --- | --- | --- | --- |
| **N** | 100 | 100 | 100 | 100 | 100 | 100 | 100 | 100 | 100 |
| **Min** | 28,40 | 9,30 | 0,00 | 89,10 | 421,60 | 1,15 | 24,20 | 1,60 | 0,89 |
| **Max** | 52,10 | 20,40 | 4,23 | 156,00 | 1176,80 | 1,42 | 48,20 | 4,00 | 0,97 |
| **Sum** | 3884,60 | 1632,00 | 241,11 | 12041,30 | 72602,10 | 126,54 | 3405,30 | 230,30 | 93,61 |
| **Mean** | 39,24 | 16,32 | 2,41 | 120,41 | 726,02 | 1,27 | 34,05 | 2,30 | 0,94 |
| **Std. error** | 0,50 | 0,20 | 0,05 | 1,28 | 13,99 | 0,00 | 0,49 | 0,04 | 0,00 |
| **Variance** | 24,90 | 3,99 | 0,24 | 164,98 | 19560,19 | 0,00 | 23,87 | 0,18 | 0,00 |
| **Stand. dev** | 4,99 | 2,00 | 0,49 | 12,84 | 139,86 | 0,05 | 4,89 | 0,42 | 0,01 |
| **Median** | 38,50 | 16,45 | 2,33 | 117,70 | 704,65 | 1,26 | 33,35 | 2,30 | 0,94 |
| **25 prcntil** | 36,50 | 15,20 | 2,13 | 112,53 | 631,83 | 1,23 | 30,90 | 2,00 | 0,93 |
| **75 prcntil** | 41,80 | 17,90 | 2,63 | 127,40 | 803,98 | 1,29 | 36,75 | 2,50 | 0,94 |
| **Mode** | 34,80 | 15,70 | 2,17 | 115,80 | NA | NA | NA | NA | NA |
| **Skewness** | 0,47 | -0,61 | -0,30 | 0,70 | 0,75 | 0,62 | 0,52 | 0,98 | -0,52 |
| **Kurtosis** | 0,19 | 0,61 | 6,20 | 0,72 | 0,73 | 0,88 | 0,28 | 2,08 | 1,16 |
| **Geom. mean** | 38,93 | 16,19 | 0,00 | 119,76 | 713,31 | 1,26 | 33,71 | 2,27 | 0,94 |
| **Coeff. var** | 12,72 | 12,25 | 20,42 | 10,67 | 19,26 | 3,77 | 14,35 | 18,28 | 1,22 |

**Table 5: *Dioscorea alata***

| **Variables** | **1** | **2** | **3** | **4** | **5** | **6** | **7** | **8** | **9** |
| --- | --- | --- | --- | --- | --- | --- | --- | --- | --- |
| N | 100 | 100 | 100 | 100 | 100 | 100 | 100 | 100 | 100 |
| Min | 16,70 | 7,30 | 1,27 | 59,40 | 205,60 | 1,13 | 10,10 | 2,40 | 0,7 |
| Max | 45,70 | 24,10 | 4,30 | 135,60 | 964,30 | 1,31 | 34,80 | 7,50 | 0,9 |
| Sum | 2962,10 | 1435,60 | 212,04 | 9249,90 | 48450,80 | 119,86 | 2082,70 | 474,60 | 80,9 |
| Mean | 29,62 | 14,36 | 2,12 | 92,50 | 484,51 | 1,20 | 20,83 | 4,75 | 0,8 |
| Std. error | 0,53 | 0,29 | 0,05 | 1,50 | 15,24 | 0,00 | 0,50 | 0,11 | 0,0 |
| Variance | 28,58 | 8,52 | 0,24 | 224,34 | 23213,77 | 0,00 | 25,50 | 1,17 | 0,0 |
| Stand. dev | 5,35 | 2,92 | 0,49 | 14,98 | 152,36 | 0,04 | 5,05 | 1,08 | 0,0 |
| Median | 29,85 | 14,15 | 2,06 | 92,05 | 478,40 | 1,19 | 20,60 | 4,70 | 0,8 |
| 25 prcntil | 26,00 | 12,63 | 1,82 | 83,10 | 380,53 | 1,17 | 17,40 | 4,00 | 0,8 |
| 75 prcntil | 32,78 | 16,40 | 2,30 | 102,45 | 562,35 | 1,22 | 24,15 | 5,48 | 0,8 |
| Mode | NA | NA | NA | NA | 501,30 | NA | NA | 5,0 | NA |
| Skewness | 0,23 | 0,17 | 2,00 | 0,27 | 0,80 | 0,59 | 0,35 | 0,30 | -0,8 |
| Kurtosis | 0,39 | 0,62 | 6,60 | -0,01 | 0,90 | 0,16 | 0,24 | -0,09 | 1,0 |
| Geom. mean | 29,14 | 14,05 | 2,07 | 91,30 | 461,81 | 1,20 | 20,21 | 4,62 | 0,8 |
| Coeff. var | 18,05 | 20,33 | 23,11 | 16,19 | 31,45 | 3,04 | 24,25 | 22,81 | 6,0 |

**Table 6: *Inocarpus fagifer***

| **Variables** | **1** | **2** | **3** | **4** | **5** | **6** | **7** | **8** | **9** |
| --- | --- | --- | --- | --- | --- | --- | --- | --- | --- |
| N | 100 | 100 | 100,00 | 100,00 | 100,00 | 100,00 | 100,00 | 100,00 | 100,00 |
| Min | 8,2 | 7,70 | 1,02 | 27,60 | 52,70 | 1,06 | 2,60 | 2,10 | 0,40 |
| Max | 16,9 | 15,40 | 1,28 | 61,50 | 219,30 | 1,35 | 7,30 | 6,10 | 0,68 |
| Sum | 1166,9 | 1052,60 | 111,02 | 4078,50 | 10367,90 | 114,10 | 410,70 | 318,70 | 56,39 |
| Mean | 11,669 | 10,53 | 1,11 | 40,79 | 103,68 | 1,14 | 4,11 | 3,19 | 0,56 |
| Std. error | 0,181145 | 0,16 | 0,01 | 0,64 | 3,15 | 0,01 | 0,09 | 0,08 | 0,00 |
| Variance | 3,281353 | 2,68 | 0,00 | 41,49 | 989,23 | 0,00 | 0,84 | 0,61 | 0,00 |
| Stand. dev | 1,81145 | 1,64 | 0,06 | 6,44 | 31,45 | 0,05 | 0,92 | 0,78 | 0,04 |
| Median | 11,5 | 10,45 | 1,10 | 39,75 | 95,75 | 1,14 | 3,90 | 3,00 | 0,56 |
| 25 prcntil | 10,425 | 9,30 | 1,06 | 36,53 | 81,38 | 1,09 | 3,40 | 2,60 | 0,54 |
| 75 prcntil | 12,975 | 11,50 | 1,14 | 44,70 | 122,48 | 1,17 | 4,50 | 3,60 | 0,59 |
| Mode | 10,5 | NA | 1,06 | 43,5 | NA | NA | 3,9 | 2,7 | 0,55 |
| Skewness | 0,420348 | 0,50 | 0,88 | 0,60 | 1,12 | 1,00 | 1,10 | 1,14 | -0,20 |
| Kurtosis | 0,086427 | -0,13 | 0,22 | 0,49 | 1,97 | 1,57 | 1,40 | 2,07 | 3,15 |
| Geom. mean | 11,53198 | 10,40 | 1,11 | 40,30 | 99,41 | 1,14 | 4,02 | 3,10 | 0,56 |
| Coeff. var | 15,52361 | 15,54 | 5,67 | 15,79 | 30,34 | 4,68 | 22,29 | 24,51 | 6,68 |

**Table 7: *Ipomoea batatas***

| **Variables** | **1** | **2** | **3** | **4** | **5** | **6** | **7** | **8** | **9** |
| --- | --- | --- | --- | --- | --- | --- | --- | --- | --- |
| N | 200,00 | 200,00 | 200,00 | 200,00 | 200,00 | 200,00 | 200,00 | 200,00 | 200,00 |
| Min | 8,90 | 8,40 | 1,01 | 35,90 | 69,50 | 1,07 | 2,60 | 1,70 | 0,51 |
| Max | 26,50 | 22,70 | 1,45 | 120,90 | 468,70 | 2,12 | 11,60 | 7,40 | 0,77 |
| Sum | 3122,30 | 2768,00 | 226,37 | 11115,00 | 37293,10 | 233,30 | 1234,30 | 786,30 | 121,64 |
| Mean | 15,61 | 13,84 | 1,13 | 55,58 | 186,47 | 1,17 | 6,17 | 3,93 | 0,61 |
| Std. error | 0,22 | 0,20 | 0,01 | 0,83 | 4,94 | 0,01 | 0,13 | 0,08 | 0,00 |
| Variance | 9,59 | 7,77 | 0,01 | 136,95 | 4879,19 | 0,01 | 3,44 | 1,32 | 0,00 |
| Stand. dev | 3,10 | 2,79 | 0,08 | 11,70 | 69,85 | 0,08 | 1,86 | 1,15 | 0,06 |
| Median | 15,40 | 14,10 | 1,11 | 55,50 | 176,60 | 1,16 | 6,00 | 3,90 | 0,60 |
| 25 prcntil | 13,30 | 11,63 | 1,07 | 46,50 | 128,98 | 1,14 | 4,80 | 2,90 | 0,56 |
| 75 prcntil | 17,98 | 15,95 | 1,18 | 63,93 | 235,08 | 1,18 | 7,38 | 4,90 | 0,64 |
| Mode | NA | 14,6 | NA | NA | NA | NA | NA | 2,7 | 0,57 |
| Skewness | 0,27 | 0,12 | 1,20 | 0,96 | 0,61 | 9,63 | 0,43 | 0,23 | 0,56 |
| Kurtosis | -0,03 | -0,50 | 1,66 | 3,65 | 0,39 | 119,31 | -0,20 | -0,79 | -0,21 |
| Geom. mean | 15,30 | 13,55 | 1,13 | 54,41 | 173,58 | 1,16 | 5,89 | 3,76 | 0,61 |
| Coeff. var | 19,83 | 20,14 | 7,19 | 21,06 | 37,46 | 6,57 | 30,06 | 29,18 | 9,42 |

**Table 8: *Manihot esculenta***

| **Variables** | **1** | **2** | **3** | **4** | **5** | **6** | **7** | **8** | **9** |
| --- | --- | --- | --- | --- | --- | --- | --- | --- | --- |
| N | 100,00 | 100,00 | 100,00 | 100,00 | 100,00 | 100,00 | 100,00 | 100,00 | 100,00 |
| Min | 9,30 | 8,70 | 1,01 | 34,80 | 70,00 | 1,12 | 2,80 | 2,10 | 0,51 |
| Max | 22,30 | 19,10 | 1,30 | 76,40 | 335,10 | 1,68 | 9,40 | 7,60 | 0,64 |
| Sum | 1380,20 | 1239,20 | 111,34 | 5021,00 | 14480,10 | 118,98 | 514,90 | 405,00 | 56,06 |
| Mean | 13,80 | 12,39 | 1,11 | 50,21 | 144,80 | 1,19 | 5,15 | 4,05 | 0,56 |
| Std. error | 0,23 | 0,19 | 0,01 | 0,82 | 4,53 | 0,01 | 0,12 | 0,10 | 0,00 |
| Variance | 5,24 | 3,74 | 0,00 | 66,89 | 2053,18 | 0,00 | 1,41 | 1,03 | 0,00 |
| Stand. dev | 2,29 | 1,93 | 0,05 | 8,18 | 45,31 | 0,06 | 1,19 | 1,01 | 0,03 |
| Median | 13,70 | 12,25 | 1,11 | 49,45 | 137,25 | 1,18 | 5,10 | 3,90 | 0,56 |
| 25 prcntil | 12,10 | 11,10 | 1,07 | 44,78 | 116,30 | 1,16 | 4,45 | 3,40 | 0,54 |
| 75 prcntil | 15,38 | 13,78 | 1,15 | 55,15 | 170,88 | 1,19 | 5,88 | 4,70 | 0,58 |
| Mode | 13,70 | 13,80 | 1,05 | NA | NA | NA | 4,60 | 3,70 | 0,55 |
| Skewness | 0,50 | 0,39 | 0,85 | 0,45 | 1,01 | 5,09 | 0,59 | 0,64 | 0,39 |
| Kurtosis | 0,78 | 0,40 | 1,14 | 0,33 | 2,12 | 35,99 | 1,27 | 0,77 | -0,33 |
| Geom. mean | 13,62 | 12,24 | 1,11 | 49,56 | 138,26 | 1,19 | 5,02 | 3,93 | 0,56 |
| Coeff. var | 16,59 | 15,61 | 4,86 | 16,29 | 31,29 | 5,38 | 23,03 | 25,06 | 4,95 |

**Table 9: *Musa* sp**.

| **Variables** | **1** | **2** | **3** | **4** | **5** | **6** | **7** | **8** | **9** |
| --- | --- | --- | --- | --- | --- | --- | --- | --- | --- |
| N | 100,00 | 100,00 | 100,00 | 100,00 | 100,00 | 100,00 | 100,00 | 100,00 | 100,00 |
| Min | 24,70 | 10,30 | 1,16 | 78,70 | 294,00 | 1,14 | 15,60 | 1,50 | 0,80 |
| Max | 64,00 | 52,20 | 3,74 | 202,90 | 2357,50 | 1,54 | 56,70 | 9,60 | 0,96 |
| Sum | 4517,30 | 2229,10 | 214,90 | 13084,30 | 88269,80 | 126,56 | 3614,80 | 358,50 | 90,88 |
| Mean | 45,17 | 22,29 | 2,15 | 130,84 | 882,70 | 1,27 | 36,15 | 3,59 | 0,91 |
| Std. error | 0,80 | 0,63 | 0,06 | 2,15 | 31,64 | 0,01 | 0,87 | 0,16 | 0,00 |
| Variance | 63,59 | 39,43 | 0,37 | 461,84 | 100133,20 | 0,01 | 75,18 | 2,67 | 0,00 |
| Stand. dev | 7,97 | 6,28 | 0,60 | 21,49 | 316,44 | 0,09 | 8,67 | 1,63 | 0,03 |
| Median | 45,05 | 21,80 | 2,07 | 129,55 | 824,85 | 1,25 | 36,20 | 3,15 | 0,92 |
| 25 prcntil | 40,10 | 17,53 | 1,68 | 115,45 | 668,05 | 1,21 | 29,23 | 2,53 | 0,90 |
| 75 prcntil | 50,80 | 26,20 | 2,37 | 144,68 | 1062,48 | 1,29 | 42,80 | 4,08 | 0,93 |
| Mode | 48,5 | 23 | 2 | NA | NA | NA | 43,1 | 2,7 | NA |
| Skewness | 0,05 | 1,12 | 0,80 | 0,23 | 1,23 | 1,22 | 0,02 | 1,94 | -1,32 |
| Kurtosis | -0,14 | 3,85 | 0,22 | 0,58 | 3,79 | 1,25 | -0,47 | 4,34 | 1,44 |
| Geom. mean | 44,45 | 21,47 | 2,07 | 129,07 | 830,62 | 1,26 | 35,05 | 3,31 | 0,91 |
| Coeff. var | 17,65 | 28,17 | 28,15 | 16,42 | 35,85 | 6,75 | 23,99 | 45,54 | 3,79 |

**Table 10: *Spondias dulcis***

| **Variables** | **1** | **2** | **3** | **4** | **5** | **6** | **7** | **8** | **9** |
| --- | --- | --- | --- | --- | --- | --- | --- | --- | --- |
| N | 100 | 100 | 100 | 100 | 100 | 100 | 100 | 100 | 100 |
| Min | 9,90 | 9,60 | 1,03 | 36,10 | 77,10 | 1,07 | 2,40 | 1,80 | 0,54 |
| Max | 24,10 | 19,70 | 1,49 | 73,50 | 362,70 | 1,22 | 11,50 | 6,60 | 0,78 |
| Sum | 1748,20 | 1497,80 | 116,81 | 5882,70 | 21674,70 | 114,03 | 683,00 | 369,80 | 64,64 |
| Mean | 17,48 | 14,98 | 1,17 | 58,83 | 216,75 | 1,14 | 6,83 | 3,70 | 0,65 |
| Std. error | 0,27 | 0,21 | 0,01 | 0,80 | 6,11 | 0,00 | 0,18 | 0,10 | 0,01 |
| Variance | 7,30 | 4,52 | 0,01 | 63,53 | 3728,23 | 0,00 | 3,35 | 1,07 | 0,00 |
| Stand. dev | 2,70 | 2,13 | 0,09 | 7,97 | 61,06 | 0,04 | 1,83 | 1,03 | 0,06 |
| Median | 17,30 | 15,10 | 1,16 | 59,60 | 210,45 | 1,14 | 6,70 | 3,70 | 0,65 |
| 25 prcntil | 15,65 | 13,50 | 1,10 | 52,63 | 171,50 | 1,10 | 5,43 | 3,00 | 0,58 |
| 75 prcntil | 19,30 | 16,30 | 1,23 | 64,28 | 254,03 | 1,17 | 8,20 | 4,20 | 0,70 |
| Mode | 19,30 | NA | 1,04 | NA | NA | NA | 6,90 | NA | 0,56 |
| Skewness | 0,04 | -0,04 | 0,72 | -0,30 | 0,26 | -0,09 | 0,23 | 0,38 | 0,02 |
| Kurtosis | 0,00 | -0,17 | 0,81 | -0,19 | -0,37 | -1,28 | -0,20 | 0,13 | -1,03 |
| Geom. mean | 17,27 | 14,82 | 1,16 | 58,27 | 207,88 | 1,14 | 6,57 | 3,55 | 0,64 |
| Coeff. var | 15,45 | 14,20 | 7,50 | 13,55 | 28,17 | 3,51 | 26,81 | 27,93 | 9,96 |

**Table 11: *Triticum estivicum***

| **Variables** | **1** | **2** | **3** | **4** | **5** | **6** | **7** | **8** | **9** |
| --- | --- | --- | --- | --- | --- | --- | --- | --- | --- |
| N | 100 | 100 | 100 | 100 | 100 | 100 | 100 | 100 | 100 |
| Min | 14,70 | 12,60 | 1,01 | 54,40 | 20,80 | 1,08 | 5,60 | 4,20 | 0,48 |
| Max | 34,50 | 32,70 | 1,42 | 126,30 | 862,20 | 3,67 | 16,00 | 12,90 | 1,00 |
| Sum | 2471,00 | 2155,60 | 115,26 | 8913,60 | 45575,40 | 121,50 | 1009,10 | 785,90 | 56,77 |
| Mean | 24,71 | 21,56 | 1,15 | 89,14 | 455,75 | 1,21 | 10,09 | 7,86 | 0,57 |
| Std. error | 0,39 | 0,38 | 0,01 | 1,42 | 14,61 | 0,03 | 0,21 | 0,19 | 0,01 |
| Variance | 14,94 | 14,24 | 0,01 | 202,75 | 21339,12 | 0,07 | 4,62 | 3,63 | 0,00 |
| Stand. dev | 3,86 | 3,77 | 0,09 | 14,24 | 146,08 | 0,26 | 2,15 | 1,91 | 0,05 |
| Median | 25,10 | 21,55 | 1,15 | 89,35 | 439,55 | 1,20 | 9,75 | 7,70 | 0,56 |
| 25 prcntil | 21,60 | 18,60 | 1,09 | 79,15 | 364,00 | 1,15 | 8,53 | 6,43 | 0,54 |
| 75 prcntil | 27,08 | 24,00 | 1,20 | 98,58 | 532,45 | 1,23 | 11,55 | 8,98 | 0,59 |
| Mode | 26,1 | NA | NA | NA | NA | NA | 10,1 | 8,2 | NA |
| Skewness | -0,01 | 0,34 | 0,62 | 0,18 | 0,37 | 9,06 | 0,48 | 0,53 | 5,24 |
| Kurtosis | -0,03 | 0,24 | 0,33 | -0,02 | 0,66 | 87,51 | -0,22 | -0,11 | 40,48 |
| Geom. mean | 24,40 | 21,23 | 1,15 | 88,00 | 426,23 | 1,20 | 9,87 | 7,64 | 0,57 |
| Coeff. var | 15,64 | 17,51 | 7,61 | 15,97 | 32,05 | 21,05 | 21,30 | 24,24 | 9,56 |

**Table 12: *Xanthosoma* sp**.

| **Variables** | **1** | **2** | **3** | **4** | **5** | **6** | **7** | **8** | **9** |
| --- | --- | --- | --- | --- | --- | --- | --- | --- | --- |
| N | 98 | 98 | 98 | 98 | 98 | 98 | 98 | 98 | 98 |
| Min | 11,70 | 10,50 | 1,02 | 39,20 | 100,30 | 1,08 | 2,70 | 2,80 | 0,36 |
| Max | 23,50 | 20,00 | 1,38 | 78,10 | 345,30 | 1,44 | 9,90 | 7,60 | 0,66 |
| Sum | 1639,10 | 1435,30 | 112,08 | 5608,70 | 19499,00 | 113,94 | 556,10 | 442,90 | 54,29 |
| Mean | 16,73 | 14,65 | 1,14 | 57,23 | 198,97 | 1,16 | 5,67 | 4,52 | 0,55 |
| Std. error | 0,27 | 0,23 | 0,01 | 0,91 | 5,93 | 0,01 | 0,13 | 0,09 | 0,00 |
| Variance | 7,37 | 5,36 | 0,01 | 80,89 | 3448,37 | 0,00 | 1,74 | 0,88 | 0,00 |
| Stand. dev | 2,71 | 2,31 | 0,08 | 8,99 | 58,72 | 0,06 | 1,32 | 0,94 | 0,03 |
| Median | 16,55 | 14,45 | 1,13 | 57,25 | 188,00 | 1,15 | 5,50 | 4,40 | 0,55 |
| 25 prcntil | 14,90 | 12,98 | 1,09 | 50,95 | 161,88 | 1,12 | 4,90 | 3,90 | 0,54 |
| 75 prcntil | 18,53 | 16,30 | 1,18 | 64,90 | 241,93 | 1,19 | 6,53 | 5,03 | 0,57 |
| Mode | 18,00 | 14,30 | NA | 64,90 | NA | 1,12 | NA | 4,60 | 0,56 |
| Skewness | 0,08 | 0,18 | 1,03 | -0,01 | 0,34 | 1,55 | 0,51 | 0,76 | -1,64 |
| Kurtosis | -0,64 | -0,58 | 0,83 | -0,72 | -0,51 | 4,20 | 0,44 | 0,91 | 14,15 |
| Geom. mean | 16,50 | 14,46 | 1,14 | 56,52 | 190,29 | 1,16 | 5,52 | 4,43 | 0,55 |
| Coeff. var | 16,23 | 15,80 | 6,95 | 15,71 | 29,51 | 5,20 | 23,28 | 20,76 | 5,77 |

**Table 13:** ***Zingiber officinale***

| **Variables** | **1** | **2** | **3** | **4** | **5** | **6** | **7** | **8** | **9** |
| --- | --- | --- | --- | --- | --- | --- | --- | --- | --- |
| N | 100 | 100 | 100 | 100 | 100 | 100 | 100 | 100 | 100 |
| Min | 14,90 | 11,30 | 1,08 | 51,60 | 164,30 | 1,06 | 9,80 | 1,40 | 0,80 |
| Max | 35,70 | 20,60 | 2,02 | 108,30 | 580,30 | 1,27 | 29,40 | 3,80 | 0,93 |
| Sum | 2226,20 | 1562,50 | 143,06 | 7087,90 | 30847,60 | 114,80 | 1614,10 | 224,50 | 87,50 |
| Mean | 22,26 | 15,63 | 1,43 | 70,88 | 308,48 | 1,15 | 16,14 | 2,25 | 0,87 |
| Std. error | 0,38 | 0,22 | 0,02 | 1,08 | 8,78 | 0,00 | 0,38 | 0,05 | 0,00 |
| Variance | 14,46 | 4,77 | 0,03 | 116,08 | 7716,03 | 0,00 | 14,54 | 0,21 | 0,00 |
| Stand. dev | 3,80 | 2,18 | 0,19 | 10,77 | 87,84 | 0,04 | 3,81 | 0,46 | 0,03 |
| Median | 21,95 | 15,70 | 1,42 | 70,60 | 296,35 | 1,15 | 15,85 | 2,20 | 0,88 |
| 25 prcntil | 19,90 | 14,00 | 1,31 | 63,68 | 251,53 | 1,12 | 13,53 | 1,90 | 0,86 |
| 75 prcntil | 24,28 | 17,28 | 1,53 | 77,25 | 359,50 | 1,18 | 17,90 | 2,50 | 0,89 |
| Mode | NA | 14,60 | NA | NA | 276,50 | NA | 16,6 | 2 | NA |
| Skewness | 0,89 | -0,02 | 0,87 | 0,68 | 0,84 | 0,08 | 0,97 | 0,71 | -0,52 |
| Kurtosis | 1,60 | -0,61 | 0,88 | 0,97 | 0,76 | -0,14 | 1,53 | 1,06 | 0,18 |
| Geom. mean | 21,96 | 15,47 | 1,42 | 70,10 | 296,84 | 1,15 | 15,73 | 2,20 | 0,87 |
| Coeff. var | 17,08 | 13,98 | 12,97 | 15,20 | 28,48 | 3,30 | 23,63 | 20,27 | 2,95 |

**Table 14: Quantitative measurements and categorization of qualitative variables, taken on each archaeological starch grain.**

| **Archaeol. starch grain code** | **Maximum grain length** | **Minimum grain length** | **Elongation Index (Ie)** | **Total perimeter** | **Total area** | **Compactnes Index (Ic)** | **Maximum distance between hilum and border** | **Minimum distance between hilum and border** | **Centricity Index (Ice)** | **2D Shape** | **Hilum type** | **Hilum Fissure** | **Shape of hilum fissure** | **Facets** | **Style of extinction cross** |
| --- | --- | --- | --- | --- | --- | --- | --- | --- | --- | --- | --- | --- | --- | --- | --- |
| Ala 0002 | 18,8 | 14,8 | 1,3 | 61,2 | 219,2 | 1,2 | 6,6 | 5,7 | 0,5 | 1 | 1 | 0 | 0 | 0 | 0 |
| Ala 0004 | 13 | 11,6 | 1,1 | 46,6 | 130,1 | 1,2 | 5,6 | 3,6 | 0,6 | 1 | 1 | 1 | 5 | 0 | 0 |
| Ala 0005 | 6,8 | 6,1 | 1,1 | 21,8 | 29,4 | 1,1 | 3,6 | 2 | 0,6 | 0 | 0 | 0 | 0 | 0 | 0 |
| Ala 0006 | 22,2 | 17,7 | 1,3 | 77,8 | 347,3 | 1,2 | 11,3 | 10 | 0,5 | 6 | 1 | 0 | 0 | 1 | 2 |
| Ala 0007 | 15 | 8,6 | 1,7 | 43,9 | 114,7 | 1,2 | 6,7 | 6,1 | 0,5 | 4 | 1 | 0 | 0 | 3 | 0 |
| Ala 0010 | 15,6 | 14,6 | 1,1 | 53,7 | 189,7 | 1,1 | 6,6 | 5,9 | 0,5 | 1 | 1 | 0 | 0 | 2 | 0 |
| Ala 0011 | 17,8 | 15,1 | 1,2 | 59,7 | 236,2 | 1,1 | 6,4 | 5,6 | 0,5 | 1 | 1 | 1 | 1 | 0 | 0 |
| Ala 0012 | 16,9 | 15,4 | 1,1 | 58,2 | 204,5 | 1,1 | 7,6 | 7 | 0,5 | 6 | 1 | 1 | 2 | 3 | 1 |
| Ala 0013 | 20,8 | 18,3 | 1,1 | 72,8 | 312,9 | 1,2 | 7,5 | 6,2 | 0,5 | 6 | 1 | 1 | 7 | 1 | 1 |
| Ala 0014 | 8,6 | 5,6 | 1,5 | 26,4 | 38,5 | 1,2 | 3,9 | 2,1 | 0,7 | 2 | 1 | 0 | 0 | 0 | 1 |
| Ala 0015 | 33,2 | 30,2 | 1,1 | 111,3 | 789,5 | 1,1 | 12,2 | 9,3 | 0,6 | 1 | 1 | 0 | 0 | 0 | 0 |
| Ala 0016 | 23,7 | 21 | 1,1 | 76,3 | 374,3 | 1,1 | 7,3 | 5,8 | 0,6 | 1 | 1 | 1 | 2 | 0 | 0 |
| Ala 0021 | 15,6 | 12,5 | 1,2 | 51,6 | 148,9 | 1,2 | 6,1 | 5,2 | 0,5 | 6 | 1 | 1 | 8 | 1 | 1 |
| Ala 0023 | 15 | 13 | 1,2 | 49,1 | 157,3 | 1,1 | 5,8 | 4,1 | 0,6 | 1 | 1 | 1 | 7 | 0 | 1 |
| Ala 0024 | 11,9 | 10,5 | 1,1 | 39,1 | 97,3 | 1,1 | 4,6 | 3,4 | 0,6 | 3 | 2 | 0 | 0 | 0 | 0 |
| Ala 0027 | 13,8 | 11,9 | 1,2 | 45,1 | 131,9 | 1,1 | 5,9 | 4,8 | 0,6 | 1 | 2 | 0 | 0 | 0 | 0 |
| Ala 0037 | 14,4 | 12,9 | 1,1 | 46,6 | 143 | 1,1 | 8,2 | 7 | 0,5 | 0 | 1 | 3 | 0 | 0 | 0 |
| Ala 0039 | 16,9 | 15,2 | 1,1 | 57,7 | 209,2 | 1,1 | 8,3 | 7 | 0,5 | 6 | 2 | 0 | 0 | 5 | 0 |
| Ala 0041 | 22,8 | 20 | 1,1 | 72,4 | 351,1 | 1,1 | 6,9 | 5 | 0,6 | 2 | 1 | 0 | 0 | 0 | 0 |
| Ala 0043 | 9,5 | 7,7 | 0,7 | 33,4 | 69,9 | 1,1 | 4,1 | 3,5 | 0,5 | 5 | 1 | 0 | 0 | 3 | 0 |
| Ala 0044 | 15,2 | 14,8 | 1,1 | 53,7 | 191 | 1,1 | 5 | 3,4 | 0,6 | 1 | 1 | 0 | 0 | 0 | 0 |
| Ala 0045 | 15,3 | 10,8 | 1,1 | 47,1 | 131,6 | 1,2 | 5,1 | 3,9 | 0,6 | 2 | 1 | 0 | 0 | 5 | 2 |
| Ala 0046 | 15,5 | 8,1 | 1,1 | 49,5 | 126,7 | 1,2 | 8 | 4,5 | 0,6 | 3 | 1 | 1 | 2 | 2 | 1 |
| Ala 0047 | 12,9 | 11,7 | 1,0 | 45,1 | 125,8 | 1,1 | 6,5 | 4 | 0,6 | 6 | 1 | 1 | 2 | 1 | 1 |
| Ala 0048 | 11 | 9,7 | 0,8 | 37,2 | 91,6 | 1,1 | 4,9 | 4,3 | 0,5 | 6 | 2 | 0 | 0 | 1 | 1 |
| Ala 0049 | 17 | 15,5 | 1,3 | 58,4 | 214,3 | 1,1 | 8 | 7 | 0,5 | 6 | 1 | 0 | 0 | 5 | 0 |
| Ala 0050 | 17 | 15,4 | 1,3 | 56,3 | 187,9 | 1,2 | 6,8 | 6,2 | 0,5 | 6 | 1 | 0 | 0 | 5 | 1 |
| Ala 0051 | 16,4 | 15 | 1,2 | 57,3 | 204,6 | 1,1 | 5,5 | 3,8 | 0,6 | 6 | 1 | 0 | 0 | 3 | 1 |
| Ala 0052 | 10,5 | 8,1 | 0,8 | 40,8 | 101,4 | 1,1 | 2,1 | 3,4 | 0,4 | 4 | 0 | 0 | 0 | 3 | 0 |
| Ala 0053 | 11,8 | 9,2 | 0,9 | 48,5 | 146,7 | 1,1 | 5,4 | 3,6 | 0,6 | 6 | 0 | 0 | 0 | 0 | 0 |
| Ala 0055 | 16,5 | 13,5 | 1,2 | 55 | 198,8 | 1,1 | 7,5 | 5,6 | 0,6 | 1 | 2 | 1 | 2 | 0 | 0 |
| Ala 0056 | 10,5 | 8,9 | 0,8 | 36,4 | 79,8 | 1,1 | 4,4 | 3,6 | 0,6 | 1 | 1 | 0 | 0 | 3 | 0 |
| Ala 0057 | 10,3 | 9,3 | 0,7 | 33,7 | 73,2 | 1,1 | 3,4 | 3,7 | 0,5 | 1 | 2 | 0 | 0 | 1 | 0 |
| Ala 0058 | 19,5 | 15,1 | 1,4 | 61,8 | 234,3 | 1,1 | 6,9 | 5,2 | 0,6 | 6 | 1 | 1 | 2 | 1 | 1 |
| Ala 0060 | 14,8 | 12,3 | 1,1 | 49,5 | 141,8 | 1,2 | 6,1 | 4,1 | 0,6 | 4 | 1 | 1 | 4 | 3 | 0 |
| Ala 0061 | 20,3 | 18,8 | 1,5 | 74,1 | 312,6 | 1,2 | 9 | 6,6 | 0,6 | 6 | 1 | 1 | 7 | 3 | 1 |
| Ala 0063 | 16,7 | 14,1 | 1,2 | 54,2 | 173,8 | 1,2 | 6,2 | 5,1 | 0,5 | 6 | 1 | 1 | 3 | 1 | 1 |
| Ala 0064 | 12,7 | 10,7 | 0,9 | 40,9 | 107,8 | 1,1 | 5,1 | 4,1 | 0,6 | 4 | 2 | 0 | 0 | 3 | 0 |
| Ala 0065 | 16,8 | 15,5 | 1,2 | 52,7 | 193,6 | 1,1 | 7,6 | 6,1 | 0,6 | 1 | 1 | 1 | 3 | 0 | 0 |
| Ala 0066 | 10,9 | 7,7 | 0,8 | 33,3 | 65,2 | 1,2 | 4 | 2,1 | 0,7 | 5 | 1 | 0 | 0 | 5 | 0 |
| Ala 0071 | 18,3 | 17 | 1,4 | 60,4 | 235 | 1,3 | 7,6 | 6,3 | 0,5 | 1 | 1 | 1 | 5 | 0 | 0 |
| Ala 0072 | 19,6 | 17,4 | 1,5 | 65,5 | 270,9 | 1,1 | 9 | 7,5 | 0,5 | 6 | 1 | 1 | 5 | 1 | 0 |
| Ala 0073 | 22 | 16,3 | 1,6 | 67,9 | 306,3 | 1,1 | 7,3 | 6 | 0,5 | 2 | 2 | 0 | 0 | 0 | 0 |
| Ala 0074 | 23,8 | 18,1 | 1,8 | 77,6 | 383,5 | 1,1 | 9,3 | 7,5 | 0,6 | 6 | 1 | 1 | 5 | 1 | 0 |
| Ala 0075 | 18,2 | 15,2 | 1,3 | 57,3 | 188,8 | 1,2 | 8,1 | 6,1 | 0,6 | 4 | 1 | 1 | 2 | 3 | 2 |
| Ala 0077 | 18,9 | 14,8 | 1,4 | 61,9 | 212 | 1,2 | 7,8 | 5,8 | 0,6 | 6 | 1 | 1 | 8 | 1 | 1 |

**Table 15: Eigenvalues and percentages of variation, as explained by the principal components (PC) for each of the 15 variables analyzed**.

| **PC** | **Eigenvalue** | **% variance** |
| --- | --- | --- |
| 1 | 6,60013 | 44,001 |
| 2 | 2,02474 | 13,498 |
| 3 | 1,5211 | 10,141 |
| 4 | 1,21551 | 8,1034 |
| 5 | 0,967802 | 6,452 |
| 6 | 0,873181 | 5,8212 |
| 7 | 0,603877 | 4,0258 |
| 8 | 0,42512 | 2,8341 |
| 9 | 0,305715 | 2,0381 |
| 10 | 0,259293 | 1,7286 |
| 11 | 0,109722 | 0,73148 |
| 12 | 0,0438231 | 0,29215 |
| 13 | 0,0343857 | 0,22924 |
| 14 | 0,00899936 | 0,059996 |
| 15 | 0,00660266 | 0,044018 |

Variable names: 1: Maximum length of the starch grain, 2: Minimum length of the starch grain, 3: Elongation index, 4: Total perimeter, 5: Total area, 6: Compactness Index, 7: Maximum distance between the hilum and edge, 8: Minimum distance between the hilum and edge, 9: Centricity Index, 10: 2D Shape, 11: Hilum type, 12: Hilum fissure, 13: Shape of the hilum fissure, 14: Facets, 15: Lamellae, and 16: Cross style.
