# Supplementary material for: Identification of breadfruit (Artocarpus altilis) and South American crops introduced during early settlement of Rapa Nui (Easter Island), as revealed through starch analysis
Source: PLoS One. 2024 Mar 20;19(3):e0298896. doi: 10.1371/journal.pone.0298896 (PMC10954183; doi:10.1371/journal.pone.0298896)
Supplement: S1 Text — (DOCX) [file pone.0298896.s006.docx]

**Description of the characteristics of morphotypes associated to species in the reference collection.**

1. **Traditional Polynesian plants**

*Artocarpus altilis*. Starch granules are slightly larger than those of *C. esculenta*; up to 12 µm in size. The most common shape is polyhedric or polymorphic, with multi-flat and concave facets. Arrays of compound clusters are also present. The hilum is mostly closed, with no fissure. Wrinkly interior. The extinction cross has straight arms.

*Colocasia esculenta*. Starch granules are generally under 10 µm long. The most common shape is polyhedric, flat multi-faceted. Granules form compound clusters and have mostly no fissured hilum. The extinction cross has straight arms.

*Curcuma longa*. Oblanceolate-shaped granules around 30 µm in length, on average. The hilum almost always is not visible; therefore, no fissures are found. Absence of pressure facets. The extinction cross has wide curvy arms.

*Dioscorea alata*. Mostly large single granules around 30 µm long, on average, with oblong shapes with no pressure facets. The hilum is open, with no fissure. The extinction cross style has very distinct wavy arms.

*Inocarpus fagifer*. Mostly oval or bell-shaped granules, 8-16 µm in length, with 1-3 multi-flat-facets and closed, large, and circular fissured hilums. Clusters of compound granules are present. The extinction cross has straight arms.

*Musa sp*. Large oblanceolate-shaped granules around 45 µm in length, on average. Open eccentric hilum with no fissures. Both multi-flat and multi-concave pressure facets are present. The extinction cross has wide very distinct curved arms.

*Spondia dulcis*. Mostly oval-shaped granules, 9-24 µm in length. Open hilum with simple (lineal), or “v”-shaped large fissures. No pressure facets are observed. The extinction cross has distinct curved arms.

*Zingiber officinale*. Oblanceolate-shaped granules that range between 10-40 µm in length. Closed hilum with no fissures. Pressure facets are absent. The extinction cross has curved arms.

**South American crops**

*Canna* sp. Single granules, with oval, oblong, or oblanceolate shapes, 15-55 µm in length. The hilum is eccentric, closed, and barely visible. The extinction cross is almost always eccentric, with wavy or straight arms. No pressure facets are observed.

*Ipomoea batatas*. Mostly oval or polyhedric shapes with 1-4 multi-mixed facets that are irregular in size and shape. Slightly open and excentric hilum, generally with no fissures. Granules that present hilum fissures were mostly “large” or “hat-like” shaped.

*Manihot esculenta*. Circular, oval, or bell-shaped granules, 9-22 µm in length. Starches appear single or as clusters of compound granules. Mostly open hilum with no fissures. Granules with fissures are simple, lightly curved, or “Y”-shaped, with mixed pressure facets. The extinction cross has straight arms.

*Xanthosoma sp*. Granules between 12 and 20 µm in length, mostly bell-shaped but oval or polymorphic shapes are also present. Hila are open and fissured, normally “hat-like”- or “large”-shaped fissures are present. The extinction cross has straight arms.

1. **Control species**

*Triticum aestivum*. Large and small oval-shaped granules, 10-35 µm in length, on average, with pressure craters on the surfaces that appear as small depressions. Open hilum, absence of fissures and pressure facets**.**
